# Machine Learning for Automatic Prediction of the Quality of Electrophysiological Recordings

**DOI:** 10.1371/journal.pone.0080838

**Published:** 2013-12-04

**Authors:** Thomas Nowotny, Jean-Pierre Rospars, Dominique Martinez, Shereen Elbanna, Sylvia Anton

**Affiliations:** 1 Sussex Neuroscience and Centre for Computational Neuroscience and Robotics, University of Sussex, Brighton, United Kingdom; 2 Physiologie de l’Insecte: Signalisation et Communication, Institut National de la Recherche Agronomique, and Université Pierre et Marie Curie, Versailles, France; 3 Laboratoire Lorrain de Recherche en Informatique et ses Applications, Centre National de la Recherche Scientifique, Vandœuvre-lès-Nancy, France; Monell Chemical Senses Center, United States of America

## Abstract

The quality of electrophysiological recordings varies a lot due to technical and biological variability and neuroscientists inevitably have to select “good” recordings for further analyses. This procedure is time-consuming and prone to selection biases. Here, we investigate replacing human decisions by a machine learning approach. We define 16 features, such as spike height and width, select the most informative ones using a wrapper method and train a classifier to reproduce the judgement of one of our expert electrophysiologists. Generalisation performance is then assessed on unseen data, classified by the same or by another expert. We observe that the learning machine can be equally, if not more, consistent in its judgements as individual experts amongst each other. Best performance is achieved for a limited number of informative features; the optimal feature set being different from one data set to another. With 80–90% of correct judgements, the performance of the system is very promising within the data sets of each expert but judgments are less reliable when it is used across sets of recordings from different experts. We conclude that the proposed approach is relevant to the selection of electrophysiological recordings, provided parameters are adjusted to different types of experiments and to individual experimenters.

## Introduction

Electrophysiological recordings are widely used to evaluate how nervous systems process information. Whereas up to about two decades ago rather small data sets were acquired which were easy to analyse manually, a rapid development of data acquisition and storage techniques now allow to accumulate huge datasets within a relatively short time and their analysis is often highly automated. This trend tends to further accelerate with the introduction of automated electrophysiology in ion channel discovery [1]–[3]. Nevertheless, an experienced electrophysiologist will typically still examine recordings by hand, one by one, to evaluate which recordings in a dataset will be suitable for exploitation by automated analyses. This practice can be problematic in several ways. Because it is based upon human judgement on a case-by-case basis, data selection by manual inspection is liable to selection or sampling bias; that is, a statistical error due to the selection of a limited, non-representative, sample of the full neural population. Although some statistical techniques aim at correcting for the small number of recordings [4], the reliability of the selected data remains problematic [5]. Different experimenters may select or reject different recordings and their decisions can depend on context, e.g. if a lower quality recording occurs among many very high quality ones or among other low quality recordings.

A secondary problem with manual data inspection is the sheer effort that is needed to classify large data sets. With “easy” experimental protocols, a strategy to keep only rapidly recognizable “good” recordings can be used, but with complex experimental protocols it is often time consuming to judge each recording trace “by eye” and errors in the judgement can lead either to a loss of recordings (if judged not sufficient although they might be analysable), errors in results (if judged analysable although they lack quality and hence lead to errors in the results) or a waste of time (if judged analysable, but their quality proves insufficient during analysis).

Many aspects of data analysis have undergone a process of automation starting from filters [6] to spike detection [7] and sorting [8]–[10], and from feature analysis of spike responses [11], [12], and inter-burst interval detection [13] in EEG recordings to statistical analysis and visualisation [14].

However, the final judgement whether to include a recording into the analysis or reject it as too low in quality or artefactual is still reserved to the human researcher. Here, we begin to challenge this established practice.

To facilitate the choice of electrophysiological recording traces for further analysis, and remove subjectivity from this process, we propose an automated evaluation process based on machine learning algorithms using examples of intracellular recordings from central olfactory neurons in the insect brain.

In machine learning [15], in contrast to alternative, automated expert systems [16], there is no rule-based decision for deciding the class of an input, e.g. the quality of a recording, but the distinction between classes are derived from examples. Rather than setting specific limits on features like the spike height, width and noise amplitude, examples of values of these features are made available to the machine learning system together with the correct classification of the recordings and the system extrapolates from the examples to decide on new inputs. In this work we define 16 characteristics (features) of electrophysiological recordings and encode a large number of recordings by the value of these 16 features as 16-dimensional feature vectors. The recordings are classified by an experienced electrophysiologist into three classes of “good” (can be used for analysis), “intermediate” (may be used for analysis but there are problems) and “bad” (not suitable for further analysis). A subset of the recordings is then used to train the machine learning classifier and this classifier can then be used to predict the classification of the remaining or new recordings.

An important ingredient for successful application of machine learning methods is feature selection [17], [18]. It is well established that for solving any particular problem, like the classification of recording quality addressed here, it is important to only use the features that are most relevant to the specific problem. Including additional, non-relevant features into the process will degrade the ability of the classifier to generalize to novel examples. However, for any given problem, the optimal number and identity of features are typically unknown. In this paper we use a so-called wrapper method [19], [20] of feature selection to determine the relevant features: the classifier is trained and tested in cross-validation [21], [22] on all possible choices of features in a brute force exploration; the best combinations of features is then used in the final classifier.

## Methods

### Data Sets

We used two data sets within the numerical analysis in this work. Data set 1 was acquired by one of the authors (“expert 1” in what follows) and combines recordings from central olfactory neurons in the antennal lobe of the noctuid moths *Spodoptera littoralis* and *Agrotis ipsilon*. Data set 2 was acquired by another author (“expert 2” in what follows) and contained similar recordings from *A. ipsilon*. Data set 1 consists of 183 recordings and data set 2 of 549 recordings.

All recording traces were obtained with attempted intracellular recordings with sharp glass electrodes of central olfactory neurons within the antennal lobe of the two moth species. Each recording trace was approximately 5 s in duration. A species-specific sex pheromone stimulus (varying doses for different traces) was applied 1.5 s after the onset of the recording for 0.5 s.

For the purpose of this work on automatic data quality assessment, the ground truth for the data quality of the used recordings was established by visual inspection by two experts. They classified the recordings in 3 categories: “bad”, “intermediate” and “good” as defined in the introduction. Expert 1′s classification resulted in 29 bad, 54 intermediate and 100 good recordings for data set 1 and 90 bad, 130 intermediate and 329 good recordings for data set 2. Expert 2′s classification resulted in 20 bad, 39 intermediate, and 124 good recordings in data set 1 and 178 bad, 75 intermediate, and 296 good recordings in data set 2. Exemplary plots of the data are provided in Data S1.

### Candidate Features

In order to enable a machine classifier to make decisions about the quality of recordings it needs access to the relevant properties of the data. We defined 16 such properties that we call features.

The data are first pre-processed with filters and a rule-based spike detection algorithm. If recorded with different gain factors during data acquisition, the recorded membrane potential *V*(*t*) was multiplied by the gain factor to achieve a common scale for all recordings (e.g. mV). For the purpose of spike detection, *V*(*t*) was then filtered with a moving average of window size 3 ms. Candidate spike events were detected based on two threshold criteria on the derivative of the filtered membrane potential. Detecting spikes based on the derivative will automatically remove any occurring plateau potentials and possible recording artefacts due to current injections. In order to qualify as a candidate spike event, 3 consecutive derivatives need to be above the upward threshold θ_up_ and within *t*_spike,max_ = 3 ms, 3 consecutive derivatives need to be below the downward threshold θ_down_. These conditions test for the sharp rise and fall of the membrane potential around a spike and are independent of the overall amplitude and baseline values. The upward and downward derivative thresholds θ_up_ and θ_down_ were chosen as three times the 80^th^ percentile and 3 times the 20^th^ percentile of all observed values of the derivative, respectively. Deriving the threshold values from percentiles of observed values of the derivative helps us to take into account if a sufficient number of spikes are less steep due to current injections or other prolonged excitation. Our manual controls of spike detection showed that this strategy is vey reliable in finding all candidate spike events.

**Figure 1:**
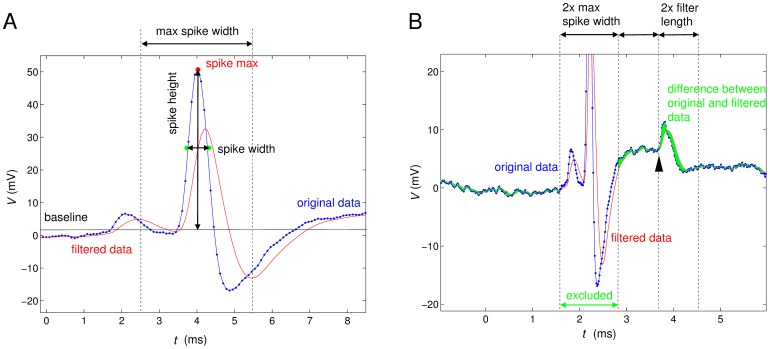
Illustration of the calculation of spike height, spike width and noise amplitude. A) Spike height and width: The blue trace represents the original voltage data with small blue markers indicating the sampling. The red line is the moving average, which is used in spike detection. The black horizontal line represents the baseline value that is calculated by averaging the membrane potential in windows to the left and the right of the spike. The spike height is determined as the difference of the maximal voltage value of the spike and the baseline value. The spike width is measured as the distance of the two closest measurements below the half-height of the spike. B) Short time scale noise amplitude: The difference is taken between the original membrane potential measurement *V*_m_ and the filtered membrane potential measurement *V*_avg_ (moving average, see Figure 1A), and its Euclidean norm (normalised by 2 times the filter length +1) is calculated over two filter lengths, 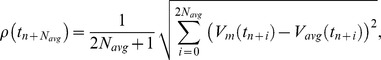 (1) e.g. the noise level at the arrowhead is calculated from the marked interval of 2 filter lengths. The areas of 2 maximal spike widths (2×3 ms) around every detected spike are excluded from this calculation and the local noise level is undefined in these areas around spikes.

Figure 1 illustrates the characterization of spike features (Fig. 1A) and local noise (Fig. 1B). The maximum of the candidate spike was calculated as the maximum of *V*(*t*) (“spike max” in Fig. 1A) between the first crossing of the derivative above θ_up_ and the last point where the derivative was below θ_down_ and the time when this maximum is attained defines the spike time *t*_spike_. The local baseline value around the candidate spike was calculated as the average membrane potential *V*(*t*) in the intervals [*t*_spike_ –6 ms, *t*_spike_ –3 ms] and [*t*_spike_ +3 ms, t_spike_ +6 ms] (black horizontal line in Fig. 1A). The spike height is then given by the difference between the maximum membrane potential and this local baseline.

To eventually be accepted as a spike event, some additional conditions have to be met: (i) there are points within 3 ms before and after the spike time where the membrane potential is lower than the half height of the spike, and (ii) the spike height has more than half the 95^th^ percentile of all observed spike heights. The first rule excludes certain artefacts, where there is mainly a step up or step down of the recorded potential and the second rule excludes small secondary events like secondary spikes from other neurons (Note, that even though these are intra-cellular recordings, spikes from other neurons can potentially be present due to either strong coupling by gap junctions, recording electrodes contacting more than one cell or the existence of multiple spike initiation zones [23], [24].) or mere EPSPs.

The 16 features of a recording that we consider in the following are based on the detected spike events as well as on general properties of the full bandwidth *V*(*t*) signal. They are summarized in table 1 and Figure 1A,B. Automatic feature extraction was performed with Matlab (Mathworks, Natick, MA) and takes about 21 minutes for the larger of our data sets (549 recordings). The Matlab tools for automatic feature extraction are provided as supplementary material (Toolbox S1).

**Table 1:** Full set of considered features of recordings.

	Feature name	Definition/Method of calculation
1	Mean spike height	Spike heights are calculated for each spike event as described above as the difference between the maximum value and the local baseline.
2	Mean spike width	The width of spikes is determined as the time difference between the two closest points before and after *t*_spike_ that are less than half of the spike amplitude above local baseline.
3	CV of spike height	Standard deviation of spike height divided by the mean spike height.
4	CV of spike width	Standard deviation of spike width divided by the mean spike width.
5	Mean baseline voltage	Average of all values of the filtered membrane potential that lie between the 5^th^ and 95^th^ percentiles, assuming that this will capture typical values outside spike events.
6	Std of baseline	Standard deviation of the filtered membrane potential values that lie between the 5^th^ and 95^th^ percentiles.
7	Short timescale noise	We calculate the Euclidean distance between the membrane potential values and the filtered membrane potential in time intervals of twice the filter length of 3 ms (illustrated in Fig. 1B as the green area). For the purpose of this calculation, sections of the membrane potential that constitute detected spikes are omitted (“excluded” in Fig. 1B). The value of this distance at each time point is interpreted as the short timescale noise at this time. We then take the mean value across the recording.
8	Std of noise level	Standard deviation of the short timescale noise across the recording.
9	Drift of spike height	Slope of a linear regression for the spike height as a function of the spike time. The slope of this regression is interpreted as a drift in the spike height over time, which may be caused by deteriorating recording quality.
10	Drift of spike width	Slope of a linear regression for the spike width as a function of the spike time. Non-zero values can be interpreted as an indicator of decreasing health of the recorded neuron.
11	Drift of noise amplitude	Slope of a linear regression on the short timescale noise as a function of time, which we interpret as a drift of the noise amplitude reflecting whether the recording quality may be measurably decreasing (or increasing) over the duration of the measurement.
12	Minimum ISI	Minimal inter-spike interval (ISI) between all confirmed spike events.
13	Maximum spike slope	Mean of the maximal values of the derivative of the filtered voltage around each spike (between threshold crossings).
14	Minimum spike slope	Mean value of the steepest decline of the filtered membrane potential during the falling phase of each spike (between threshold crossings).
15	Std of maximum spike slope	Standard deviation of the maximum slopes of all observed spikes.
16	Std of minimum spike slope	Standard deviation of the set of values observed for the minimum slope around each spike.

### Distributions

The statistical distributions of feature values are shown as histograms (20 bins) in a range that includes the 5^th^ to the 95^th^ percentile of observed values (i.e. excluding extreme outliers if there are any). Statistically significant differences between distributions were determined by Kolmogorov-Smirnov tests with Bonferroni correction for multiple pairwise tests at 5% and 1% significance levels.

### Crossvalidation and Classification Method

Crossvalidation is used to assess the success of a classification method when no separate test set is available. The data set of interest is split repeatedly into a training and testing portion and the performance of the classification algorithm is assessed on these different splits. We use 10-fold crossvalidation, in which the data is split into a 90% training set and 10% left out samples for testing. The split is chosen randomly, but such that after 10 repeats, all samples have been left out once. If not stated otherwise we repeat the full 10-fold crossvalidation 50 times with independent random splits.

As classifiers we used linear support vector machines (SVMs) [25]. SVMs are known to perform competitively in a number of applications. We decided to employ a linear SVM to avoid introducing additional meta-parameters such as a kernel degree or parameters of radial basis functions and, importantly, to limit the risk of over-fitting, which is higher in non-linear SVMs when the data is high dimensional. To avoid infinite iterations, which occur in rare cases with minimal features (which arguably are not even particularly interesting), we limited the learning iterations of the SVM algorithm to 10^4^ steps. We checked with unlimited learning iterations (with a ceiling of 10^6^ iterations in two rare cases where otherwise apparently infinite iterations occurred) and observed no discernible differences in the results.

For the cost parameter of the linear SVM we used C = 512. Repeated runs with C = 8, 32, and 128 gave similar results.

### Wrapper Approach to Feature Selection

The wrapper approach to feature selection is a brute force method in which all possible choices of features are tested with respect to the performance of classification in crossvalidation based on each feature choice. Here, there are 16 possible features allowing 2^16^ – 1 potential choices for the group of used features. We call a particular choice of features, e.g. features (1, 4, 9, 11) a feature set and the number of employed features, 4 in this example, the size of the feature set. Most results will be reported separately for feature sets of different sizes.

The wrapper features selection was executed on the in-house computer cluster of the University of Sussex in separate processes for each feature set size. Computation times varied from 20 s for feature sets of size 16 to about 3.5 days for feature sets of size 7–9.

The normal wrapper approach of feature selection with crossvalidation is prone to the following over-fitting effect: we typically test all possible feature sets in crossvalidation and then report the best observed performance and identify the feature set that obtained this performance. If this best-performing feature set is interpreted as the optimal feature choice for the problem at hand one is exposed to the selection bias of potentially identifying a feature set where the crossvalidation procedure (which contains a random element) worked particularly well by chance. This bias is particularly strong when a large number of feature sets with very similar quality are compared. To avoid this bias and, without using truly novel test data, get a realistic estimate of how well one would do when using a wrapper method for feature selection, we devised a two-stage-leave-one-out crossvalidation procedure. In the first stage, one recording is left out and the remaining training set of *n*−1 recordings is used for a full wrapper feature selection with crossvalidation. This involves choosing feature sets and evaluating them in crossvalidation, i.e. leaving out another recording, training a classifier on the resulting training set with *n−2* recordings and testing it on the left out recording. For full crossvalidation, this procedure is repeated until all *n−1* recordings have been left out. We then use the top10 best feature sets (see below for a definition of the top10 group) and train corresponding classifiers on the “full” training set of n−1 recordings. The resulting classifiers are then finally used for predicting the class of the originally left out recording. This procedure is repeated until all recordings have been left out once in stage one.

## Results and Discussion

### Statistical Distributions of Feature Values in the Data Sets

**Figure 2:**
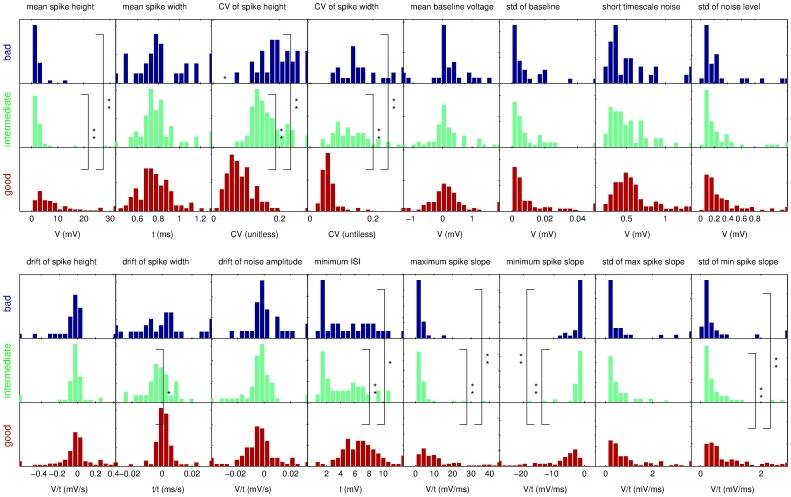
Overview of the observed distributions of feature values for data set 1. The value distributions are shown separately for recordings that were classified as good (red), intermediate (green) or bad (blue) by expert 1. We have compared the distributions with Kolmogorov-Smirnov tests and found that many but not all distributions differ significantly on significance level α = 0.05 (one star) or α = 0.01 (two stars). We note that the distributions between intermediate and bad recordings rarely differ significantly but often both do differ from the good recordings.

**Figure 3:**
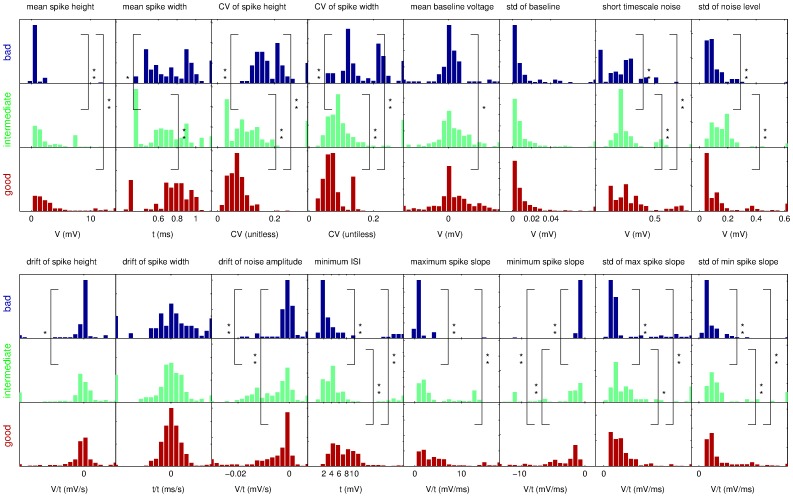
Overview of the observed distributions of feature values for data set 2. The conventions are as in Figure 3. We note that for this data set the differences of feature value distributions are even more pronounced.

We calculated the values of all 16 features on the two data sets and plotted the distribution of observed values separately for each group of bad (blue), intermediate (green) and good (red) recordings, as judged by expert 1 (Fig. 2, 3). The quantities plotted are noted in each graph. The plots report relative occurrence within each group rather than absolute numbers to take into account the different group sizes (100 good, 54 intermediate, and 29 bad recordings in data set 1 and 329 good, 130 intermediate and 90 bad in data set 2).

The plots reveal noticeable differences between distributions of feature values for good recordings and intermediate or bad recordings, e.g. for the mean spike height or the CV of the spike height. To test this observation formally, we performed pairwise Kolmogorov-Smirnov tests with a Bonferroni correction for multiple statistical tests on a 5% significance level (one star in Figures 2 and 3) and 1% significance level (two stars in the Figures). We find that many features show significant differences between the distributions for good and bad recordings and between distributions for good and intermediate recordings. The main features with highly significant differences in both data sets are spike height, CV of spike height, CV of spike width, minimum and maximum spike slope, and standard deviations of minimum and maximum spike slope (see relevant panels in Fig. 2, 3).

The distributions for bad and intermediate recordings rarely differ significantly but more so in data set 2 than in data set 1. It is worth noting that the different numbers of recordings for the three categories imply that the power of the KS test will be different for the various comparisons and hence in some cases differences between intermediate and bad recordings are not significant even though they are visible to the eye (Fig. 2, 3).

**Figure 4:**
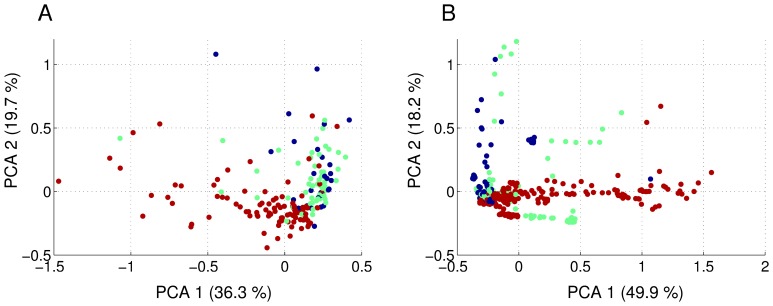
Principal component analysis in the 16 dimensional feature space. The plots display the first two principal components of all considered recordings in data set 1 (A) and data set 2 (B). Each point represents one recording and is colour coded as a good (red), intermediate (green) or bad (blue) recording, according to evaluation by researcher 1. We note that there is a systematic structure to the clouds of points representing the three recording qualities but the clusters are not fully separated.

To further analyse whether the observed differences in the distributions of feature values for the three different categories result in a clear cluster structure in the 16 dimensional feature space that would be amenable to standard machine learning algorithms for classifying new recordings of unknown quality, we performed a principal component analysis. The results are illustrated in Figure 4. The two first principal components account for 36.2% and 19.7% of the total variance in data set 1 and 49.9% and 18.2% for data set 2, indicating that data set 2 has a lower-dimensional structure in the 16 dimensional feature space that can be captured more easily in two principal components.

We observe some visible differences between good recordings and the rest, but clusters are not particularly well defined in either data set. Especially intermediate and bad recordings seem very intermingled (Fig. 4). This suggests that building a machine learning system for automatic detection of recording quality may not be trivial.

From the statistical analysis we can conclude that most of the chosen features are informative for distinguishing the quality of recordings. We would expect that when assessed for their suitability in a machine learning approach to predicting recording quality the features that gave rise to the most significant differences between recordings of differing quality would be the best candidates. We will re-examine this question in the framework of a wrapper feature selection method below.

### Feature Selection and Classification

The problem of not well-separated classes is common in machine learning and one of the most important elements of a successful machine learning system is the selection of a subset of the most relevant features and the omission of the less informative or misleading ones. Here we used a standard approach to this problem, a so-called wrapper method. In brief, in a wrapper approach to feature selection all possible subsets of features are tested with the employed classifier on the training set, usually in crossvalidation (see Methods). The feature set with the best prediction performance in crossvalidation is chosen for the final classifier, which is then tested on a separate test set.

**Figure 5:**
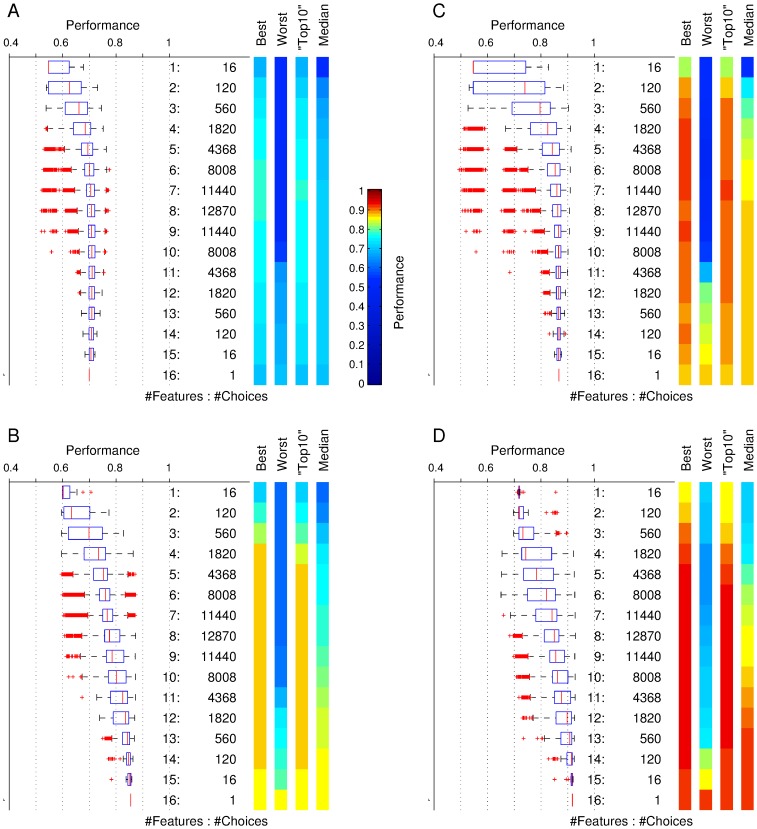
Crossvalidation performance as a function of the number of chosen features. The boxplots represent the distribution of observed fraction of correct classification for all feature choices of *n* features, with *n* ranging from 1 feature (top line) to all 16 features (bottom), see labels in the middle column. The colour bars indicate the performance for the best, worst, top10 (see main text for definition) and median feature choice. Panel A and C show the performances for classifying all three categories of good, intermediate and bad for data set 1 (A) and data set 2 (C). Panels B and D show the results for classifying “good” against the combined category of “intermediate or bad”.

We performed wrapper feature selection on all possible subsets of the 16 defined features, a total of 2^16^–1 = 65535 possible selections. As a classifier we used a linear support vector machine (SVM). Figure 5 shows the performance for the two data sets. Performance values are grouped by the size of the employed feature sets, i.e. size 1 indicates only one feature was used and size 16 means that all features were used. The performance of the classifier is expressed as the percentage of correct predictions, i.e., 90% performance would mean that the classifier predicted for 90% of the recordings the true quality value (as provided earlier by expert 1). We here report the best performance for any of the feature choices, the worst observed performance, the median of the observed performance values and the average performance of the “top10” group of choices (see vertical colour bars in Figure 5). The top10 group is defined as the 10 best performing choices, or, for smaller numbers of overall available choices (e.g. when choosing 15 out of 16 features, etc.), the performance of the best 10% of choices, but always of at least one choice. Using 10-fold crossvalidation we observed that in data set 1 (Fig. 5A) the best performance was maximal when using 6 features and led to 77.5% correct predictions. The maximal average performance of the “top10” groups was 76.8% when using 7 features. In data set 2 (Fig. 5C) we find optimal performance of 87.4% when using 8 features and best average performance of the top10 group of 87.1% when using 10 features. This compares to the following chance levels: In data set 1, for fully random guesses of equal probability, the expected performance would be 33.3%, for guessing proportional to the abundance of the three classes, 41.1% and if guessing that recordings are always of class “good”, 55%. In data set 2 the corresponding chance levels are 33.3% (random), 44.2% (proportional) and 60% (guessing “good”).

For data set 1, the set of features that was performing best consisted of 6 features: (1, 4, 8, 12, 13, 16), and for data set 2 of the 8 features (1, 2, 3, 4, 5, 13, 14, 15). We note that the spike height (feature 1) and the CV of the spike width (feature 4), as well as, the maximum spike slope (feature 13) are common to both feature sets. Comparing to the statistics shown in Figures 2 and 3, the distributions of feature values for these three features do show visible and significant differences in both data sets. Conversely, the standard deviation of the baseline is not a chosen feature in either case, which appears to correspond with the observation that the value distributions for this feature are not very different between classes. Beyond these obvious observations, however, it is hard to predict by manual inspection of the value distributions which combination of features may be particularly successful, necessitating the exhaustive wrapper approach.

To further test the idea that features 1, 4 and 13 are particularly useful, we inspected the performance of all feature sets that contain all three of these and compared them to the performance of feature sets that do not contain them all. We observe that for data set 1 and feature sets with more than 4 and less than 14 elements, the average performance of the sets containing the three features is significantly higher than of the sets not containing all of them (1-way unbalanced ANOVA, P<10^−4^ or less). For data set 2, however, we do not see such a significant effect, even though the performance of the feature sets containing all three features visually appears higher for most feature set sizes (data not shown). Finally, on their own, the three features lead to a performance of 68.1% (3 classes), 87.1% (2 classes) in data set 1 and 76.7% (3 classes), 78.3% (2 classes) in data set 2.

**Figure 6:**
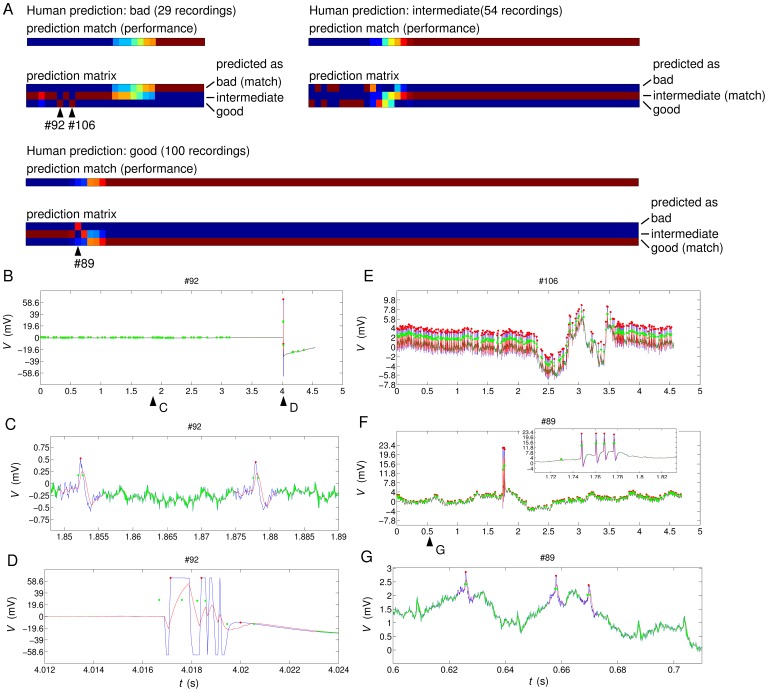
Comparison of individual predictions of expert 1 and the machine learning classifier 6–2133. (The feature sets are labeled by their size, here 6 features, and then enumerated from 0 to N–1, where N is the maximal number of feature choices for the given size. The feature set used here is number 2133 out of a total of 8008 possible choices.) for data set 1. A) Match of the predictions of human expert and machine. The recordings are ordered from the recording with the worst mismatch between expert 1 and the 50 times repeated 10-fold crossvalidation with 6–2133 on the left to the ones with the least mismatch on the right. The colour represents the percentage of crossvalidation runs where machine and human prediction match scaling from blue (0% match) to dark red (100% match). The 3-row prediction matrices show the details of how often individual recordings (x-axis) were recognised as the three classes (y-axis). The colour scale is the same as for the performance. B)–G) Raw data plots of the three most problematic recordings, where blue lines are the voltage data, red lines the filtered voltage data, red dots the top of detected spikes, and green dots the half-height of spikes, shown at spike time +/− half spike width. Panels C and D are detailed plots of two relevant regions of recording #92 as indicated by arrowheads in B. The inset in F and panel G are details of two relevant regions of recording #89 as indicated by arrowheads in F.

When analysing the errors being made by the classifier using the above best feature choice of size 6 (Fig. 6) we note that there are 3 specific recordings for which predictions are opposite to human judgement (i.e. predicting “bad” when the ground truth was “good” or predicting “good” when it was “bad”) and consistently so across repeated crossvalidation runs (Fig. 6A, arrowheads). We inspected these recordings (#89, #92 and #106 in our data set 1) manually (Fig. 6B–G) and found clear reasons for the discrepancy that elucidate the remaining limitations of the automated system.

Recording #92 contains a large artefact and no obvious spikes; it is therefore classified as “bad” by the human expert. The preprocessing picked up both the artefact and many small, but very consistent spikes (Fig. 6 C,D). Because of the reliance on percentiles (aimed at limiting the impact of potential artefacts), the recording is predicted to be good by the machine based on the many small spikes.

Recording #106 also contains artefacts, in this case an unstable baseline voltage that might compromise reliable spike detection (Figure 6E). It was therefore judged to be “bad” by expert 1. However, the automatic system detects spikes quite reliably and of consistent amplitude and because the standard deviation of the baseline (feature 6) is not included in the feature set employed here, the automatic system classifies the recording as “good”.

The third example, recording #89, contains four clear spikes (Fig. 6F, inset) and was judged to be good by expert 1. However, the automatic system detects secondary, very small spikes (Figure 6G), which are not excluded by the spike eligibility rules (see Methods) because those are based on percentiles and four large spikes are not sufficiently many to trigger the exclusion rules for the spike height. As a result, the calculated features have large values for the standard deviations of spike height and spike width, the latter of which is included in our best-performing feature set – a likely reason for the observed “bad” classification.

Apart from the three discussed examples, all other mistakes are between good and intermediate or between bad and intermediate, the latter ones being about twice as frequent. This is consistent with the observed differences in the statistical distributions of feature values and suggests that the distinction between intermediate and bad recordings is the most difficult. Accordingly, if we combine intermediate and bad recordings into one class of unacceptable recordings and ask the same question of classifying the data quality but now only into the two categories “good” and “unacceptable”, we observe much higher classification success (Fig. 5B,D).

Overall success rates of almost 80% for the three class problem and over 90% for the two class problem make the automatic system attractive for research areas where high volumes of data need to be processed. Furthermore, the low error rate between the extremes of good and bad makes the system fairly safe to use. One strategy of using it would be to rely on the two class system and keep all recordings with a “good” rating. Alternatively, one could additionally use the three class system to identify candidates for intermediate quality and manually inspect them to maximise the usable recordings. Optimally one would want to design a system that is particularly geared towards identifying the distinction between “intermediate” and “bad”; however, the statistics for the feature values (Fig. 2,3) and our classification results indicate that this is the hardest part of the problem.

### Feature Use Statistics

**Figure 7:**
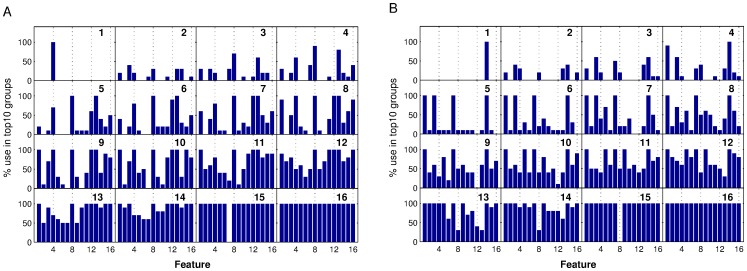
Distributions of the occurrence of individual features in the top10 feature groups for each given size of the feature set from 1 (top left panel) to 16 (bottom right panel) features. If a single feature is used, feature 4 (CV of spike width) is best for data set 1 (A) and feature 14 (minimum spike slope) for data set 2 (B), but when using 2 or more features, combinations involving other features work best. The highly successful feature sets between 5 to 10 features (second row) show some commonalities in that for data set 1 (A) features 8 (std of noise level) and 13 (maximum spike slope) are always included and for data set 2 (B) features 1 (mean spike height), 3 (CV of spike height), 8 (std of noise level) and 14 (minimum spike slope) play a dominant role. It is noteworthy that observations reported here do not seem to generalize well between the two data sets used in this study.

Having tried all possible combinations of features we now can ask which features are the most useful for the classification of recording quality. We built the distribution of how often particular features where used in the ‘top10’ groups in the experiments with data sets 1 and 2. The results are illustrated in Figure 7.

Interestingly, the most successful features differ for the two data sets, indicating that individual feature selection may be necessary for different experimenters, and likely also for different types of experiments (see also ‘testing across data sets’ below). However, the distributions do have in common that for small feature sets of 2–4 features, there are no clear preferences but many combinations of features seem to work similarly well. This appears to indicate that several of the defined features are informative for the quality of the recording and there is no one golden rule deriving from only one or two central features. For larger feature sets of 5 to 8 features we notice that features (4, 8, 12, 13) seem to (almost) always be used but with different combinations of other features, indicating that these features seem to be the most salient for the task.

Overall the wide spread of features used indicates, however, that there are less dominant features in this application of machine learning than in other domains [18]. Furthermore, the difference in successful features for data set 1 and data set 2 might indicate that the best features may depend on the experimenter and, to speculate a bit further, likely on the nature of the preparation and the experiments. The use of an automatic procedure for both feature selection (wrapper method) and classification however alleviates this problem as a data quality system could be fairly quickly adjusted to novel experiments or preparations, simply by providing a well-sized set of examples for different quality recordings and the appropriate class labels based on human judgement. From there on, the procedure can be fully automated.

### Testing Across Data Sets

In a practical application one would choose features and train a classifier on a reference data set to then automatically recognise the recording quality in future recordings. To investigate the performance of our machine learning system with wrapper feature selection in this situation we choose the best-performing feature sets in crossvalidation on data set 1, train a classifier for the best and the top10 feature choices on data set 1 and classify all recordings in data set 2 using the resulting classifiers.

**Figure 8:**
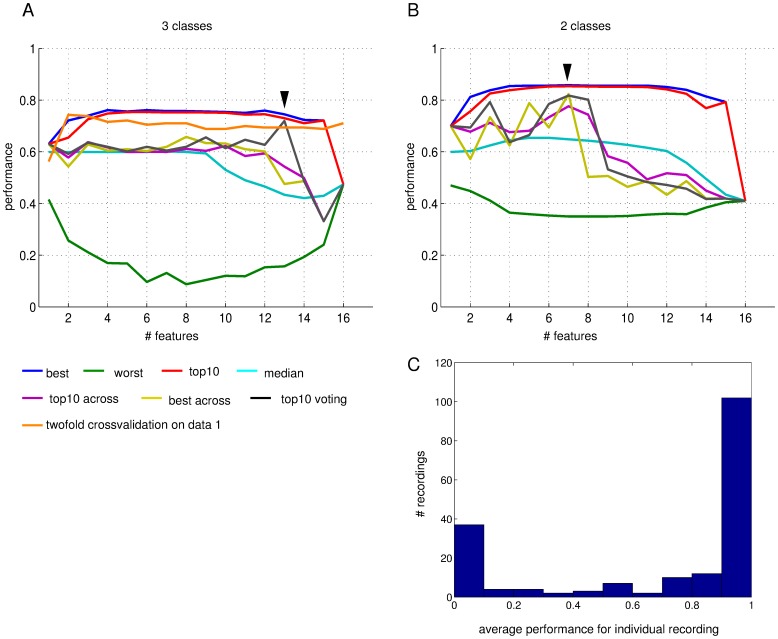
Performance of training classifiers and choosing features on data set 1 and predicting the quality of recordings in data set 2 for the 3-class problem (A) and the 2-class problem (B). The most relevant data are the performance of choosing the best feature set as observed in crossvalidation on data set 1 (blue), training a classifier using this feature set on data set 1 and then predicting the qualities in data set 2 (“best across”, yellow), the average results of doing so with the members of the top10 group as determined on data set 1 (“top10 across”, purple) and the result of using a voting scheme within the feature sets of the top10 group (“top10 voting”, black). Note that for three classes the voting mechanism delivers good performance for 13 dimensional feature sets, while for the 2-class problem 6–8 features appear to be optimal and provide good performance (arrowheads). The red lines show the performance of the top10 feature sets in normal crossvalidation on data set 1. And the green line of the worst feature set. The orange line shows the observed performance of a top10 group of feature sets that are chosen and tested in a two-fold crossvalidation procedure (see methods) and in a sense gives the most accurate prediction of how good a system would do strictly within data set 1. C) Distribution of prediction performance in the two-fold cross-validation procedure with wrapper feature selection on data set 1 resolved by individual recording. The histogram shows how many recordings lead to the performance values marked along the x-axis when being the left out and later classified example in the two-fold crossvalidation. The performance in this context was based on the 10 best feature sets (regardless of size) as found in wrapper feature selection on the *n*−1 data set.

Figure 8A illustrates our observations in the case of three classes (bad, intermediate and good). If we use the single best feature set we observe a classification performance around 60% (barely above chance) on data set 2 (yellow line in Fig. 8A), where the ground truth was assumed to be the manual classification of recording quality by expert 1. This performance is observed for feature sets of 10 or less features and then rapidly declines to markedly below chance levels for systems using more features. It compares to around 75% performance for best and top10 group in cross-validation on data set 1 only (blue and red lines in Fig. 8A). The average performance of using top10 group feature sets to train on set 1 and predict set 2 shows a similar pattern (purple line in Fig. 8A). We then also used a compound classifier based on a majority vote of the classifiers trained based on each of the ‘top10’ feature sets (black line in Fig. 8A). This “voting classifier” performs better than the individual ‘top10’ feature set based classifiers with a marked best performance for 13 used features, above which performance drops.

The reduced problem of only 2 classes of recording quality (with intermediate and bad pooled together) shows a somewhat different picture (Fig. 8B). Here, some of the classifiers based on the best feature sets and top10 feature sets and of size 7 or less achieve more respectable performances of 70 to 80% (yellow, purple and black lines in Fig. 8B) and rapidly decline in performance for larger feature sets. The compound voting classifier here performs best for between 6 and 8 features (black line in Fig. 8B) and with around 80% well above chance levels of 59%. This seems to indicate that the simpler 2-class problem is more robustly solved with less features while the more complex 3-class system needs more features to distinguish all 3 classes.

In our testing across data sets we have made two separate advances over the crossvalidation trials with the wrapper feature selection reported above. We have used a true test set that was not seen by the machine learning system until after feature selection and building a classifier with the preferred feature choices has been completed. We have also used data from one expert to predict the data quality of recordings of a different expert. Overall, we see a reduction in prediction accuracy but it is not clear which of the two changes is mainly to blame. To unravel this potential confound, we devised a two-fold crossvalidation procedure that can be run within the data set of a single expert but avoids the potential over-fitting when using a wrapper feature selection approach. In the two-fold crossvalidation procedure, one recording is left out from the data set and then the full wrapper feature selection using crossvalidation (involving more leave-out recordings) is performed on the remaining “*n−*1” recordings. We then choose features and train a classifier on the *n−*1 recordings to eventually predict the quality of the originally left out recording (see Methods for more details). We observe that the performance of voting classifiers based on ‘top10’ feature groups is competitive (Fig. 8A, orange line). In particular, for very small feature sets of 2 or 3 features, we observe above 73% correct results. When compared to the 76–77% maximal success rate in the standard wrapper method (red and blue lines in Fig. 8A), this suggests an effect of over-fitting. However, when compared to the about 60% performance seen in classification of data set 2 based on data set 1 (yellow, purple, black lines in Fig. 8A), a similarly strong, if not stronger, effect of predicting across experts becomes apparent.

From this numerical experiment with twofold leave-one-out crossvalidation we conclude that in the three class problem, we can reasonably expect a 73% performance of fully correct predictions when remaining within the recordings of a single expert.

The observed performances imply that misclassification occurs in a few cases. It is interesting to ask whether these cases are due to the classifiers being unreliable, i.e. misclassifying a given recording occasionally but getting it right as well, or whether the failures are due to particular recordings (for example the specific examples discussed above in relationship with classifier 6–2133 in Figure 6) that are consistently classified incorrectly. To address this question more systematically we calculated for each individual recording how often it was classified correctly in the 2-fold wrapper classification procedure. The results are shown in Figure 8C. The histogram indicates clearly that there is a large majority of recordings that are either always predicted correctly (right hand side bar) or always predicted incorrectly (left hand side bar), whereas there are only a few recordings where the repeated use of the classifier method yields different results from trial to trial (bars in the middle).

Another aspect of judging the performance of the automated system is how its consistency of reproducing expert judgement compares to the consistency of expert judgement between individual experimenters. To obtain some insight into this problem we compared the opinions of two experts against each other and against one of the best machine classifiers. The results are shown in tables 2 (data set 1) and 3 (data set 2). We observe that on both data sets, the consistency among human experts and between humans and machine are comparable. On data set 2 the machine classifier even seems to be more consistent with expert 1(its trainer) than is expert 2 with expert 1. When mixing training and predictions from different data sets, however, the performance drops measurably (last column in table 3).

**Table 2:** Human and machine judgements on data set 1.^a^

	Classification expert 1	Classification expert 2	Classification 6/2133	Two-fold crossvalidation top10 voting
Classification expert 1	1	0.70	0.73	0.69
Classification expert 2	0.70	1	0.68	0.65
Classification 6/2133	0.73	0.68	1	0.80
Two-fold crossvalidationtop10 voting	0.69	0.65	0.80	1

a Correlation between the prediction vectors (bad = −1, intermediate = 0, good = 1).

**Table 3:** Human and machine judgements on data set 2.^a^

	Classification expert 1	Classification expert 2	Classification 8/141	Classification top10 voting across^b^
Classification expert 1	1	0.70	0.85	0.73
Classification expert 2	0.70	1	0.68	0.53
Classification 8/141	0.85	0.68	1	0.69
Classification top10 voting across^b^	0.73	0.53	0.69	1

a Correlation between the prediction vectors (bad = −1, intermediate = 0, good = 1).

b The last column and row show the correlation to the result of feature selection and training on data set 1 and then predicting data set 2 with all members of the top10 group of size 13 (the one performing best, see Figure 8).

## Conclusions

We have presented a first attempt at using machine learning methods for automatically judging the quality of electrophysiological recordings. The proposed system is fully automated from data pre-processing, feature extraction, feature selection, all the way to a final classification decision so that, even though the employed wrapper approach needs considerable computation time, there is no burden on the time of the researcher for using a system like this.

While full automation suggests a degree of objectivity it is worth keeping in mind that the human judgment on the training examples plays a decisive role in the performance of the system. Nevertheless, once features have been collected and a classifier has been trained, the procedure is fully transparent and reproducible. Authors using the method would only need to publish the feature choices and support vectors of their classifier as supplementary material to their publication to fully document the process of choosing appropriate recordings.

We observed that the automatic system performs as consistently compared to its trainer human expert as another human expert. The success rates for reproducing the “ground truth” human judgement were on the order of almost 80% for the three class problem of distinguishing “bad”, “intermediate” and “good” recordings and more than 90% for the reduced two class problem of only distinguishing “good” versus “not good”. These success rates appear high enough to make the system useful for applications with high data throughput.

## Supporting Information

Data S1 **PDF collection of example plots of the data used in our study.** The data is displayed in its original unprocessed form and each plot is labelled with the corresponding file name of the original data files, which are included in Toolbox S1.
(PDF)

Toolbox S1 **Matlab toolbox and example original data files of data used in this study.** The installation and use of the Matlab tools is explained in the included README file.
(ZIP)

## References

[1] Asmild M, Oswald N, Krzywkowski KM, Friis S, Jacobsen RB, et al (2003) Upscaling and automation of electrophysiology: toward high throughput screening in ion channel drug discovery. Receptors Channels 9: 49–58. 12825298

[2] Priest BT, Swensen AM, McManus OB (2007) Automated electrophysiology in drug discovery. Curr Pharm Des 13: 2325–2337. 17692004 10.2174/138161207781368701

[3] Mathes C (2006) QPatch: the past, present and future of automated patch clamp. Expert Opin Ther Targets 10: 319–327. 16548779 10.1517/14728222.10.2.319

[4] Panzeri S, Senatore R, Montemurro MA, Petersen RS (2007) Correcting for the sampling bias problem in spike train information measures. J Neurophysiol 98: 1064–1072. 17615128 10.1152/jn.00559.2007

[5] Koolen N, GGligorijevich I, Van Huffel S (2012) Reliability of statistical features describing neural spike trains in the presence of classification errors. International Conference on bio-inspired systems and signal processing (BIOSIGNALS 2012), Vilamoura, Portugal. 169–173.

[6] Wiltschko AB, Gage GJ, Berke JD (2008) Wavelet filtering before spike detection preserves waveform shape and enhances single-unit discrimination. J Neurosci Methods 173: 34–40. 18597853 10.1016/j.jneumeth.2008.05.016 PMC2602872

[7] Wilson SB, Emerson R (2002) Spike detection: a review and comparison of algorithms. Clin Neurophysiol 113: 1873–1881. 12464324 10.1016/s1388-2457(02)00297-3

[8] Lewicki MS (1998) A review of methods for spike sorting: the detection and classification of neural action potentials. Network 9: R53–78. 10221571

[9] Franke F, Natora M, Boucsein C, Munk MH, Obermayer K (2010) An online spike detection and spike classification algorithm capable of instantaneous resolution of overlapping spikes. J Comput Neurosci 29: 127–148. 19499318 10.1007/s10827-009-0163-5 PMC2950077

[10] Takahashi S, Anzai Y, Sakurai Y (2003) Automatic sorting for multi-neuronal activity recorded with tetrodes in the presence of overlapping spikes. J Neurophysiol 89: 2245–2258. 12612049 10.1152/jn.00827.2002

[11] Lei H, Reisenman CE, Wilson CH, Gabbur P, Hildebrand JG (2011) Spiking patterns and their functional implications in the antennal lobe of the tobacco hornworm Manduca sexta. PLoS One 6: e23382. 21897842 10.1371/journal.pone.0023382 PMC3163580

[12] Ignell R, Hansson B (2005) Insect Olfactory Neuroethology - An Electrophysiological Perspective. In: Christensen TA, editor. Methods in Insect Sensory Neuroscience. Boca Raton, FL: CRC Press. 319–347.

[13] Matic V, Cherian PJ, Jansen K, Koolen N, Naulaers G, et al.. (2012) Automated EEG inter-burst interval detection in neonates with mild to moderate postasphyxial encephalopathy. 2012 Annual International Conference of the Ieee Engineering in Medicine and Biology Society (Embc): 17–20. 10.1109/EMBC.2012.6345860 23365821

[14] Friedrich R, Ashery U (2010) From spike to graph – a complete automated single-spike analysis. J Neurosci Methods 193: 271–280. 20869399 10.1016/j.jneumeth.2010.09.004

[15] Mohri M, Rostamizadeh A, Talwalkar A (2012) Foundations Of Machine Learning. Cambridge, MA: The MIT Press.

[16] Jackson P (1998) Introduction to expert systems: Addison Wesley.

[17] Saeys Y, Inza I, Larranaga P (2007) A review of feature selection techniques in bioinformatics. Bioinformatics 23: 2507–2517. 17720704 10.1093/bioinformatics/btm344

[18] Nowotny T, Berna AZ, Binions R, Trowell S (2013) Optimal feature selection for classifying a large set of chemicals using metal oxide sensors. Sensors and Actuators B 187: 471–480.

[19] John G, Kohavi R, Pfleger K (1994) Irrelevant features and the subset selection problem. 11th International Conference on Machine Learning. New Brunswick, NJ: Morgan Kaufmann. 121–129.

[20] Kohavi R, John GH (1997) Wrappers for feature subset selection. Artificial Intelligence 97: 273–324.

[21] Olshen L, Breiman JH, Friedman RA, Stone CJ (1984) Classification and Regression Trees. Belmont, CA: Wadsworth International Group.

[22] Weiss SM, Kulikowski CA (1991) Computer Systems that Learn. San Mateo, CA: Morgan Kaufmann.

[23] Galizia CG, Sachse S (2010) Odor coding in insects. In: Menini A, editor. The neurobiology of olfaction. Boca Raton, FL: CRC Press. 35–70. 21882428

[24] Galizia CG, Kimmerle B (2004) Physiological and morphological characterization of honeybee olfactory neurons combining electrophysiology, calcium imaging and confocal microscopy. J Comp Physiol. A 190: 21–38. 10.1007/s00359-003-0469-0 14639486

[25] Chang CC, Lin CJ (2011) LIBSVM: A Library for Support Vector Machines. ACM Transactions on Intelligent Systems and Technology 2.

