# Supplementary figures and images for: Machine Learning for Automatic Prediction of the Quality of Electrophysiological Recordings
Source: PLoS One. 2013 Dec 4;8(12):e80838. doi: 10.1371/journal.pone.0080838 (PMC3851757; doi:10.1371/journal.pone.0080838)

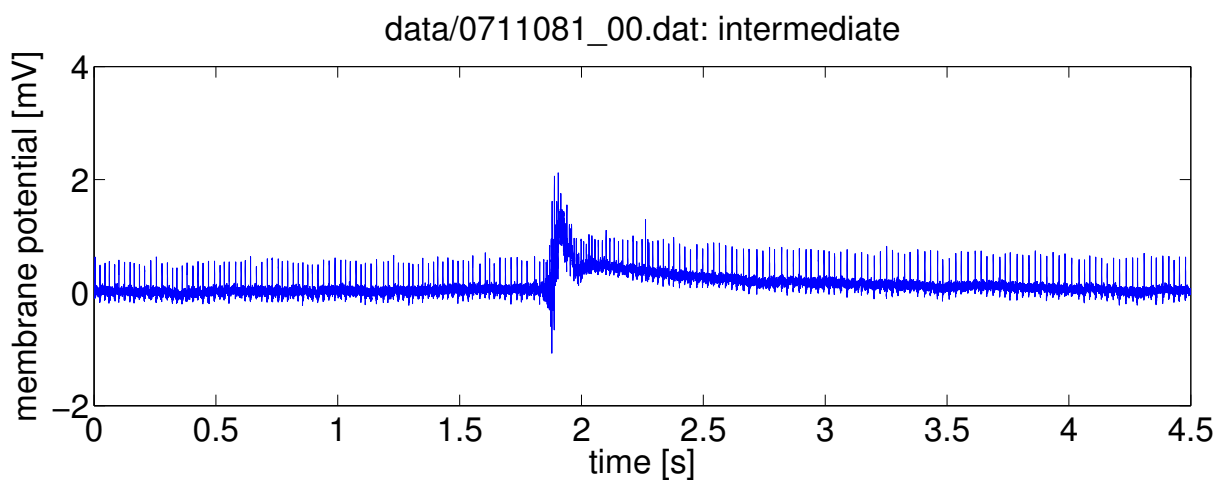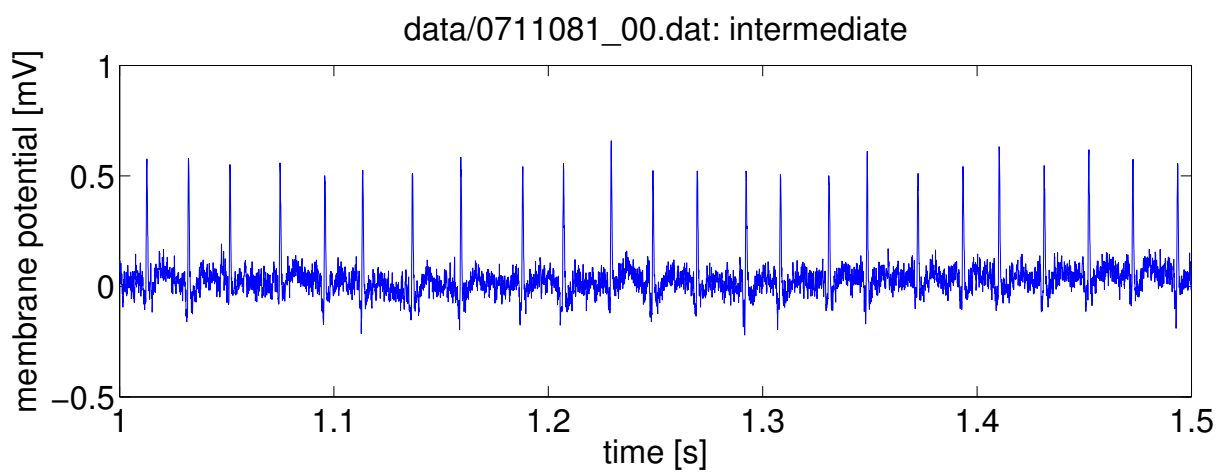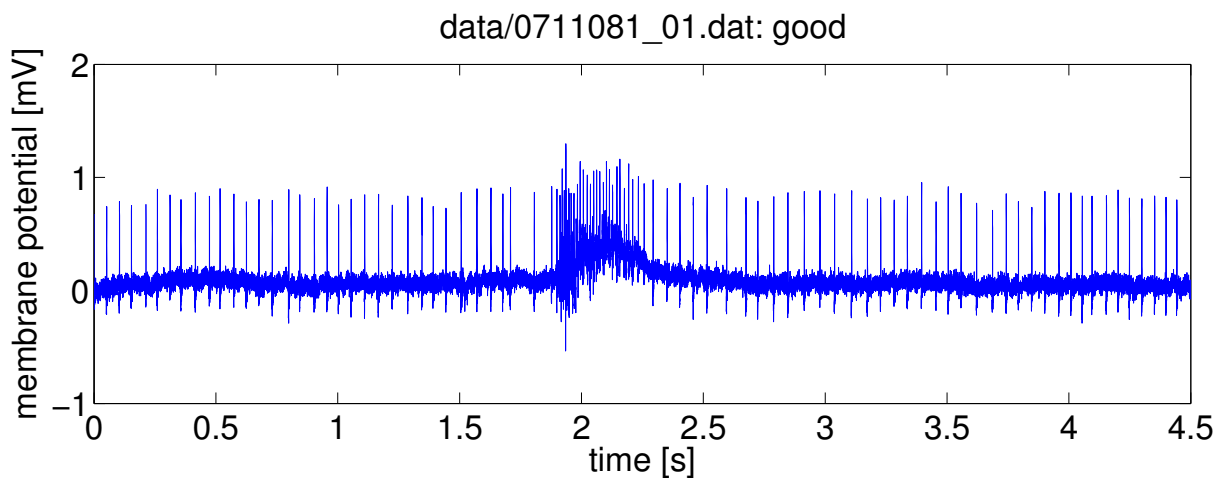

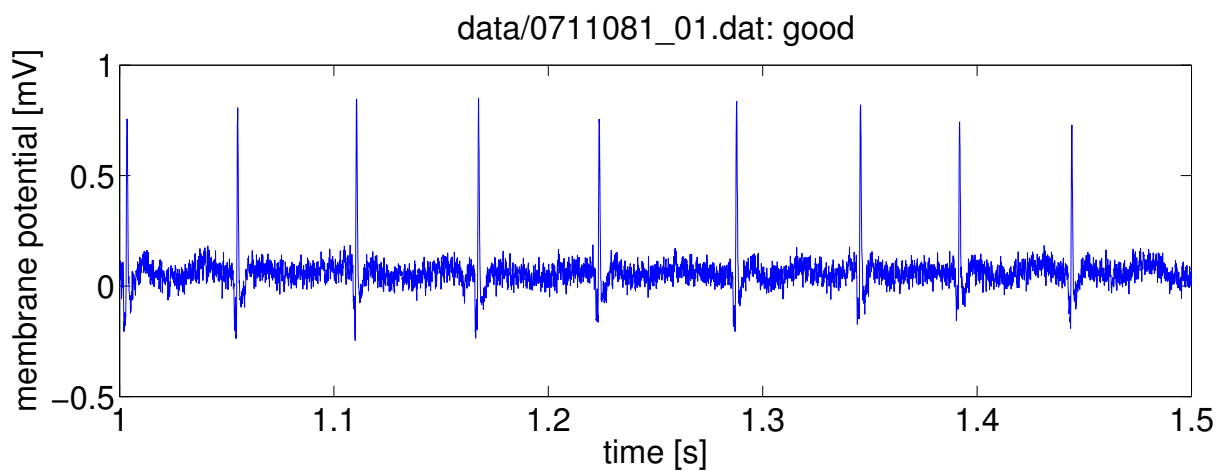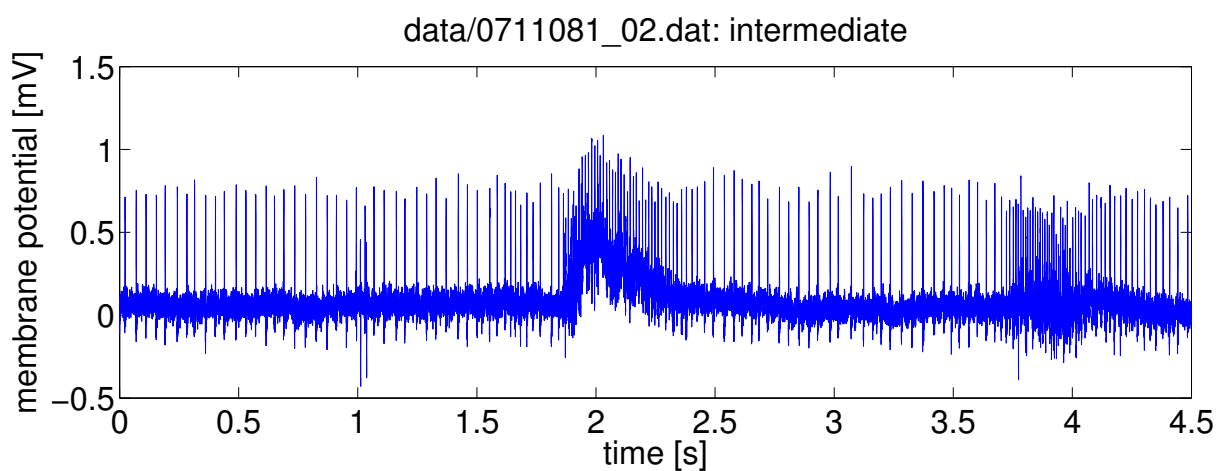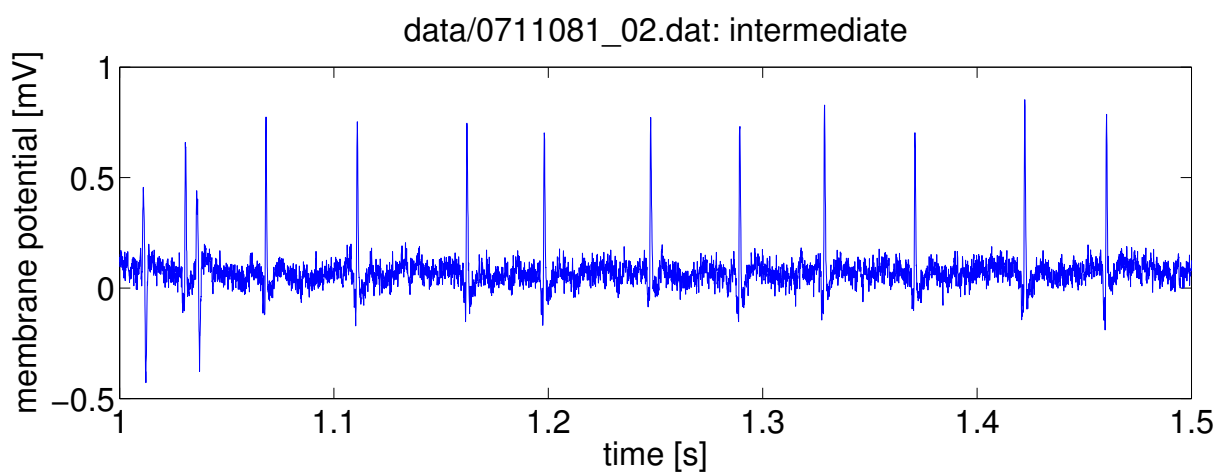

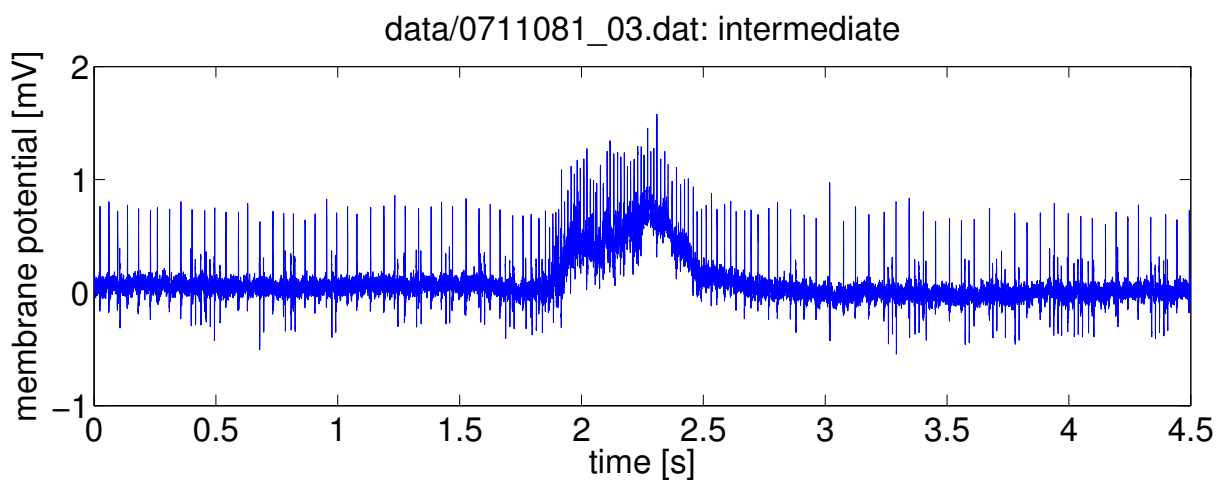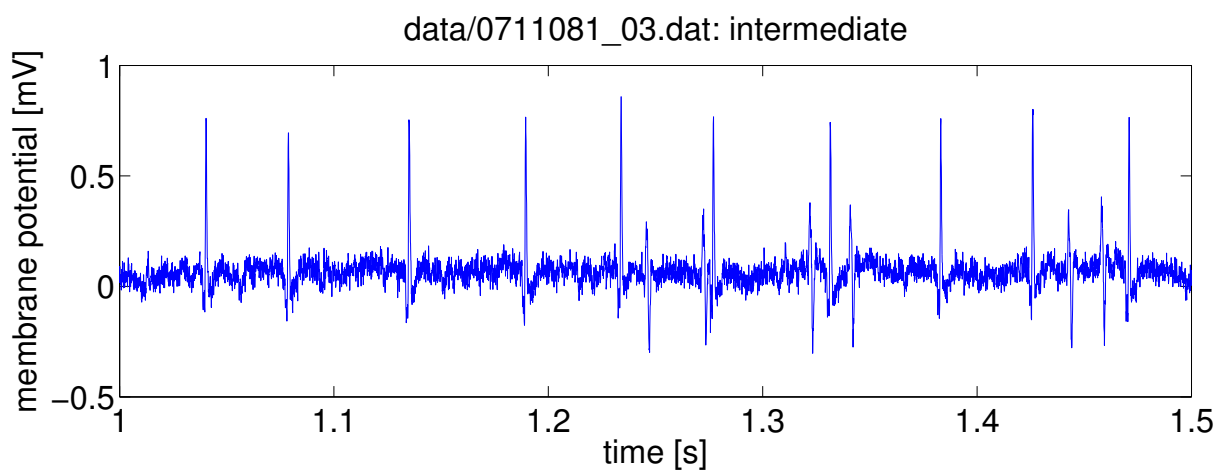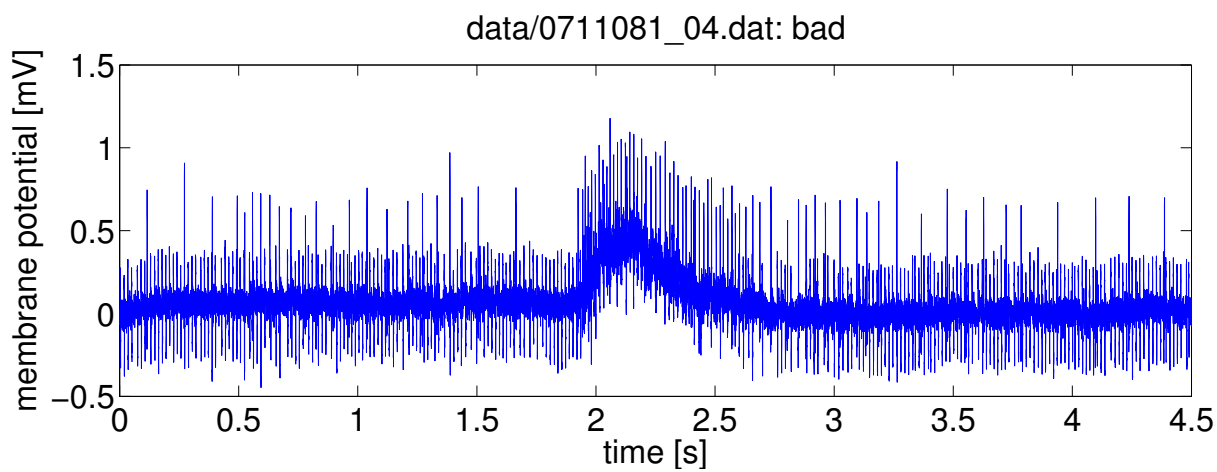

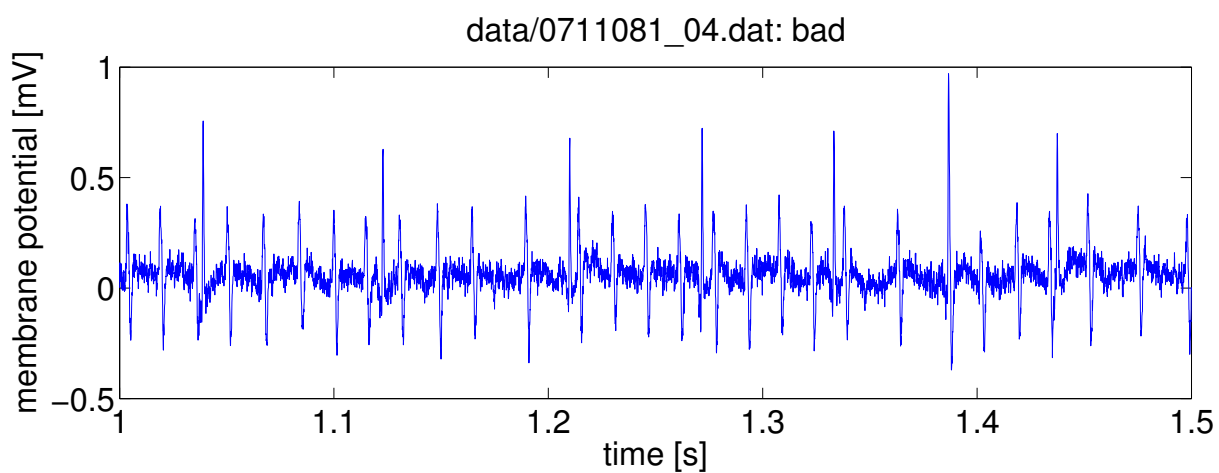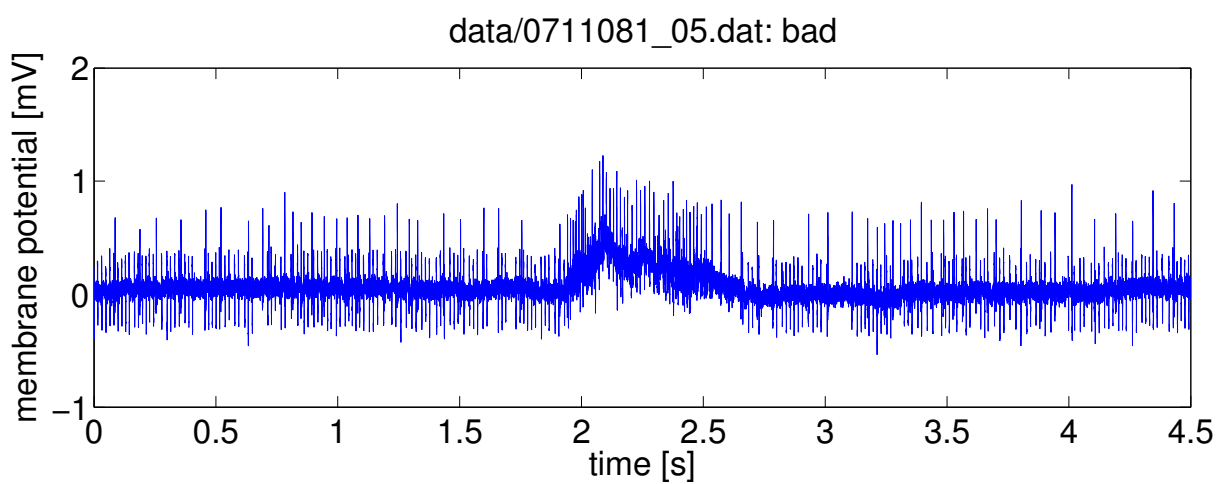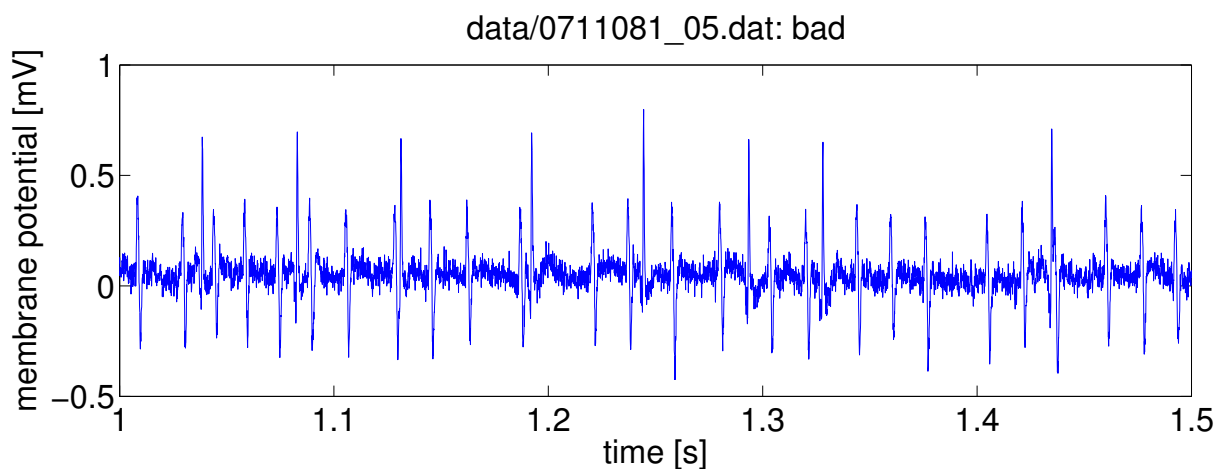

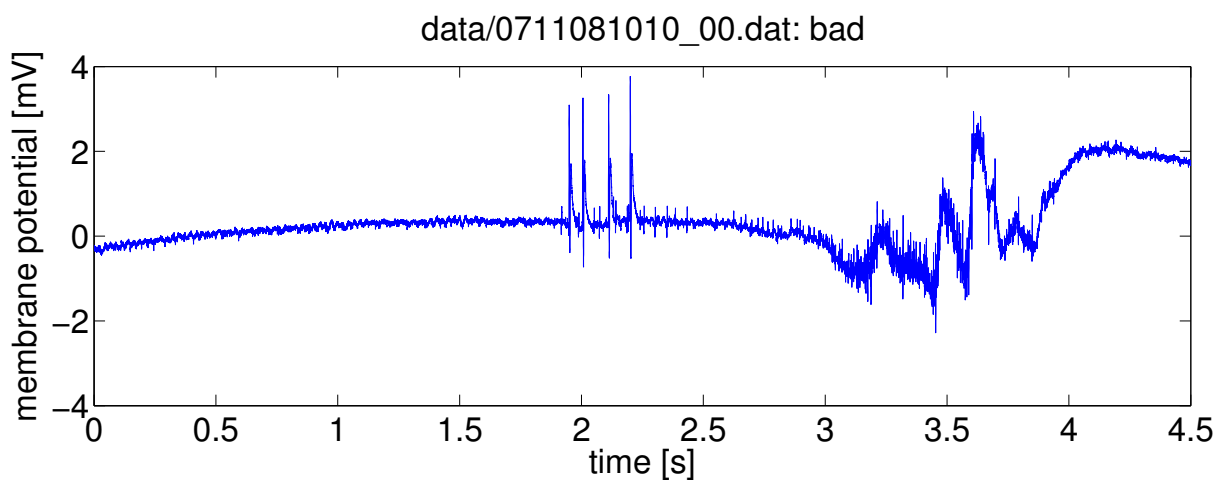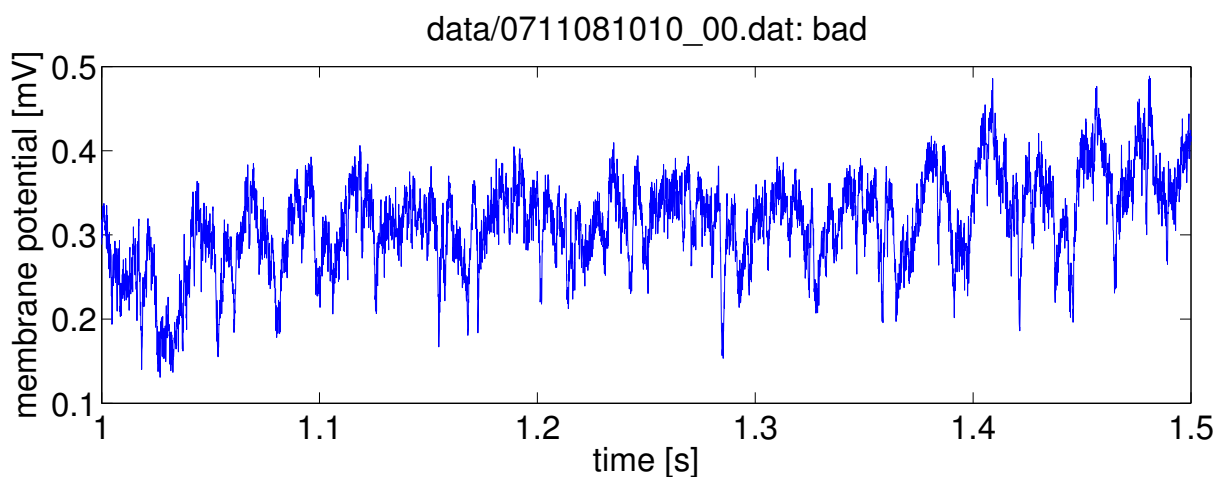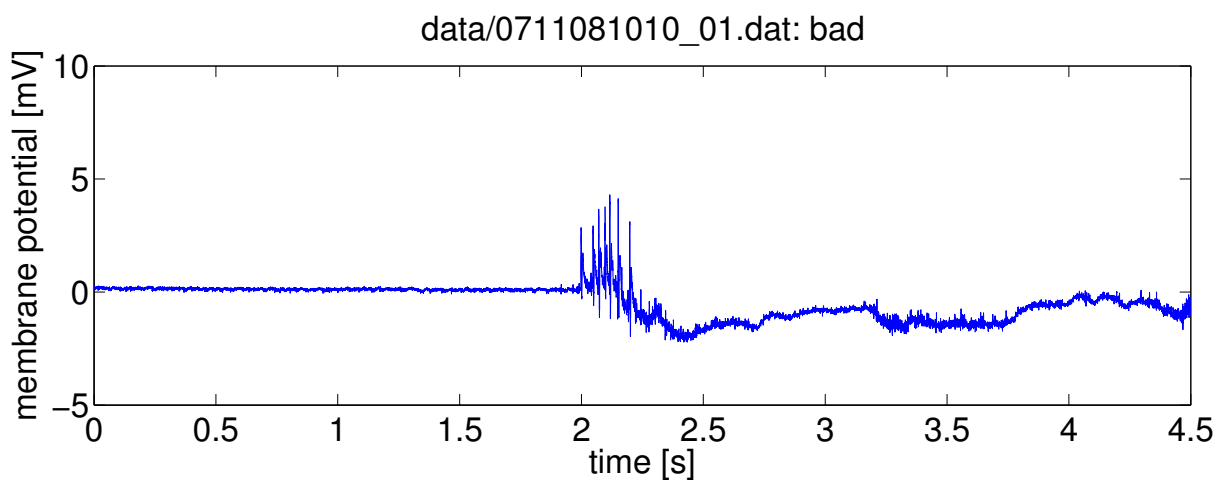

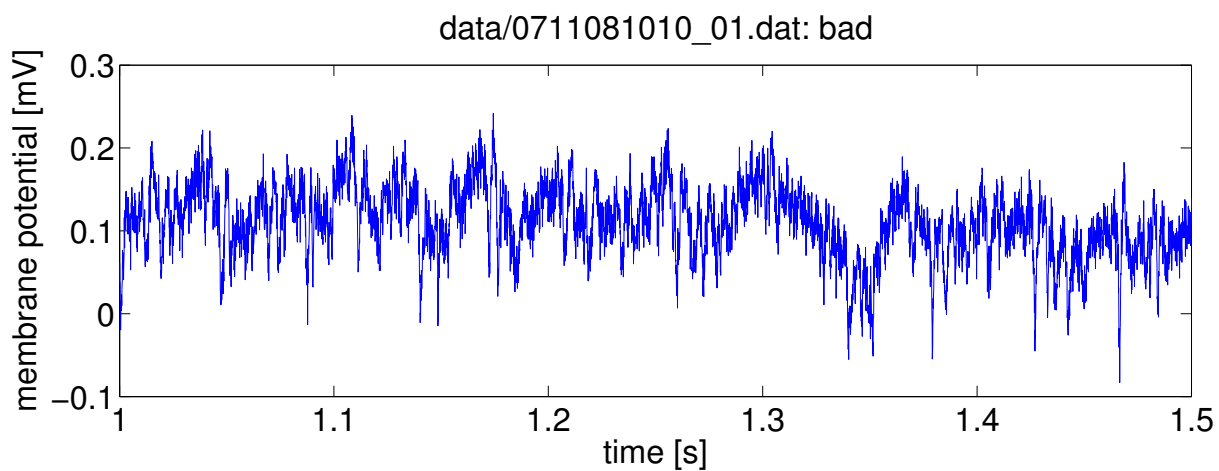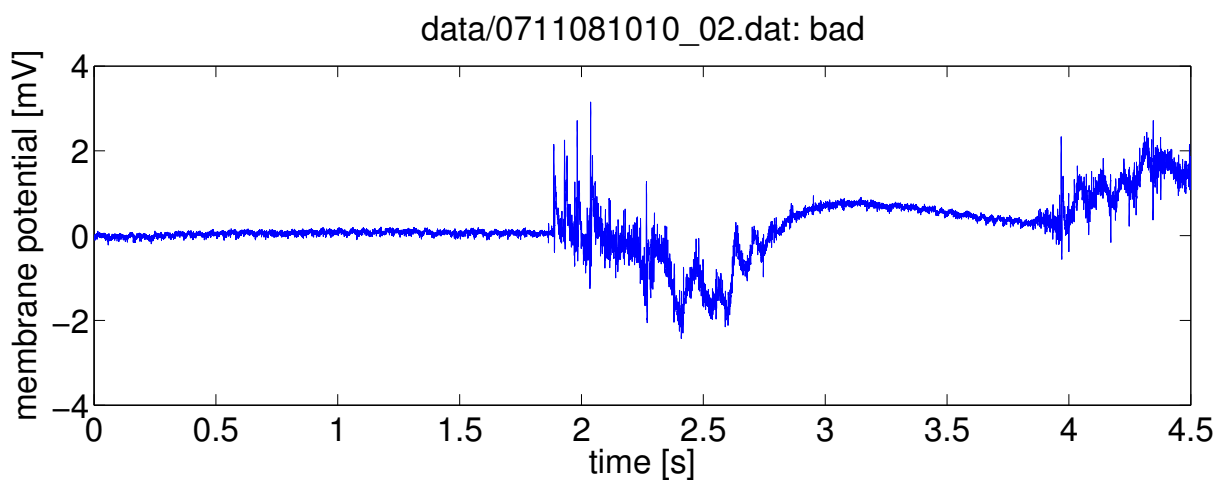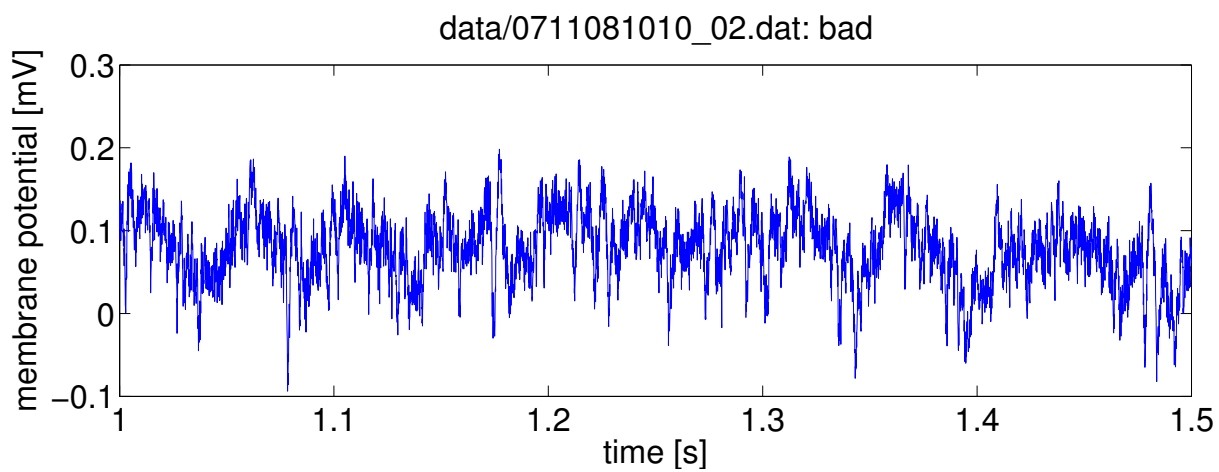

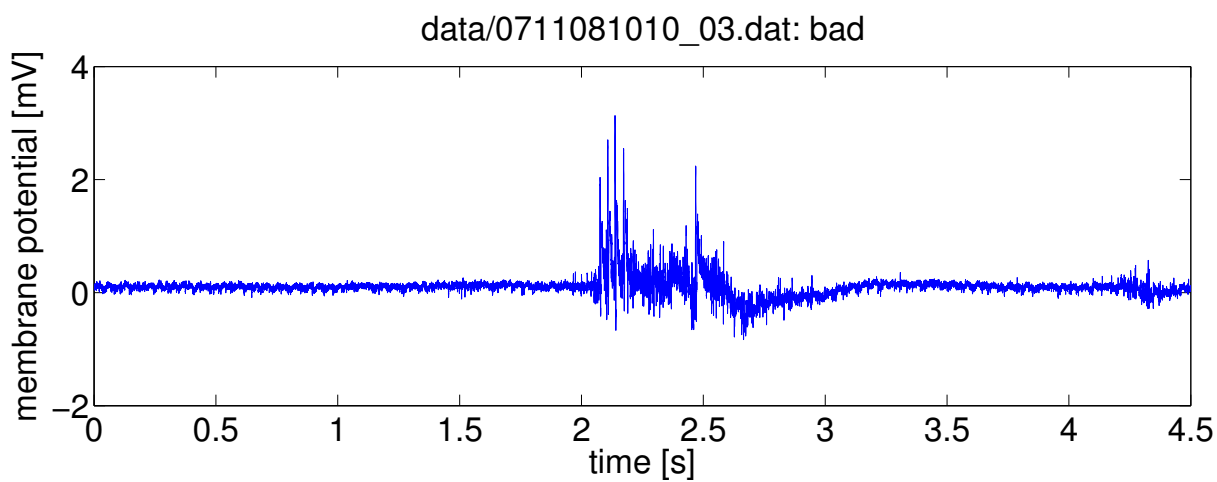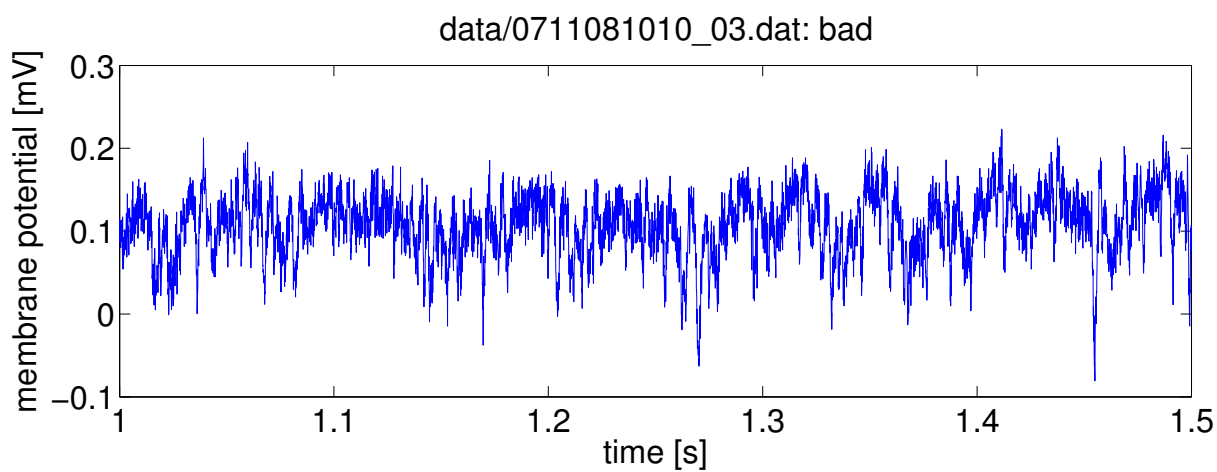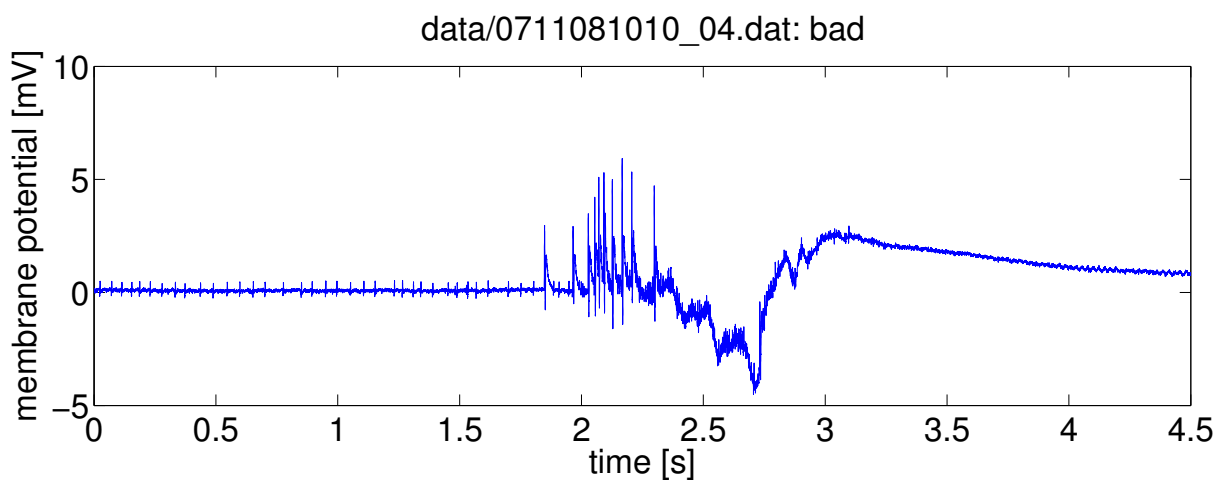

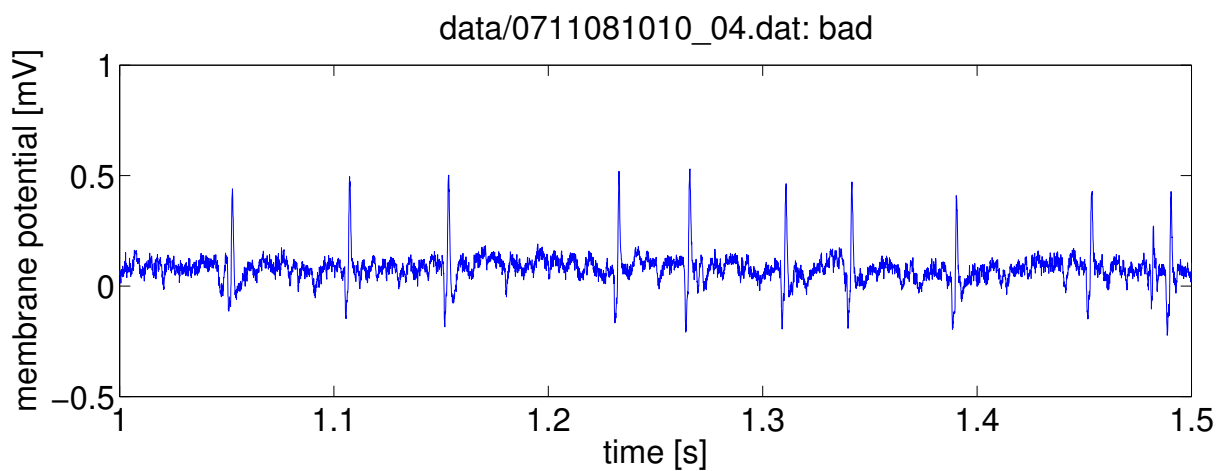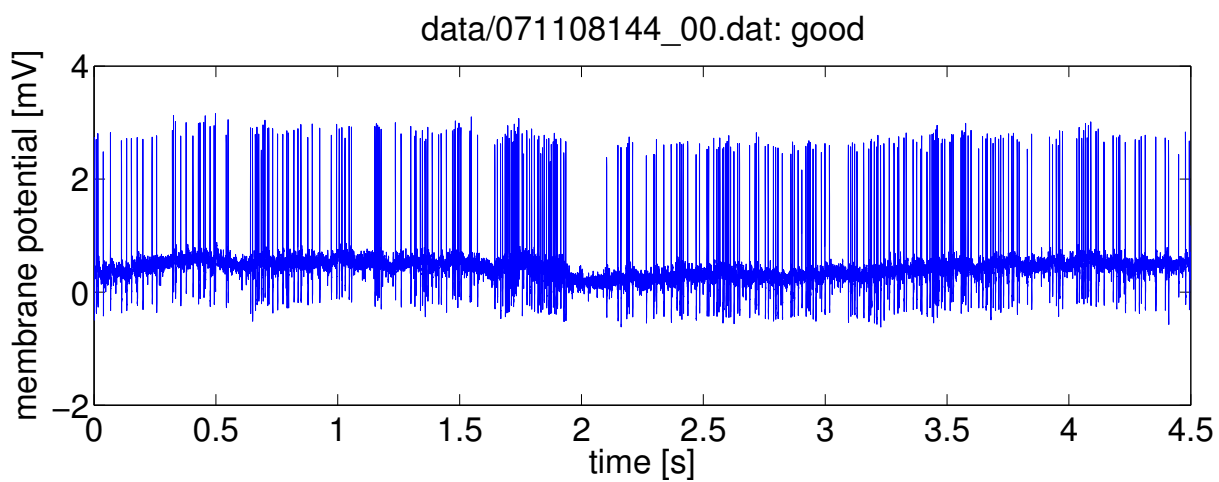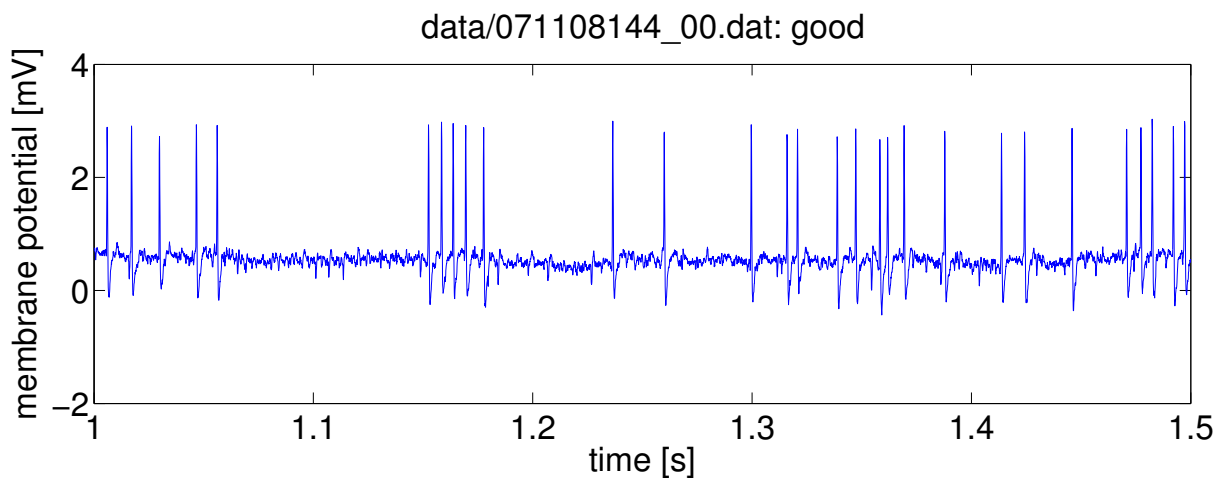

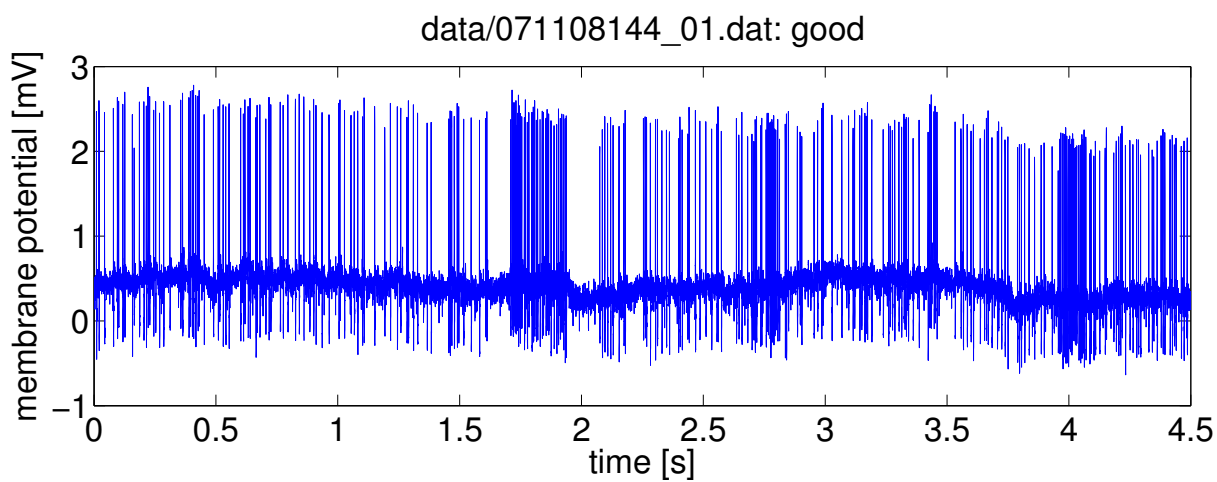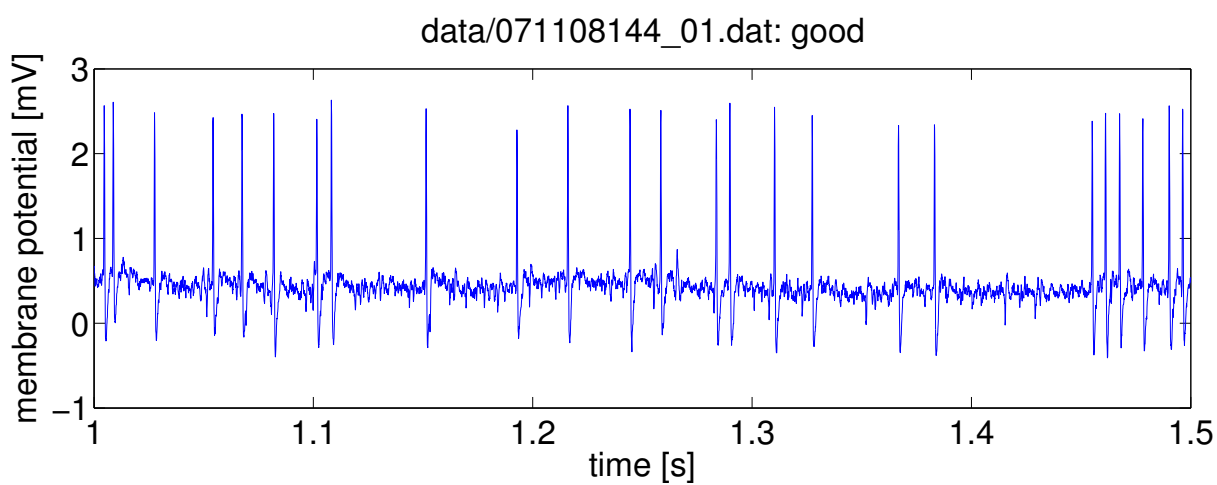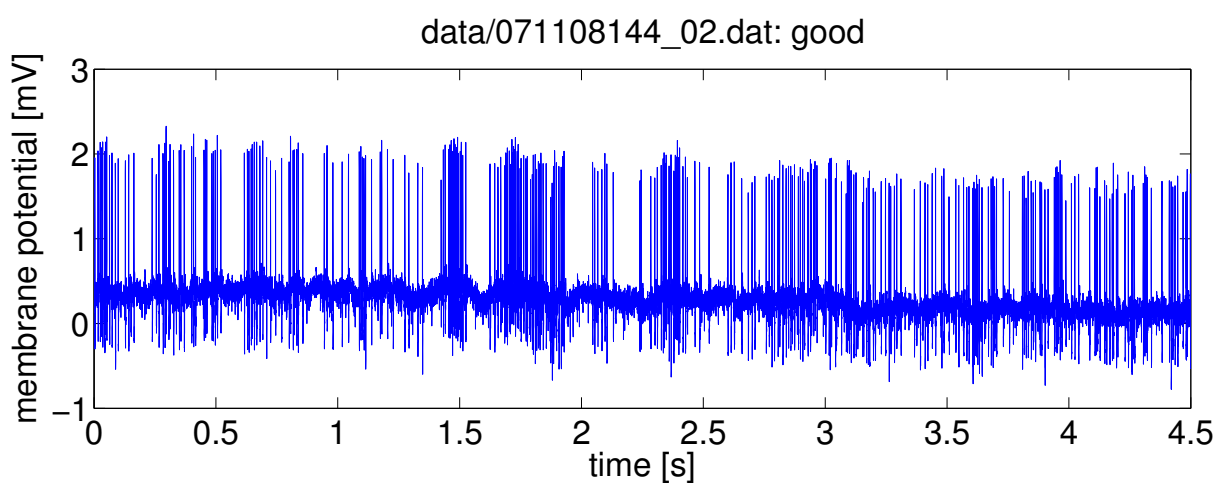

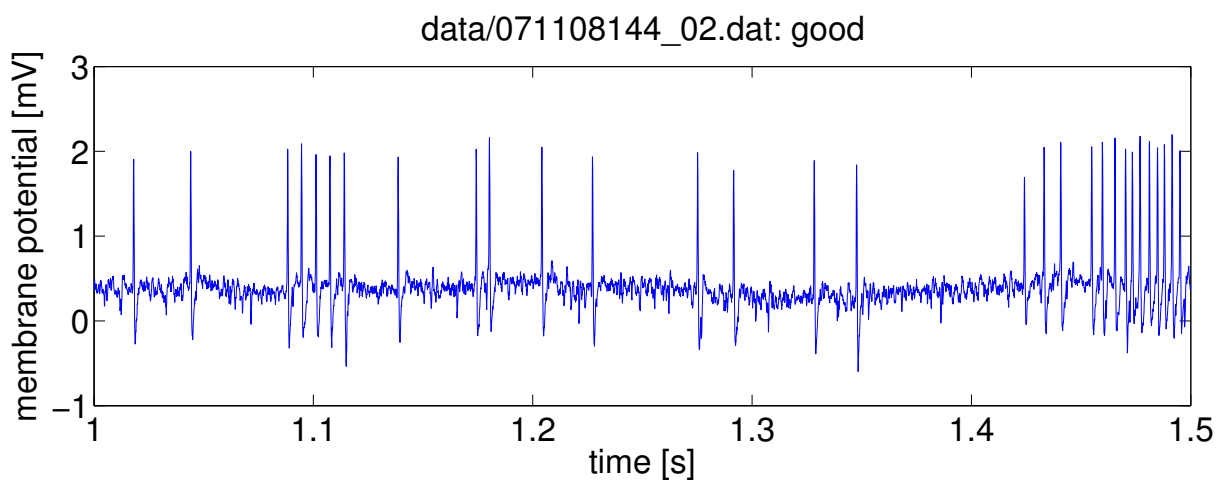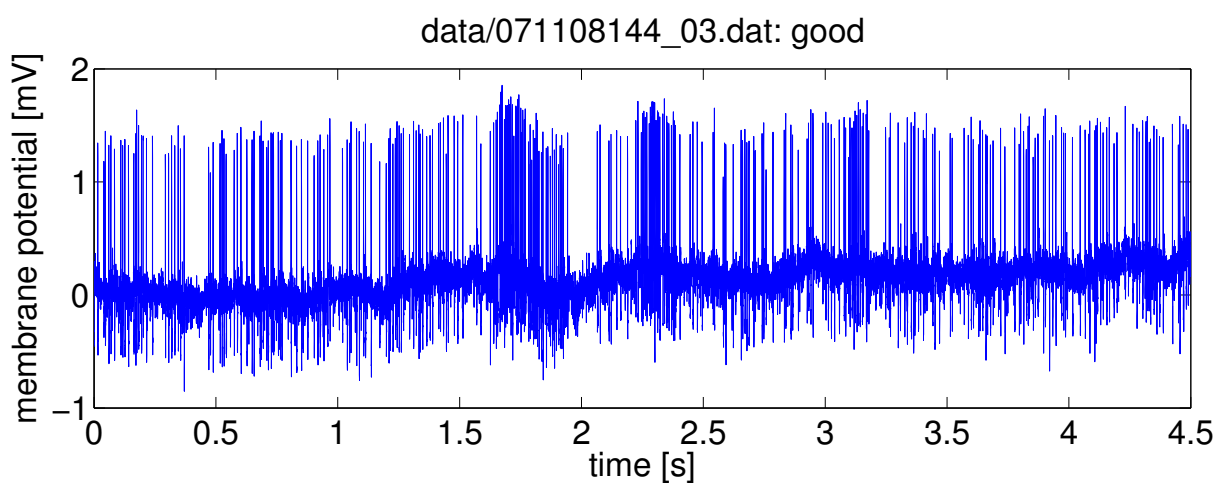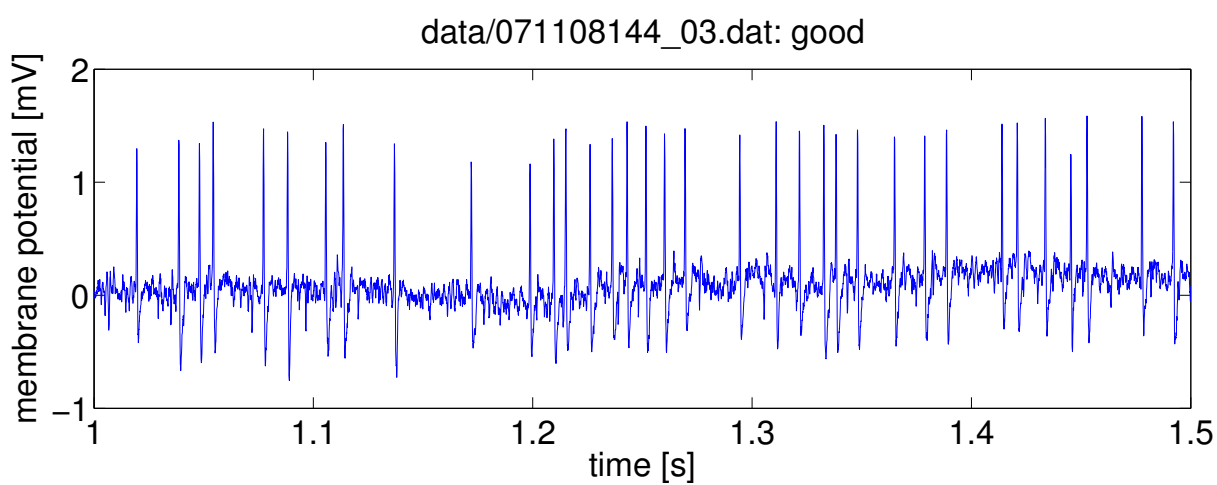

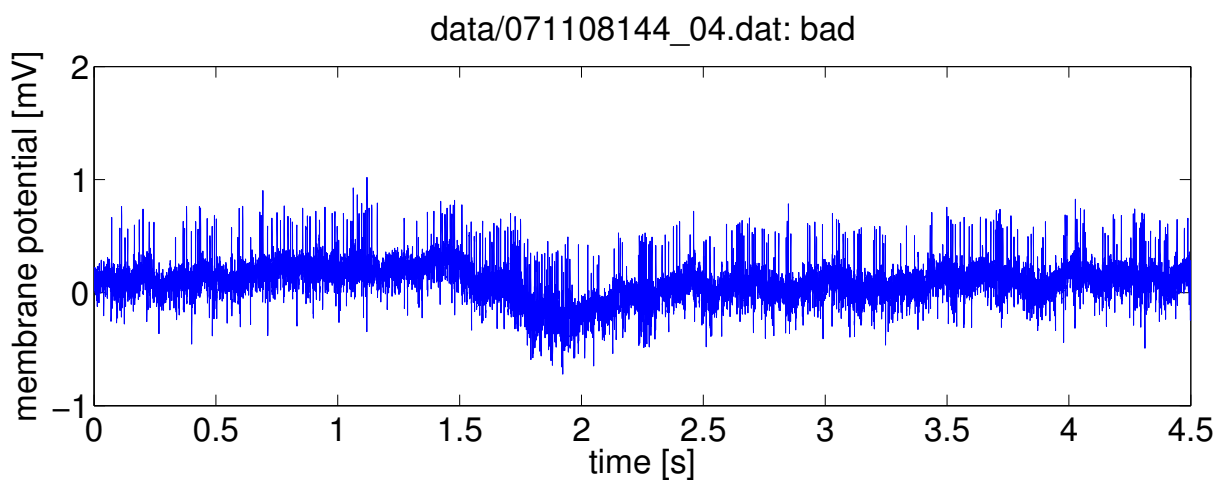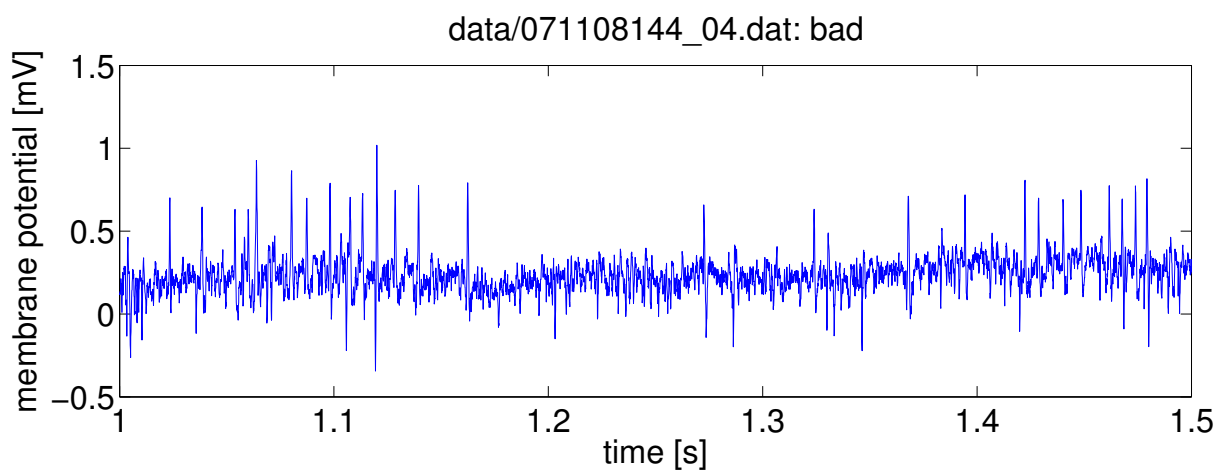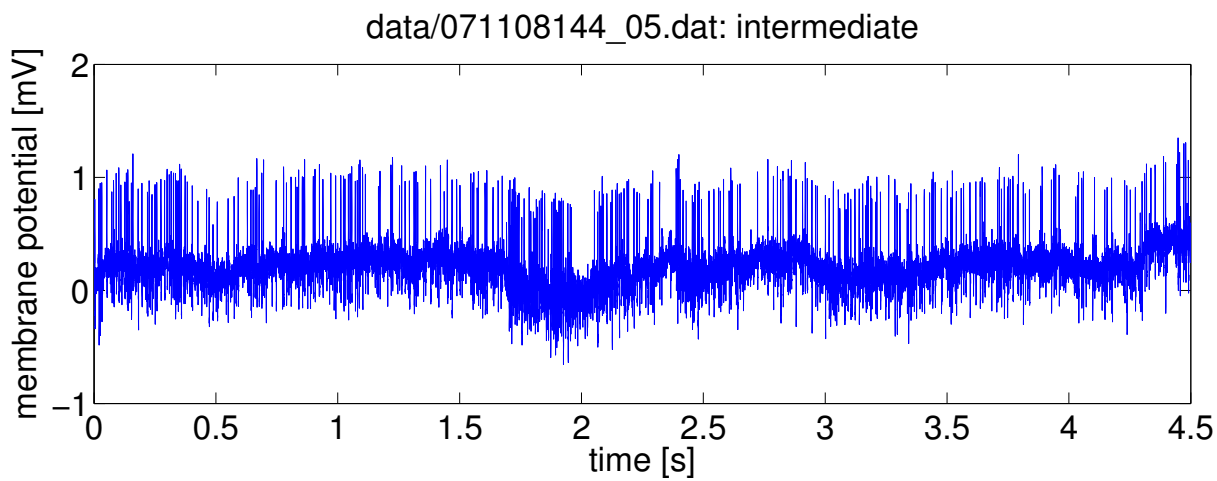

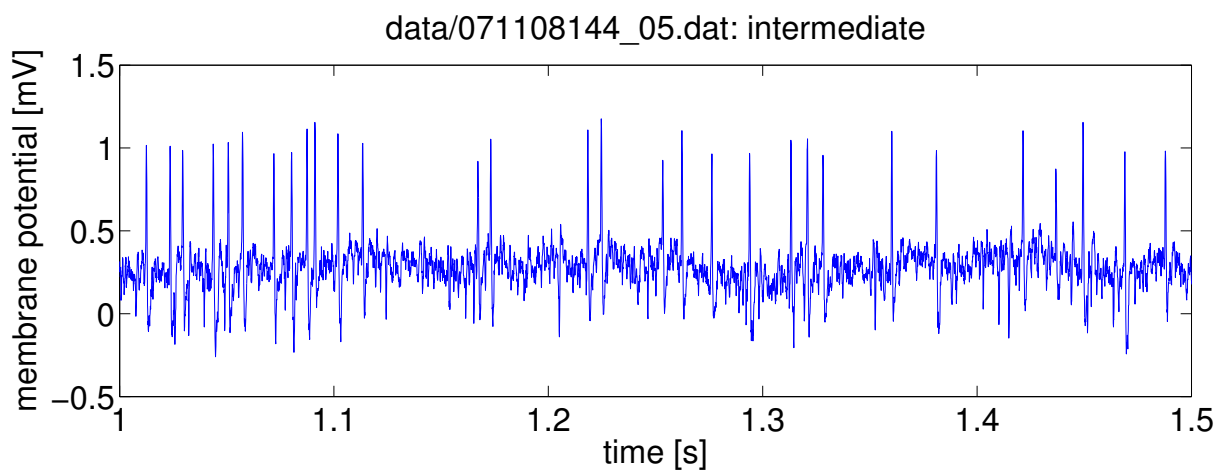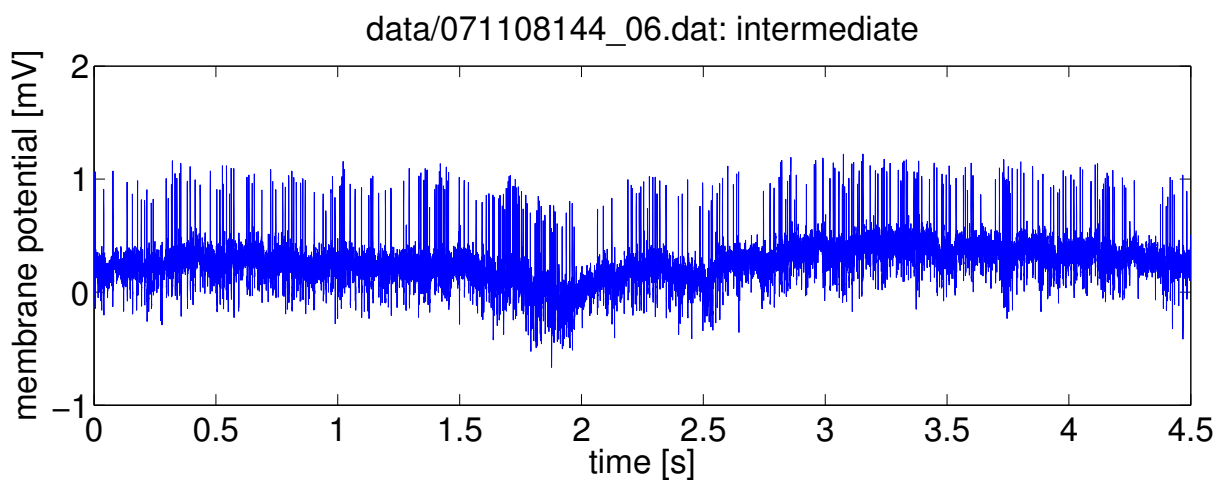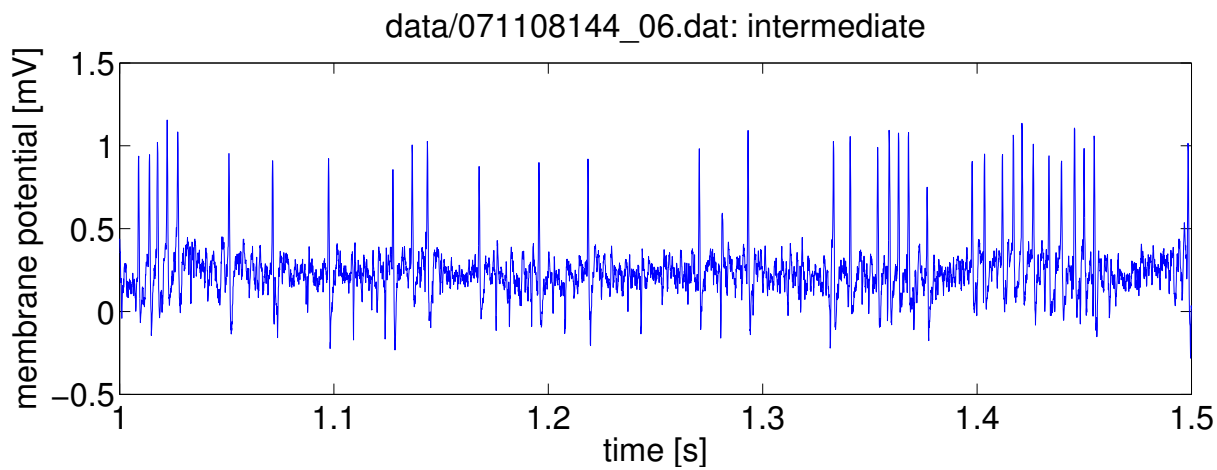

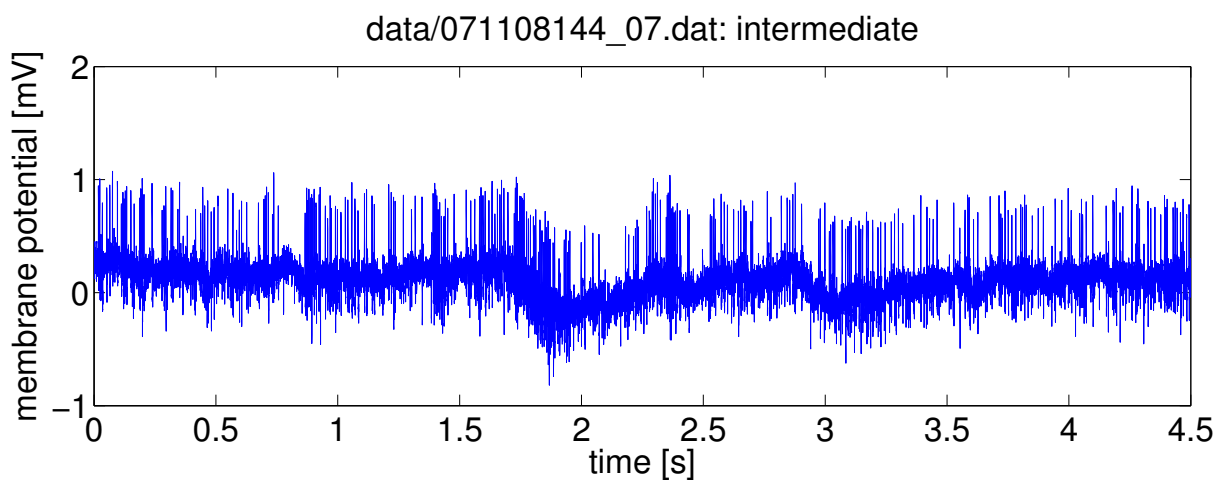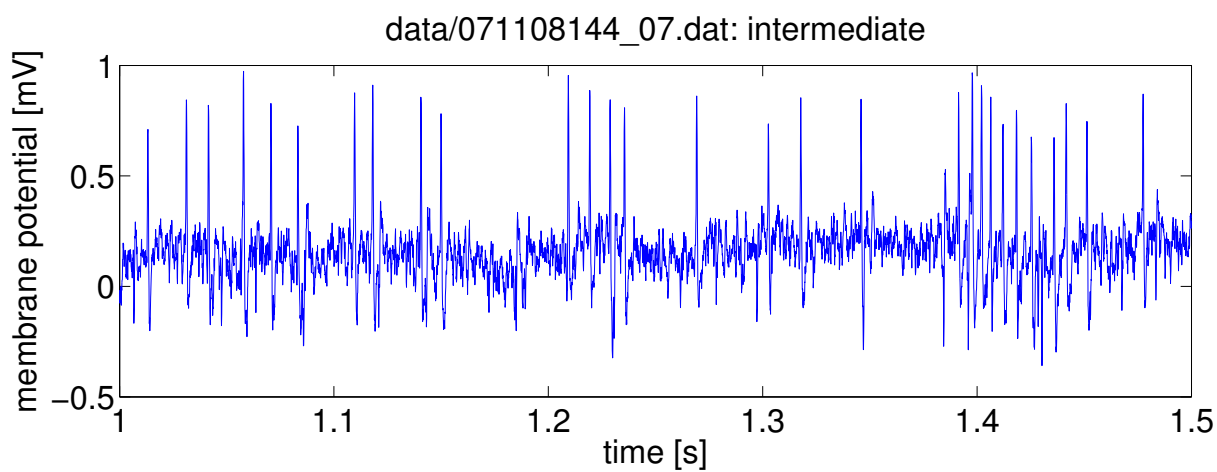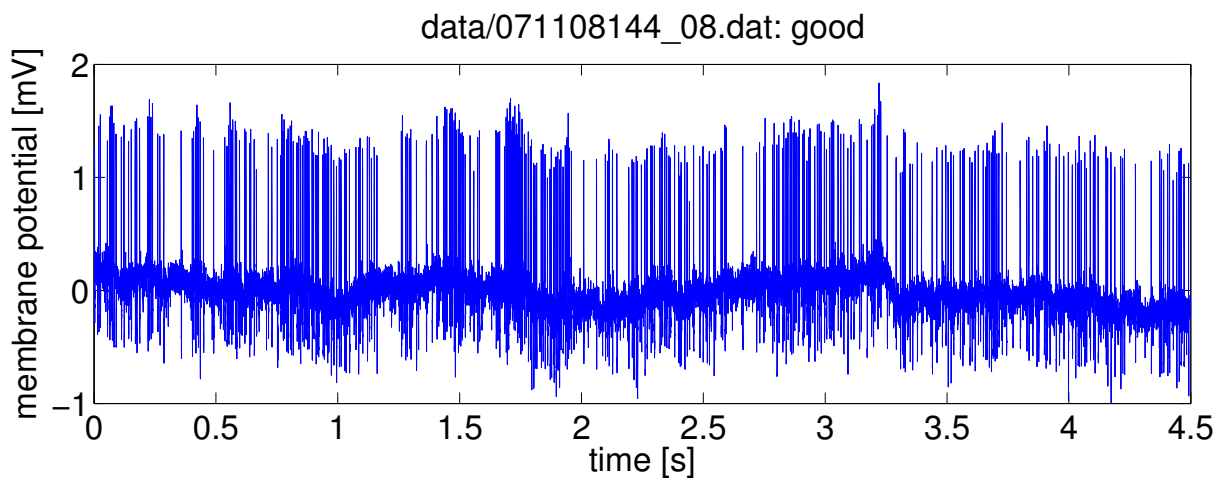

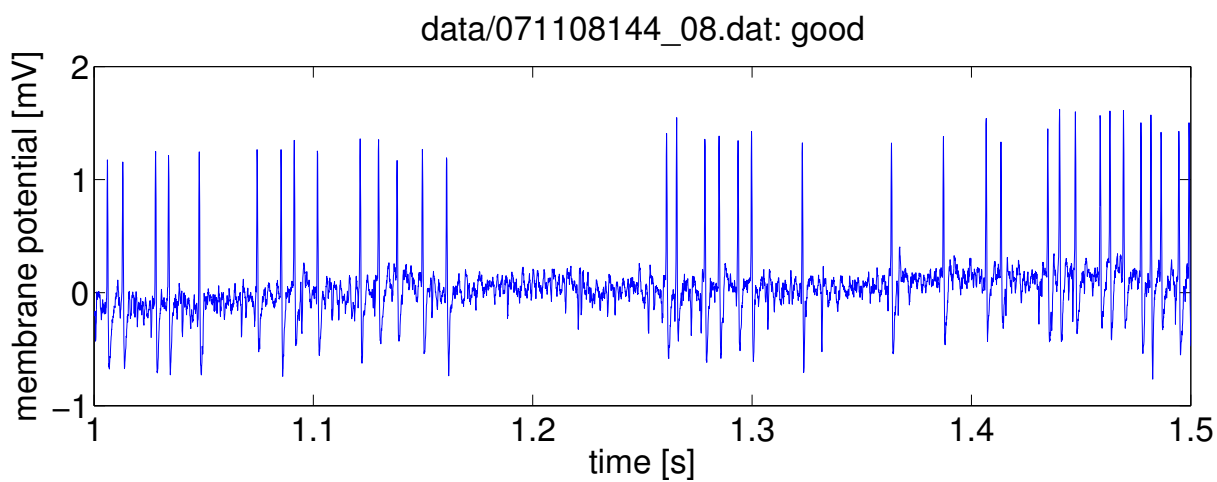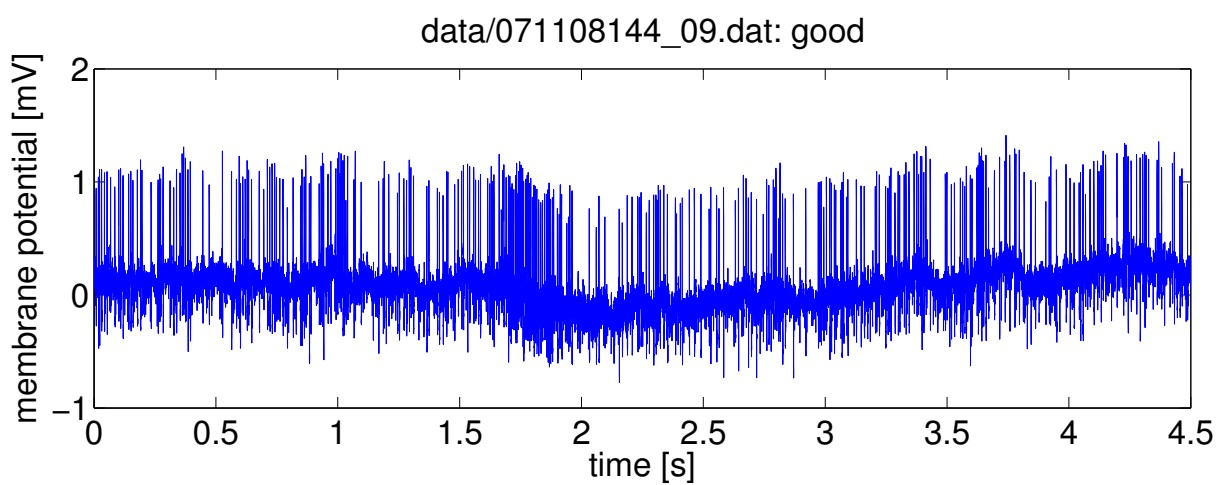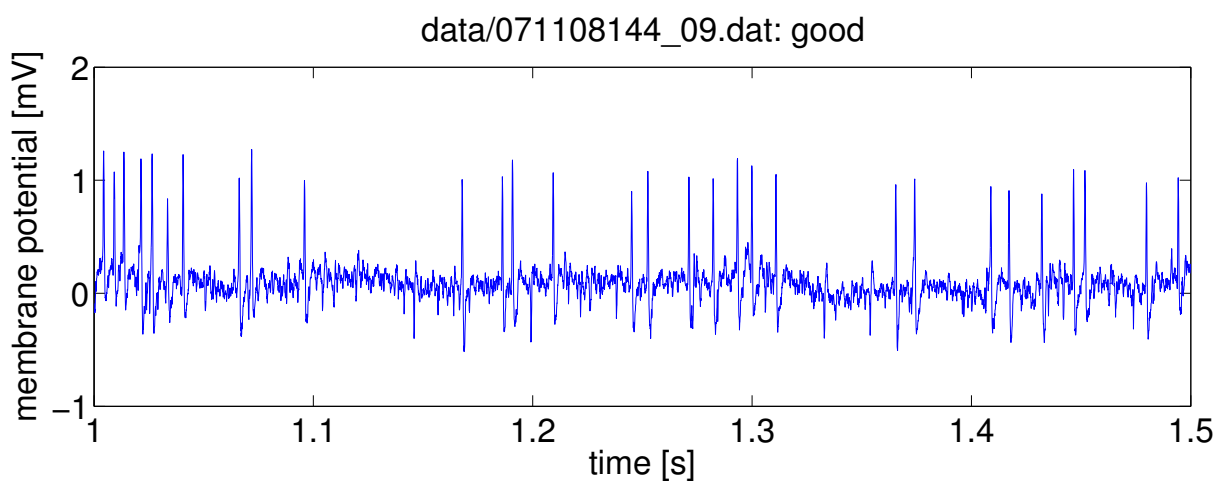

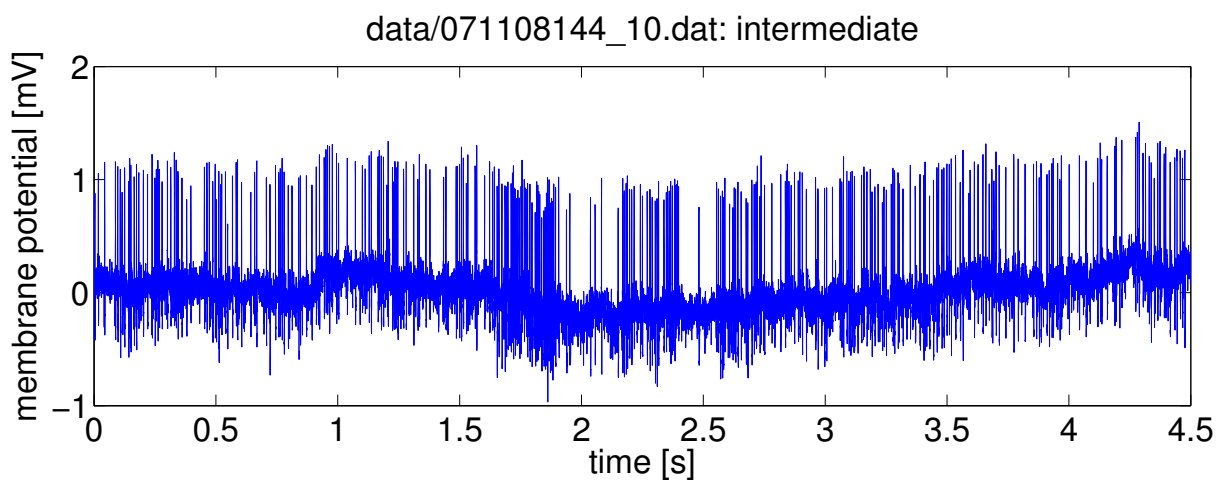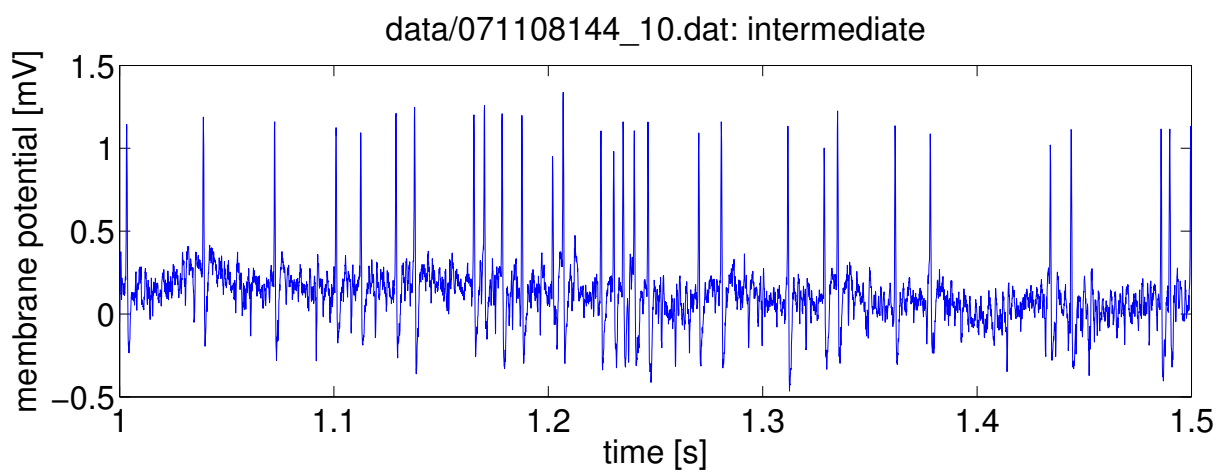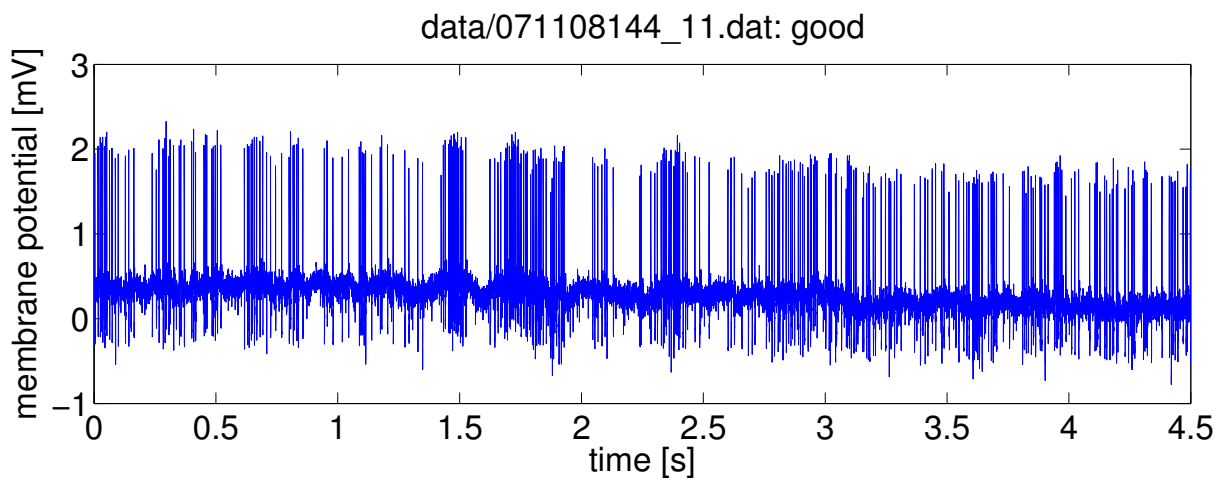

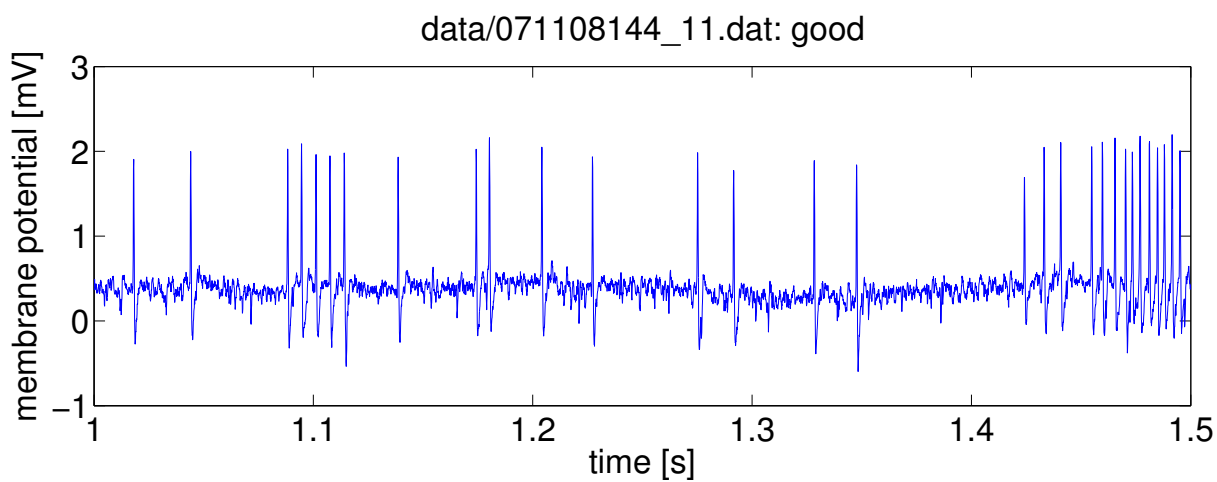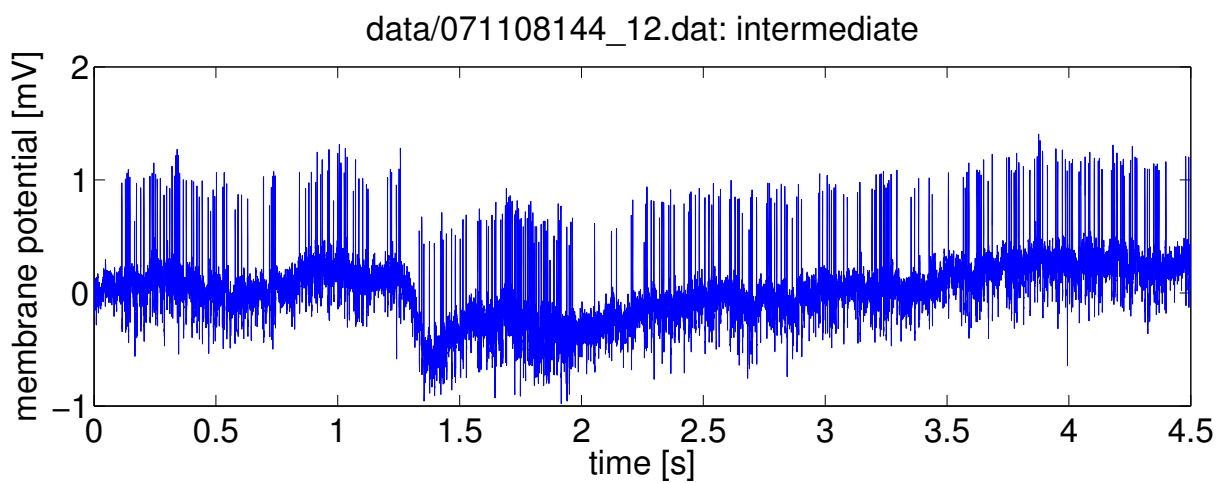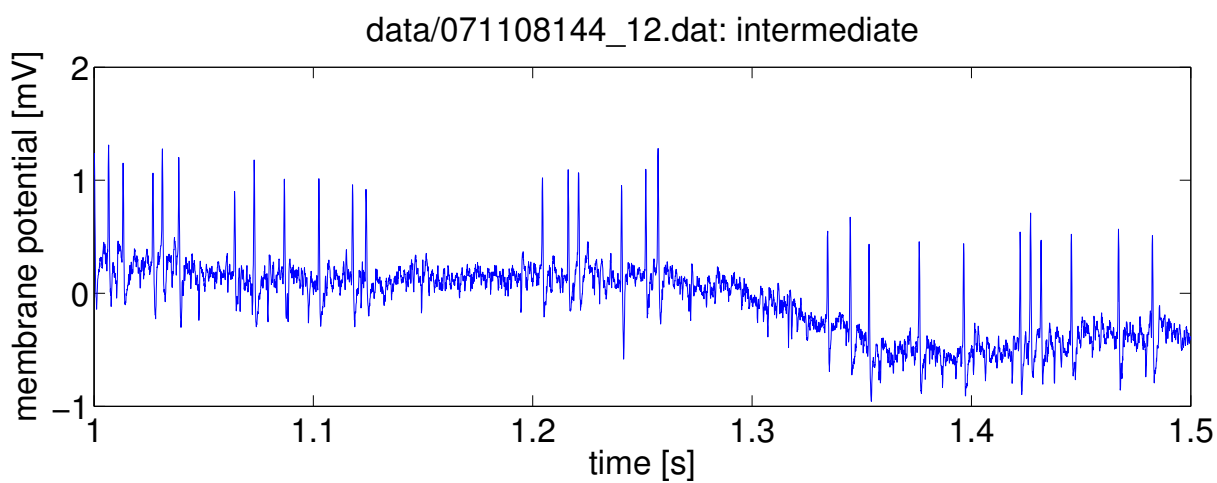

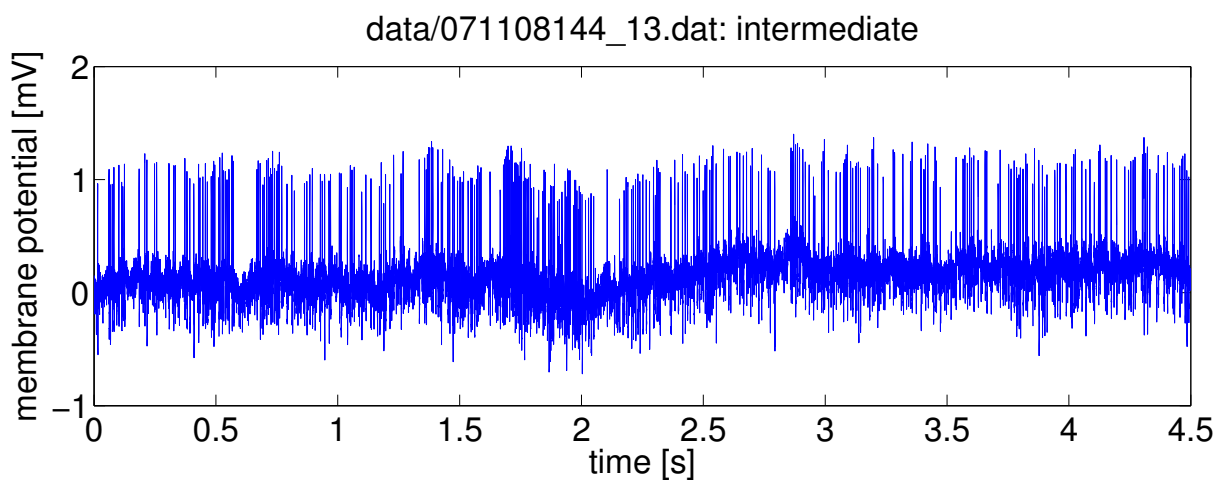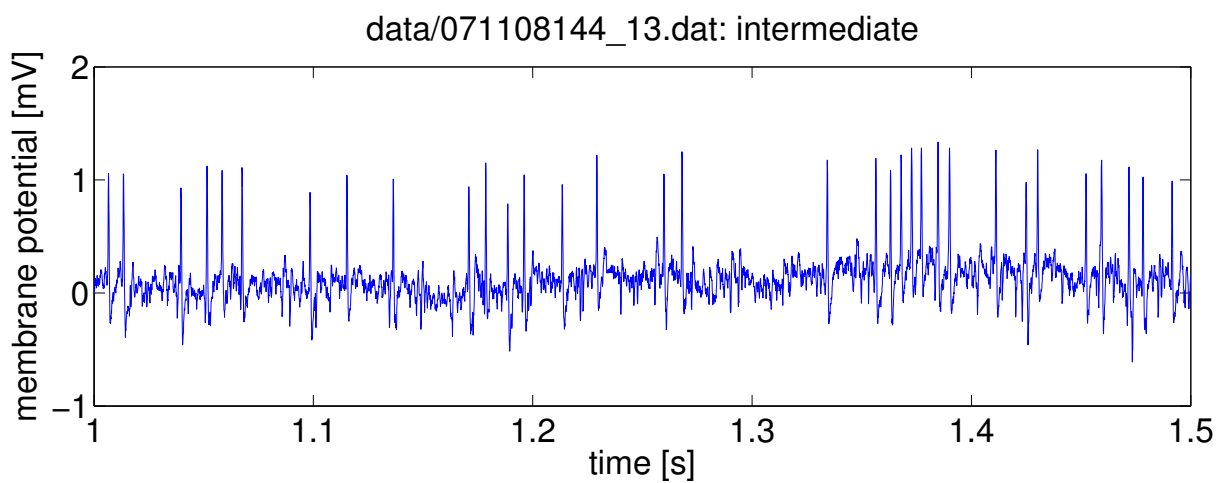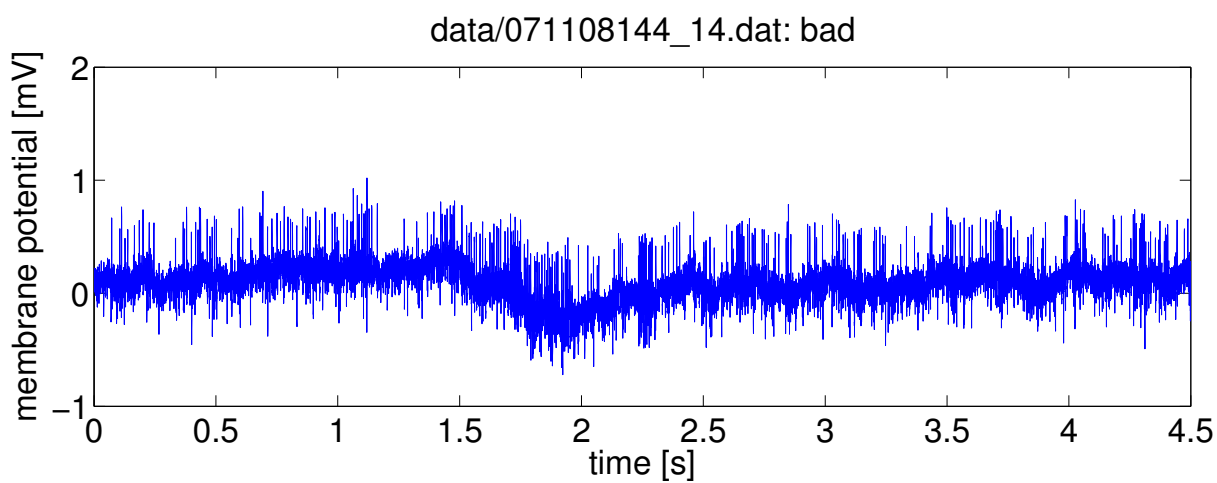

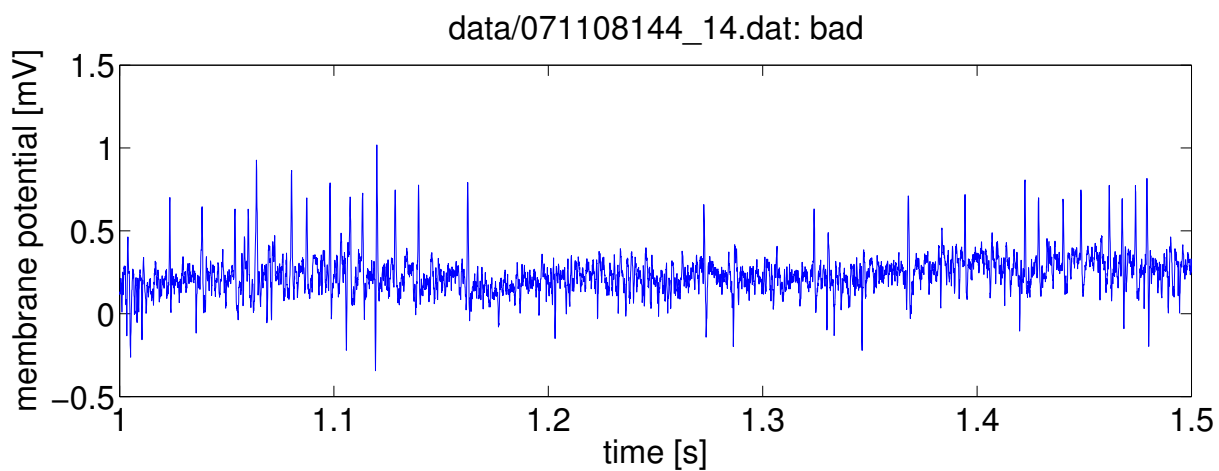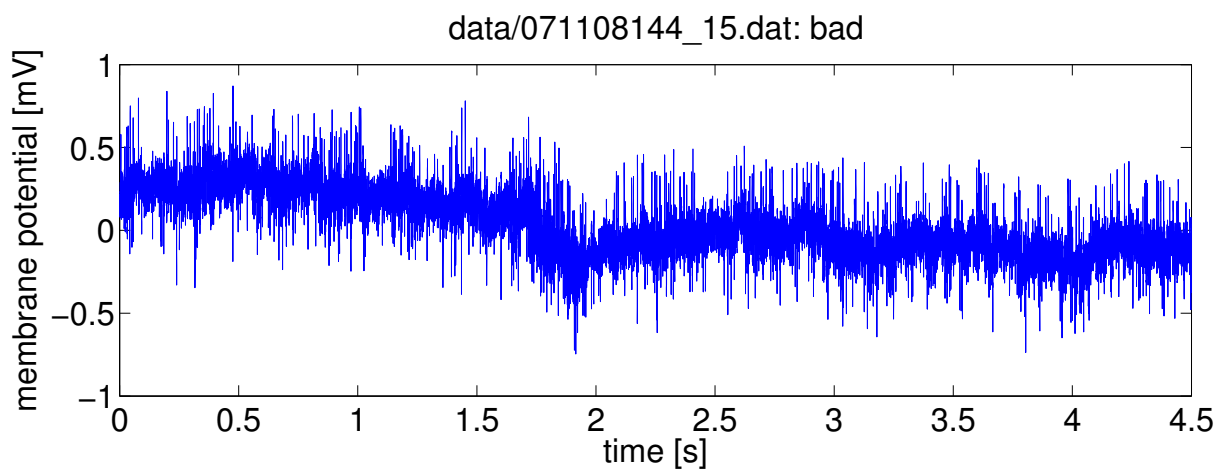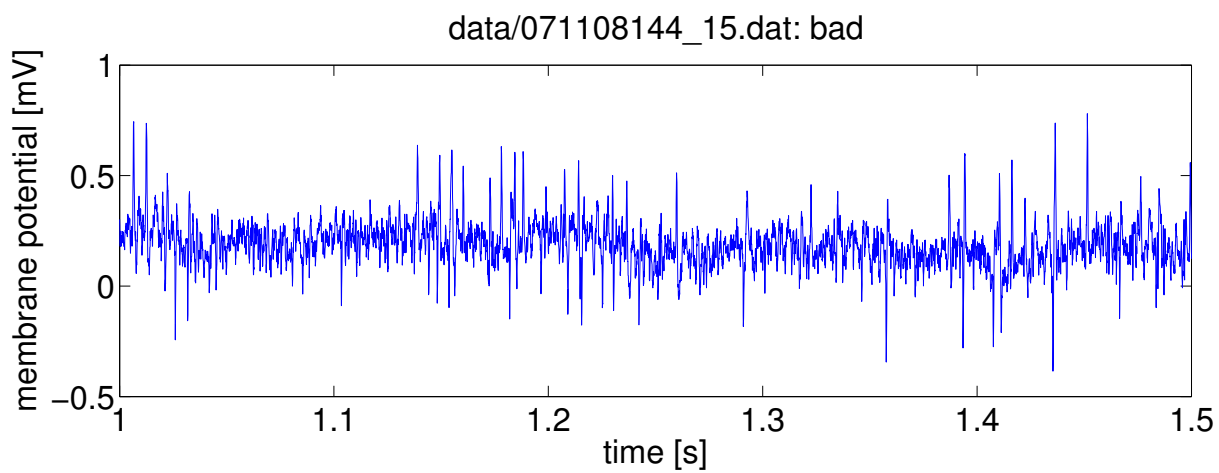

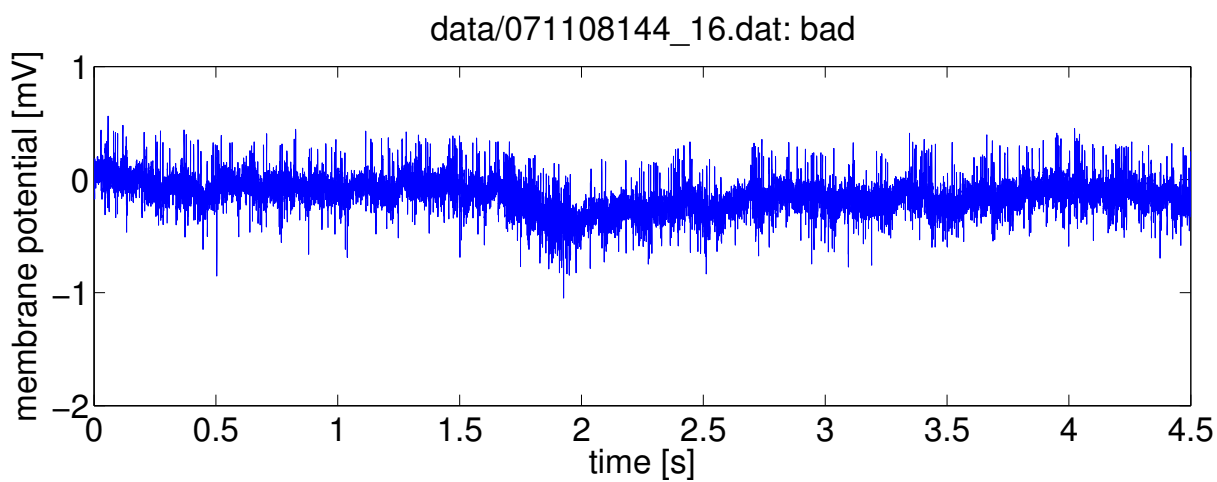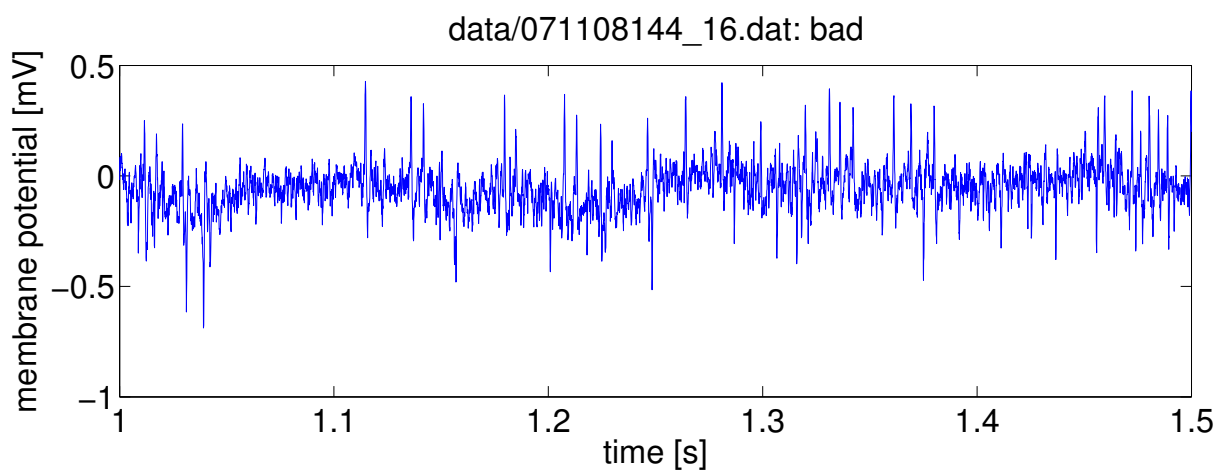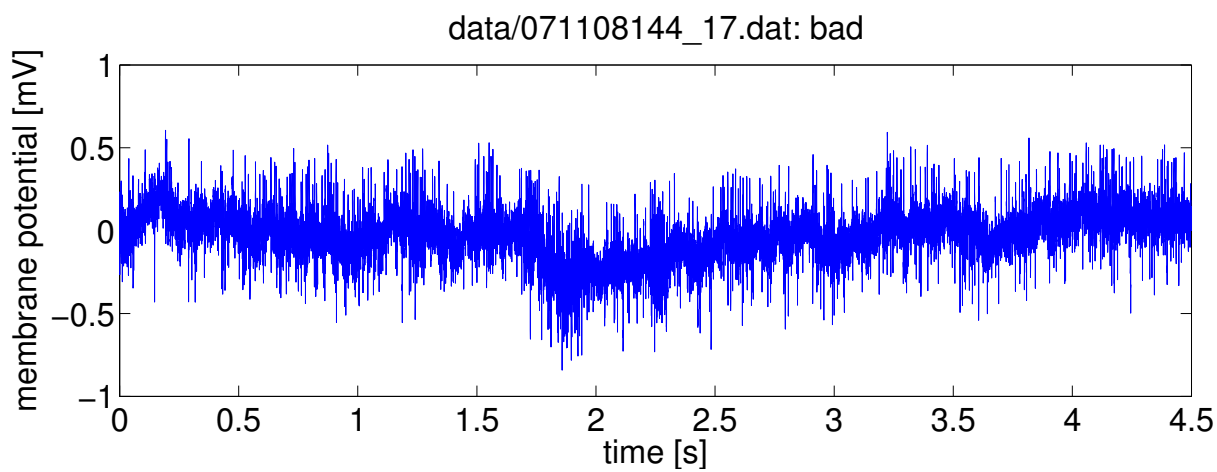

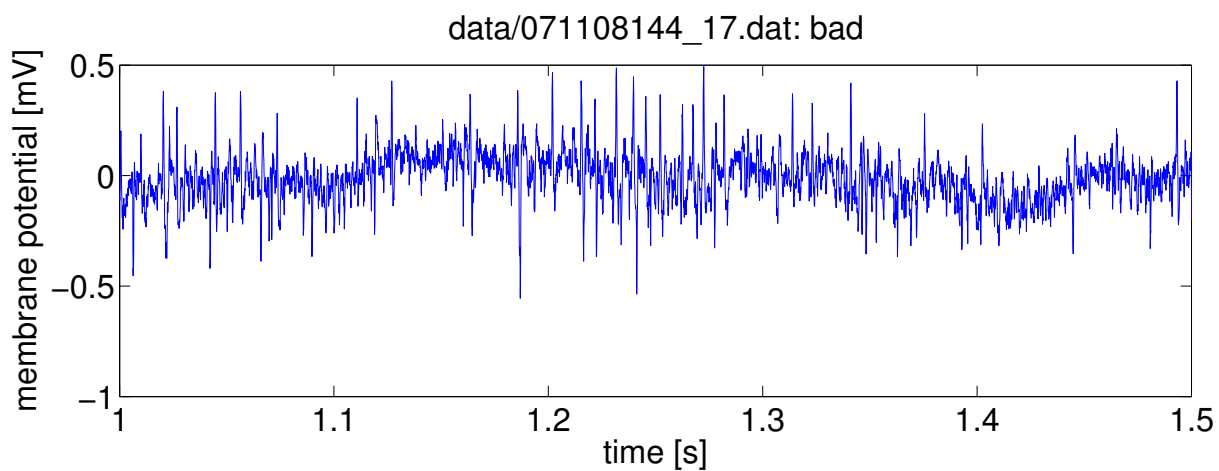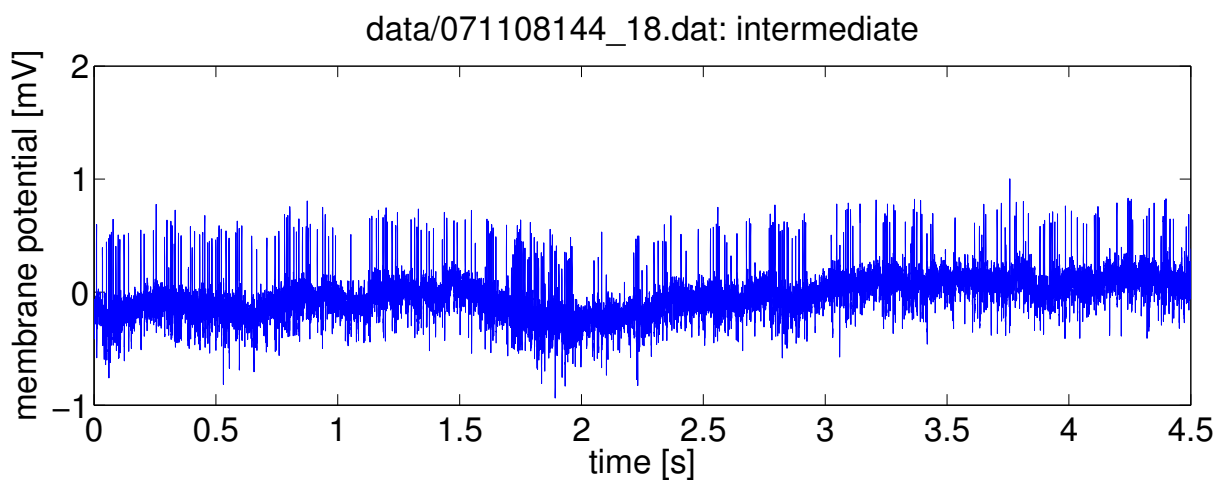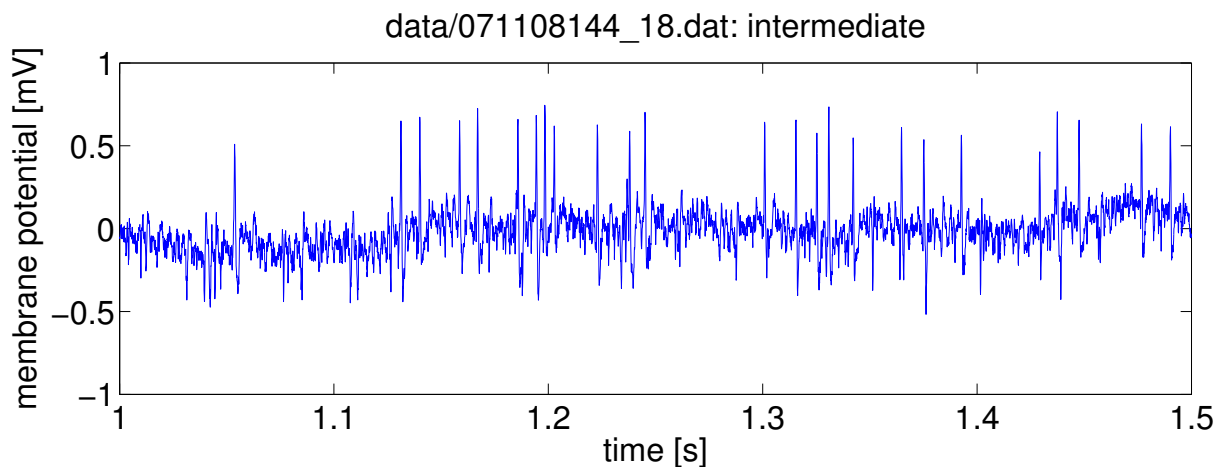

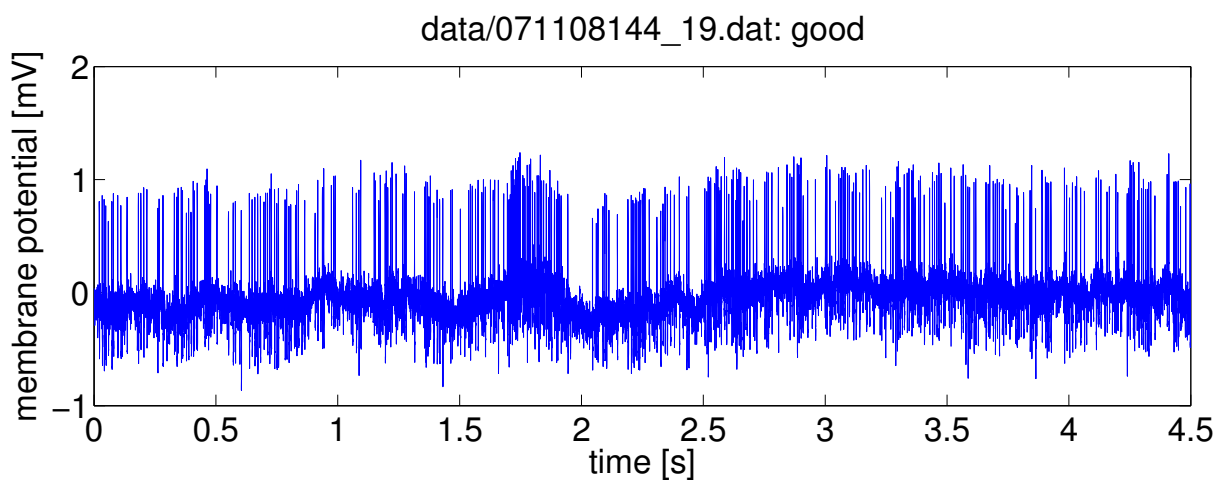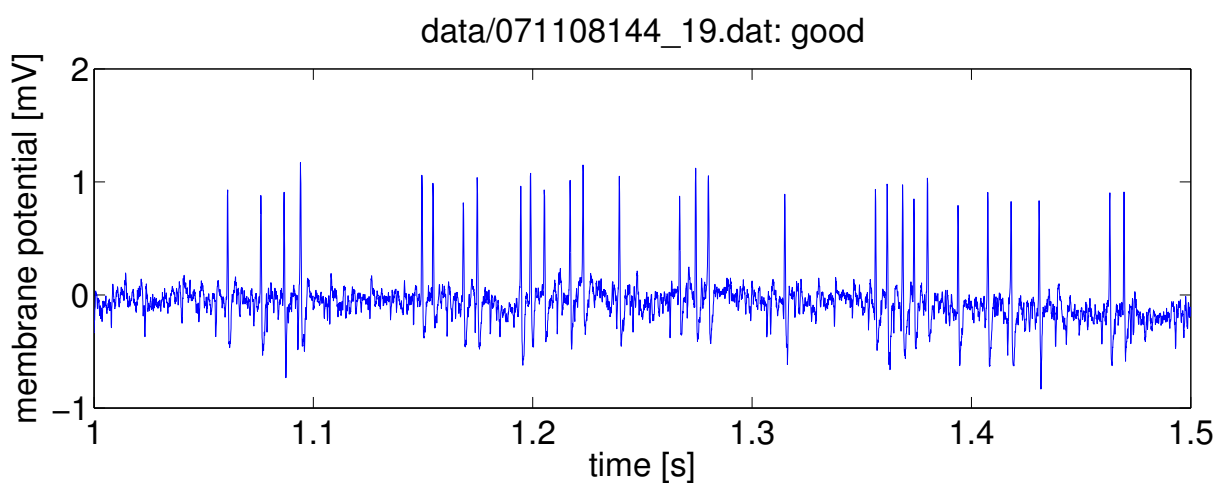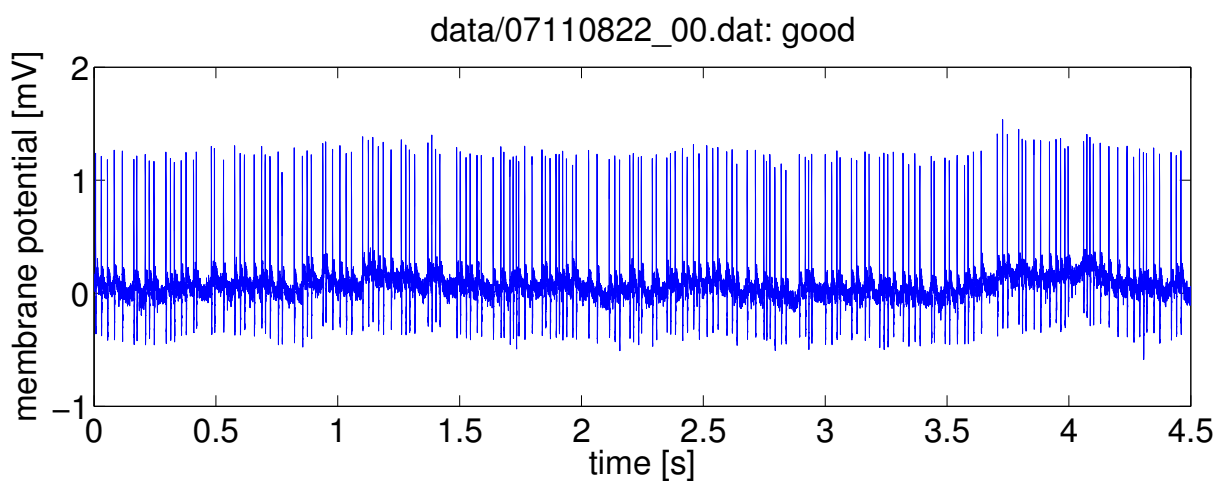

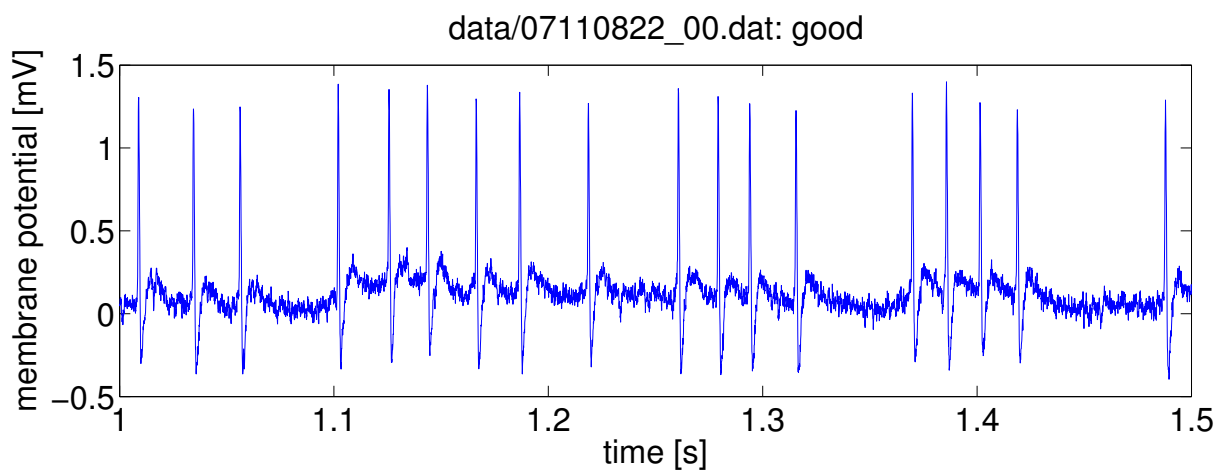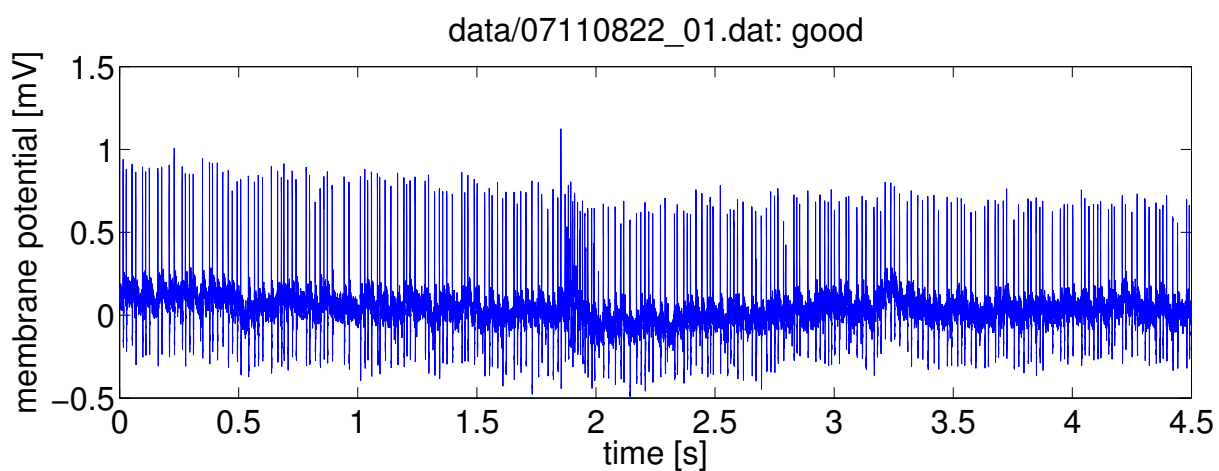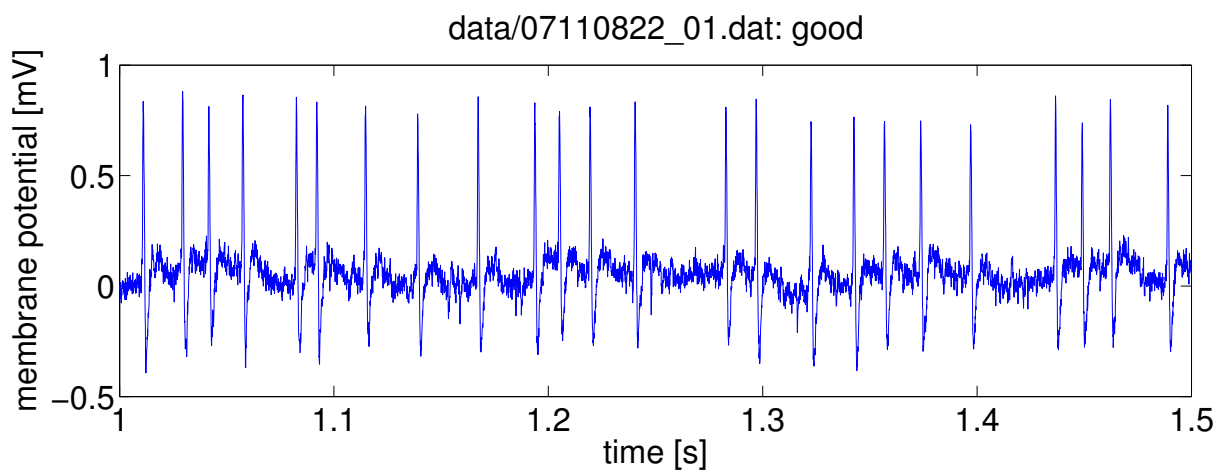

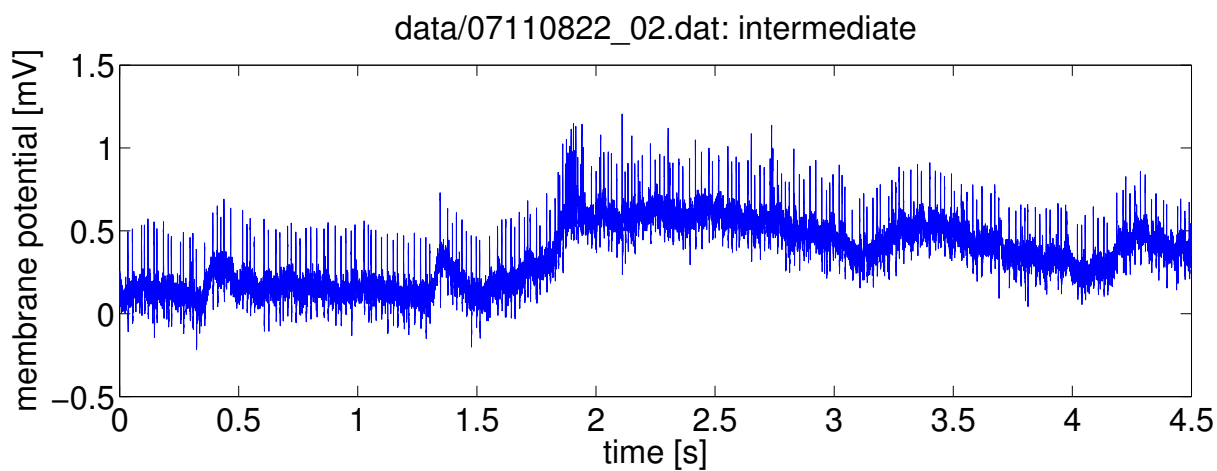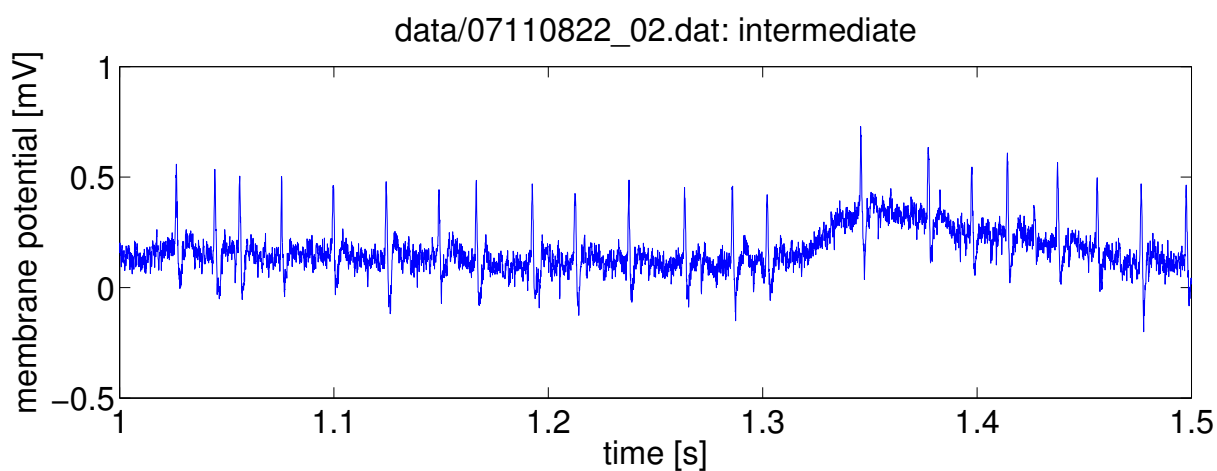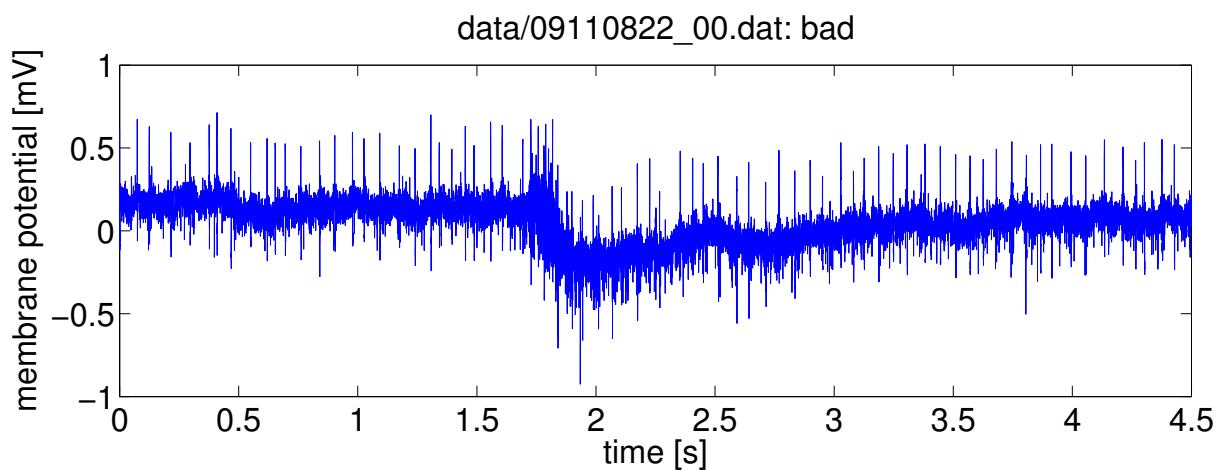

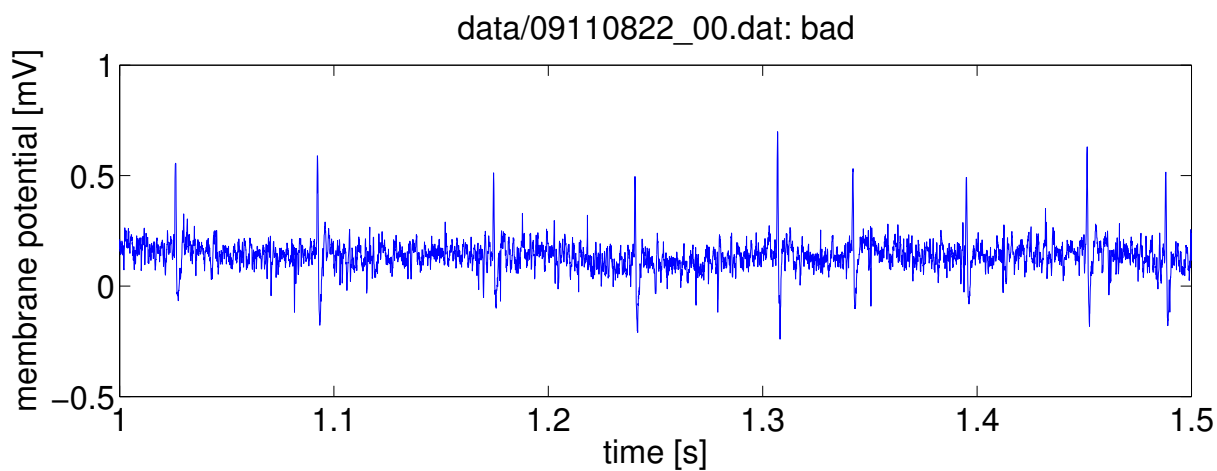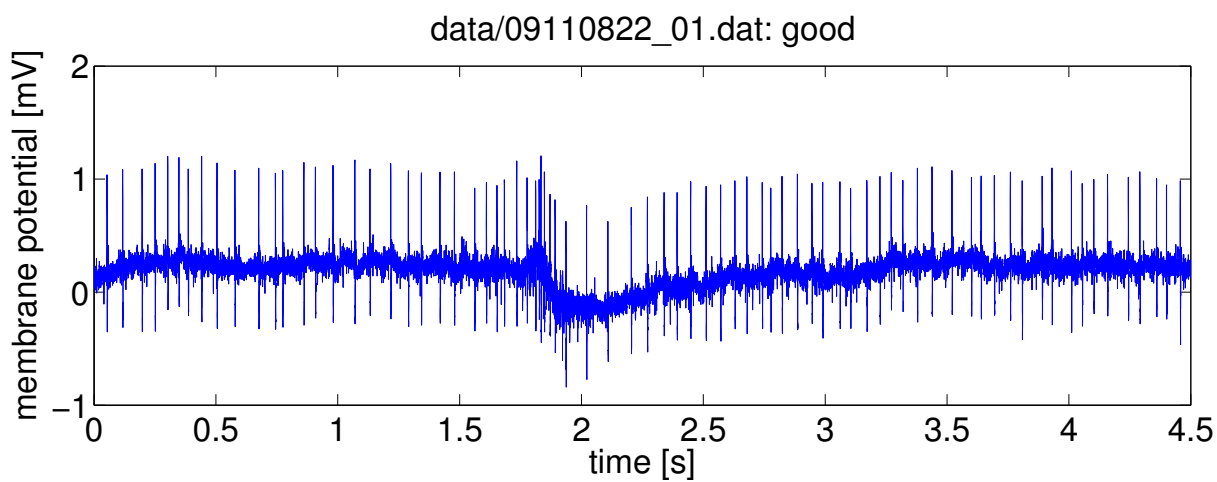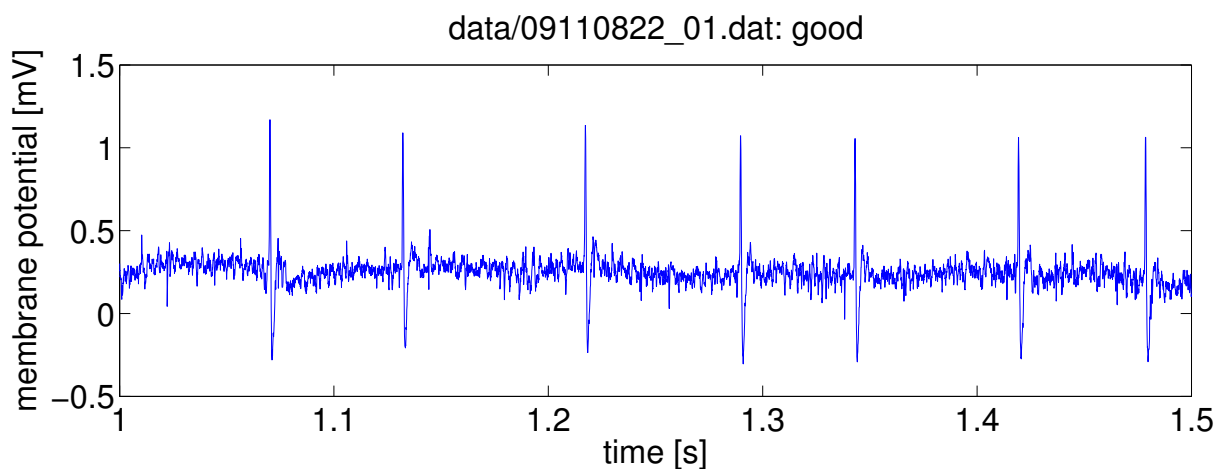

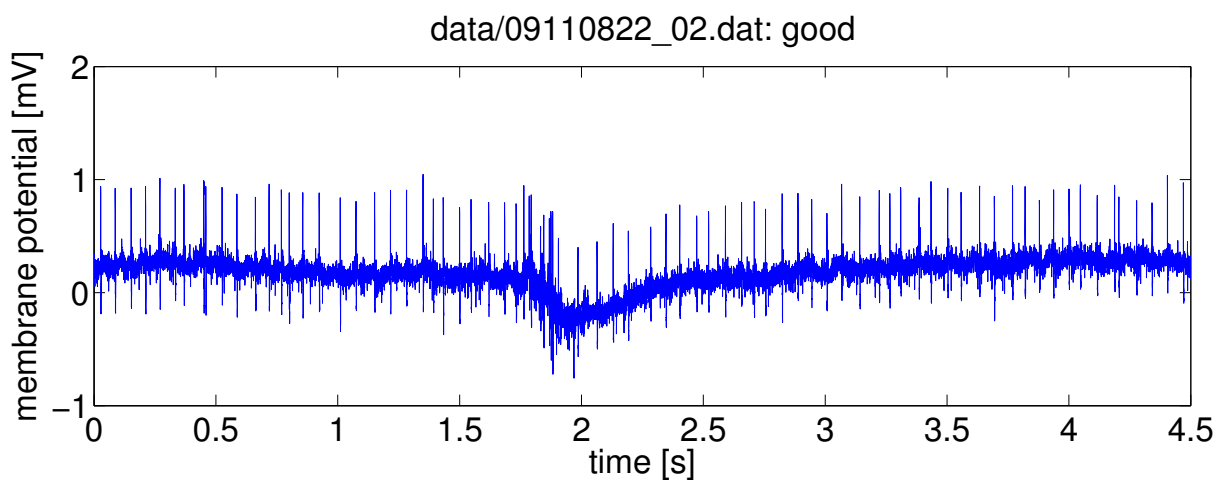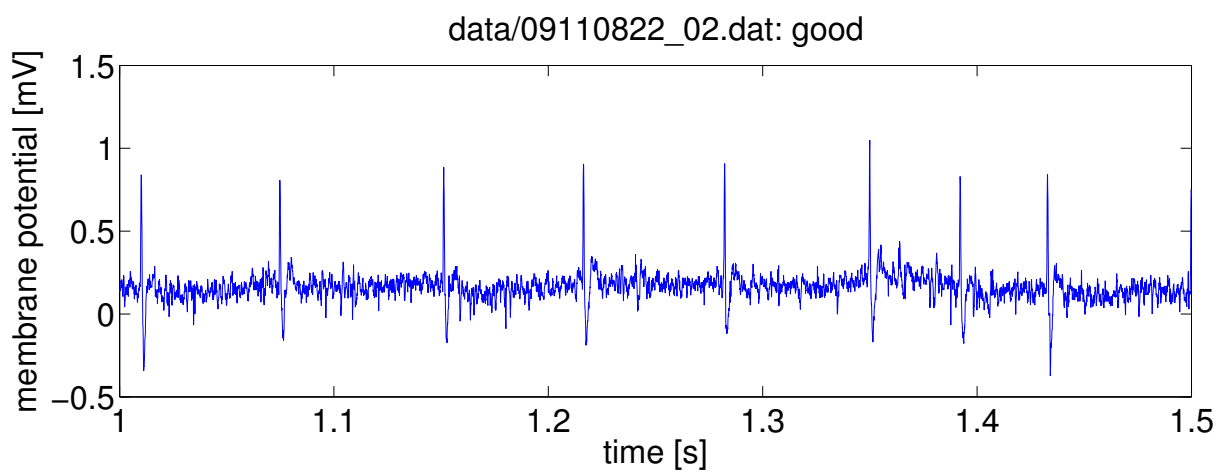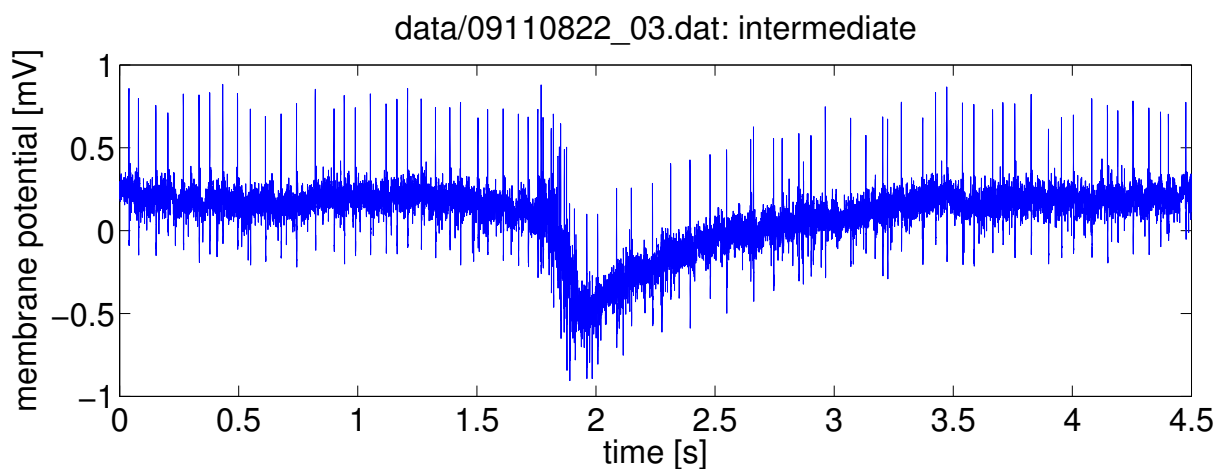

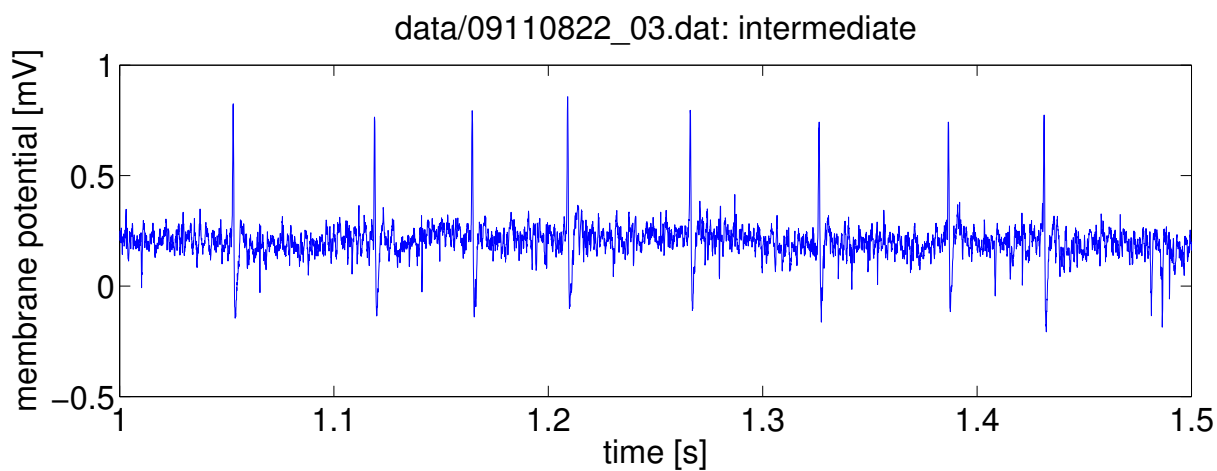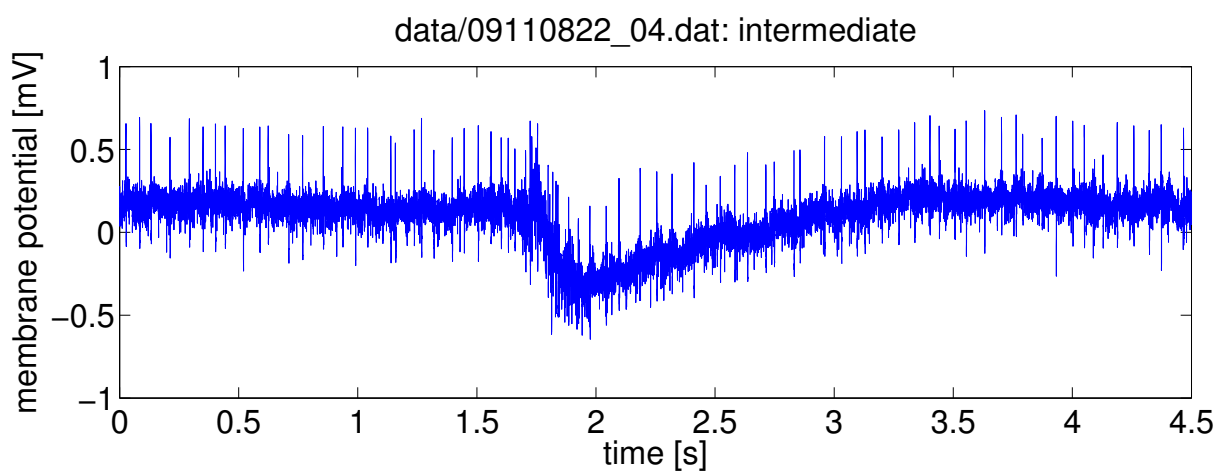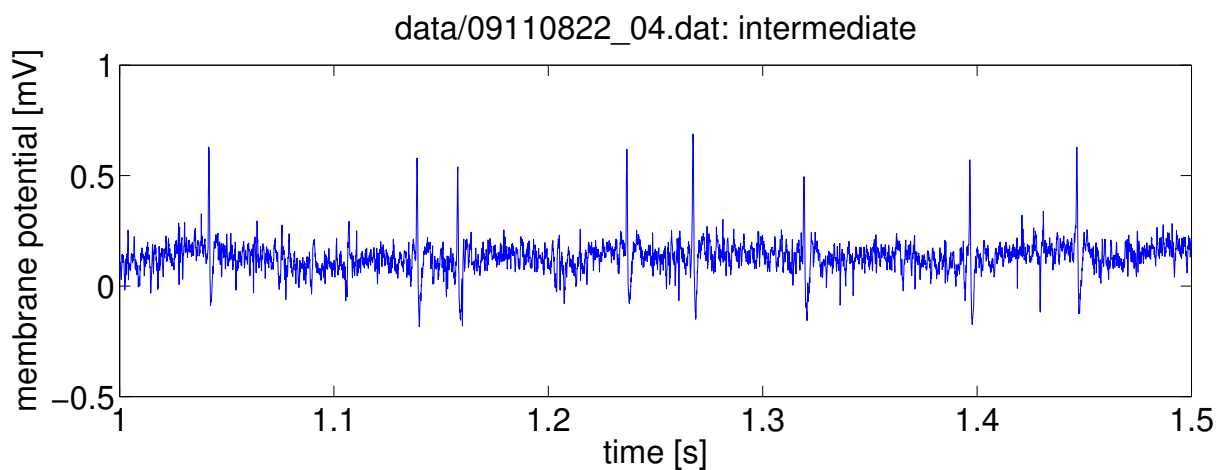

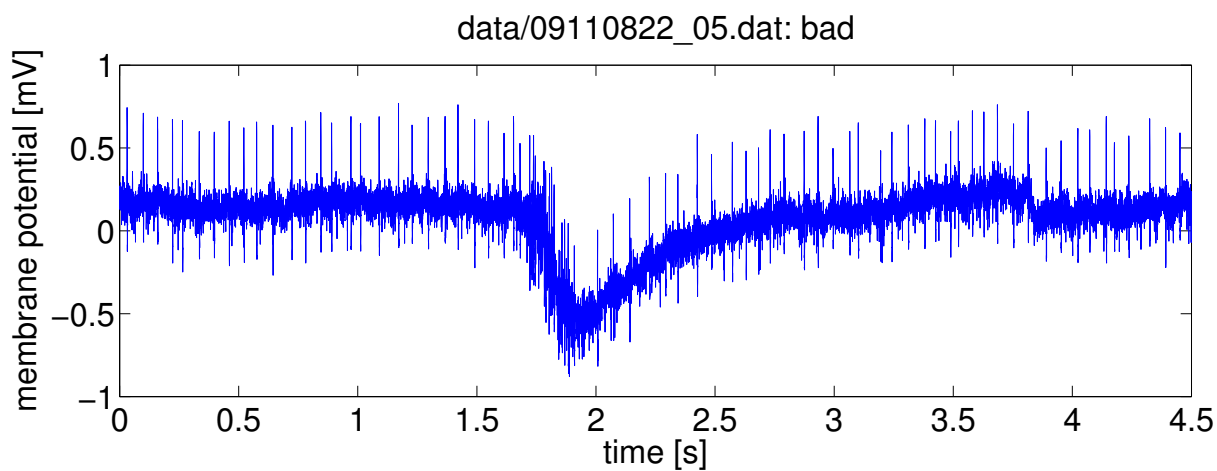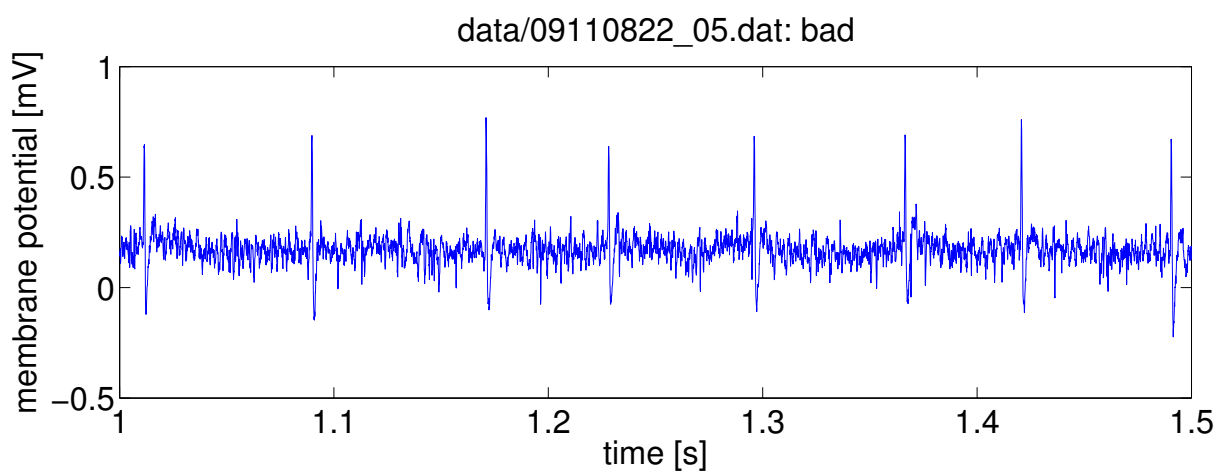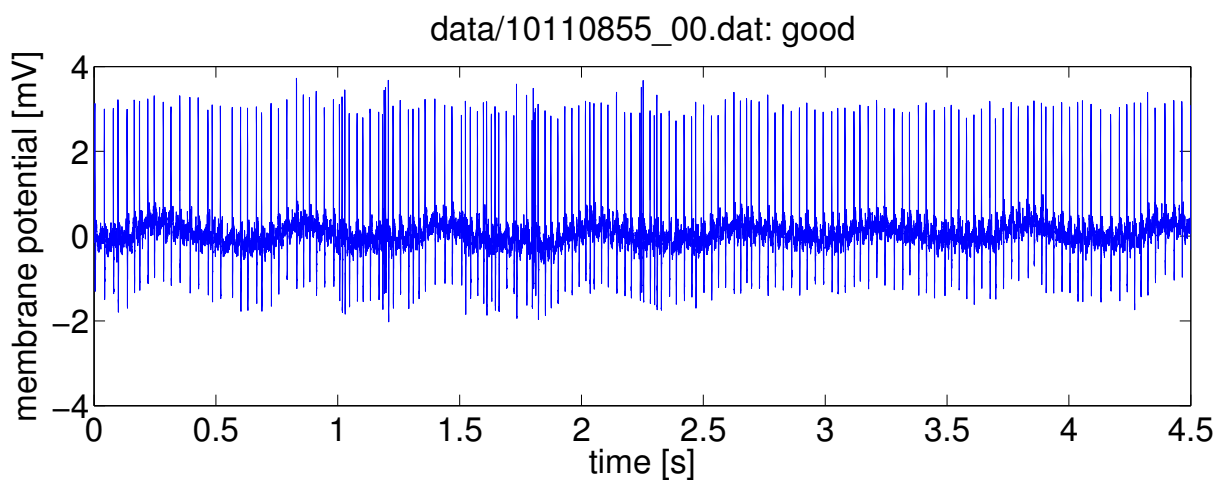

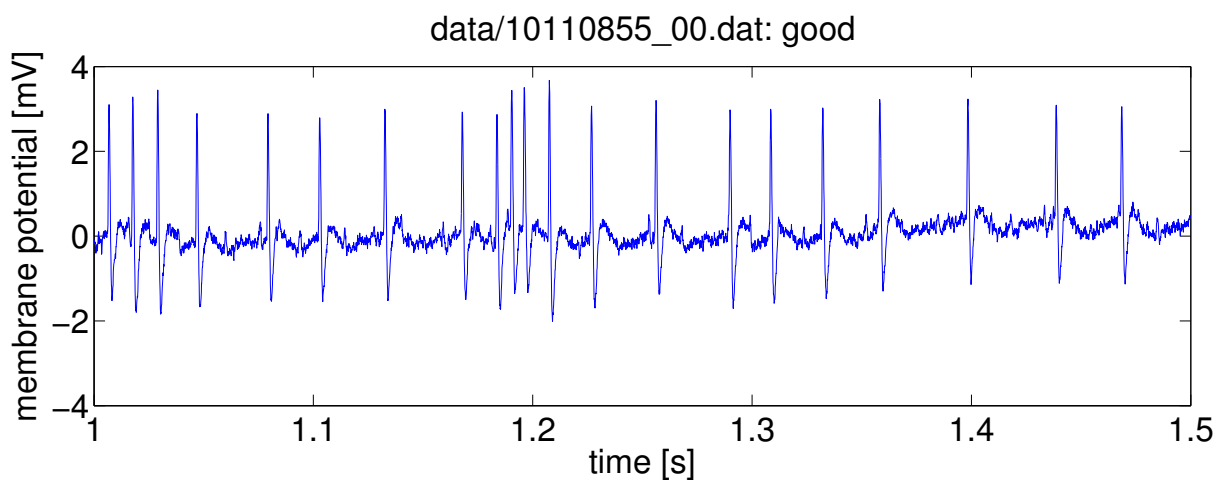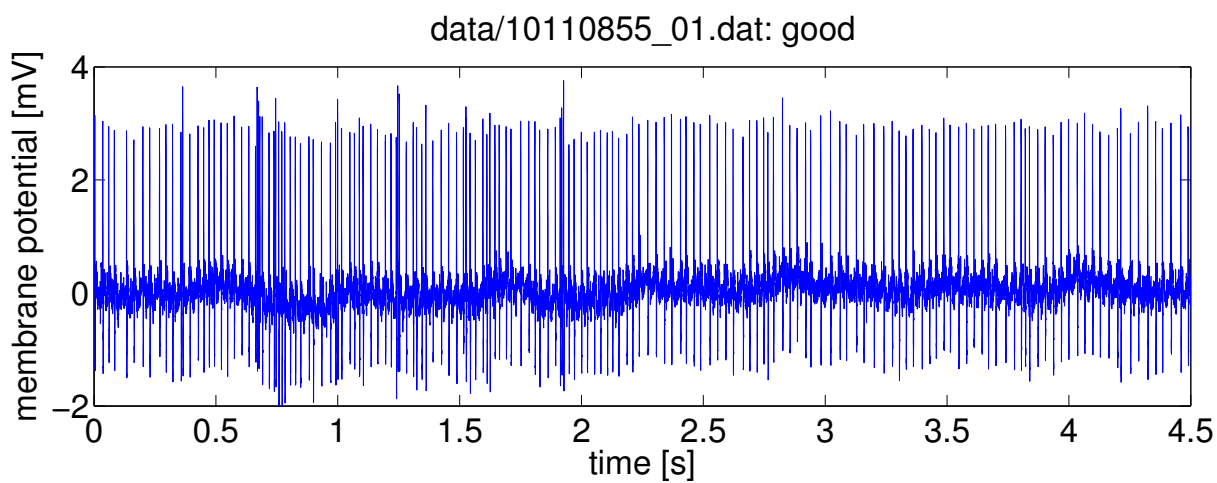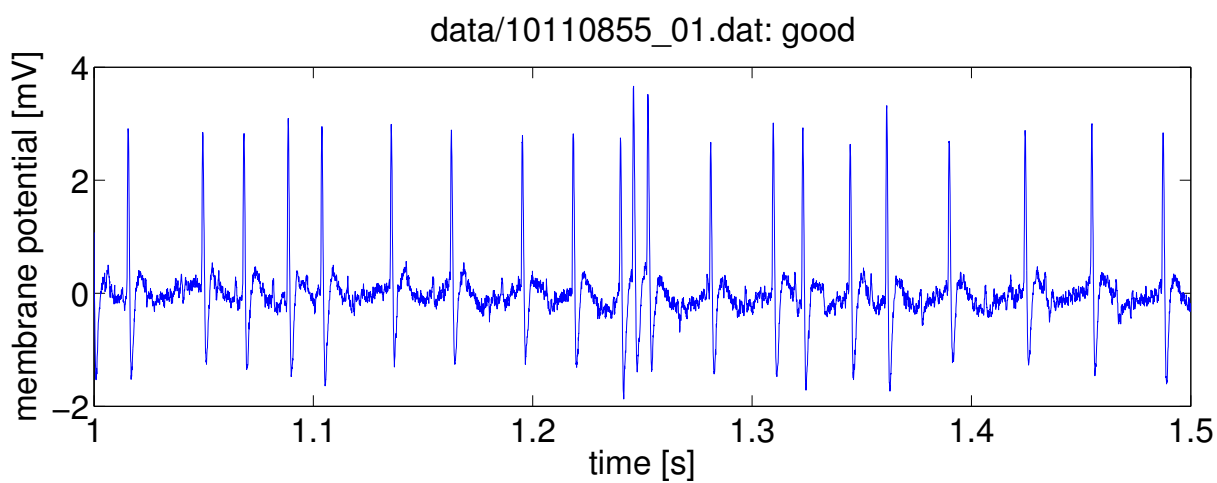

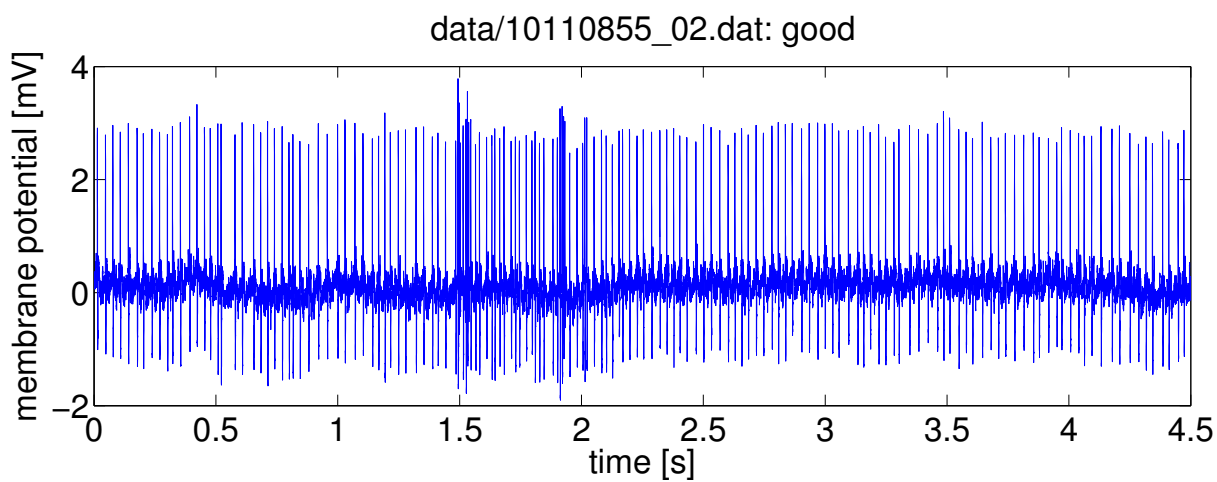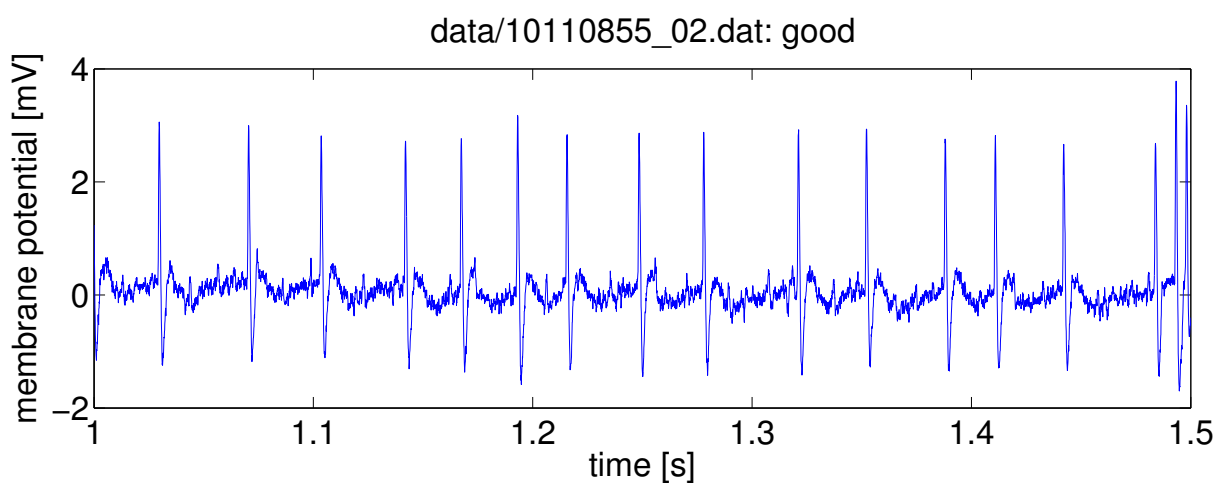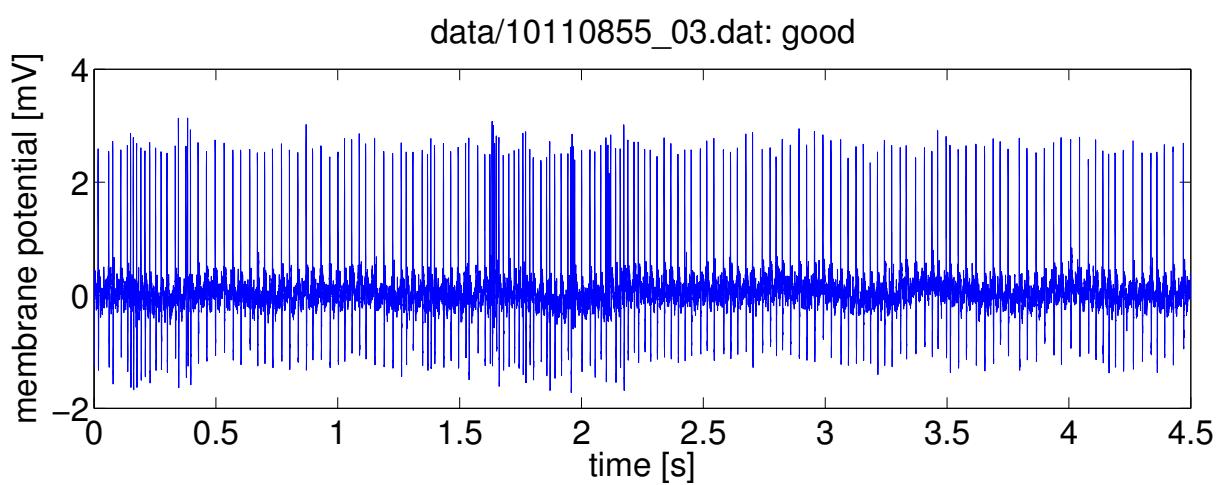

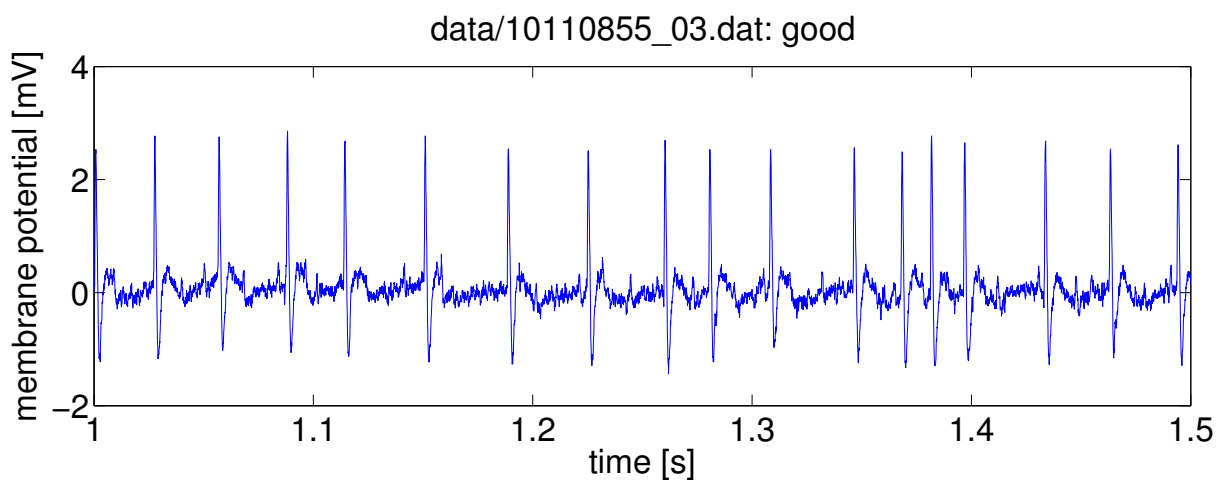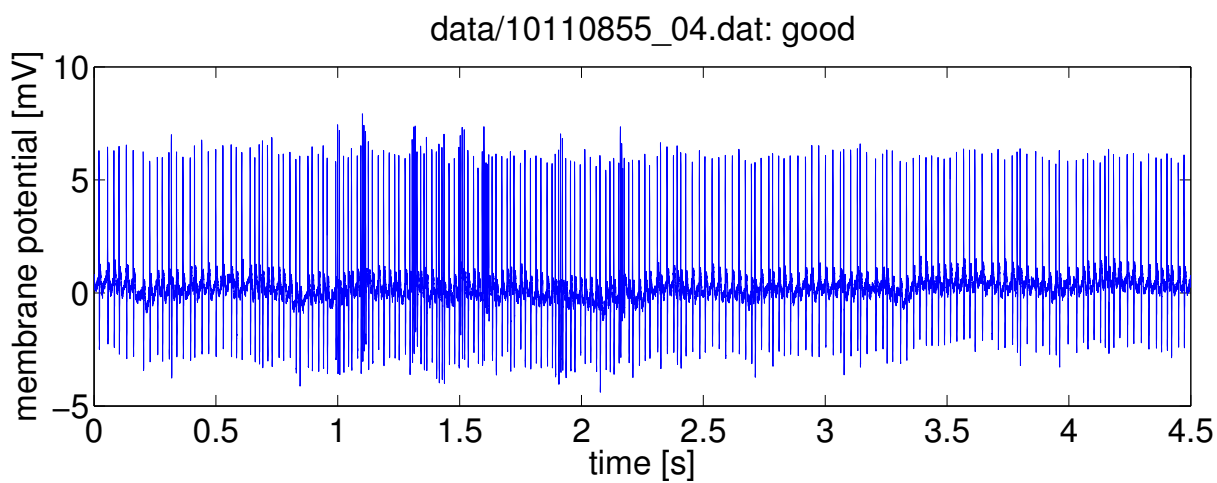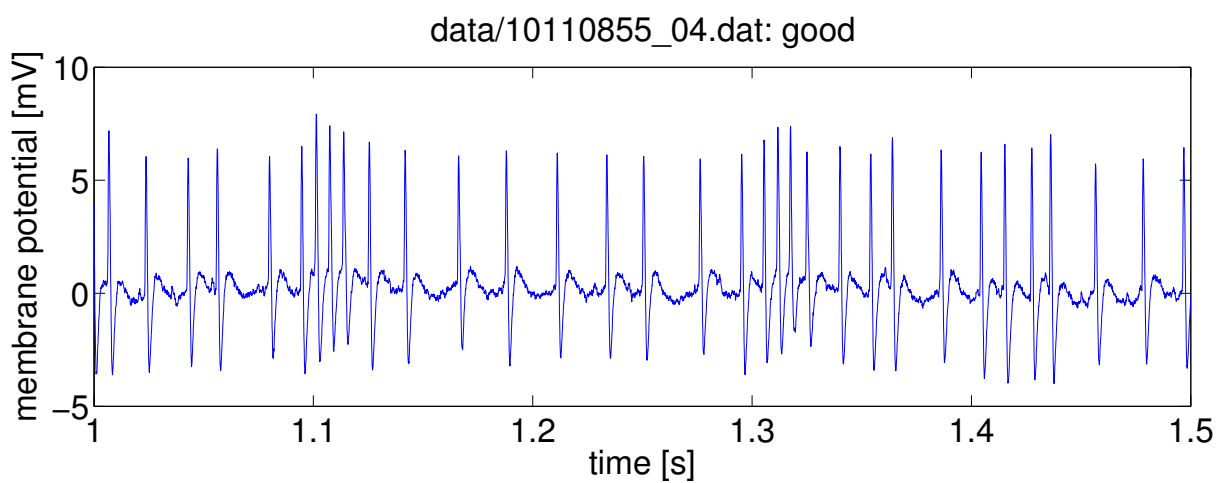

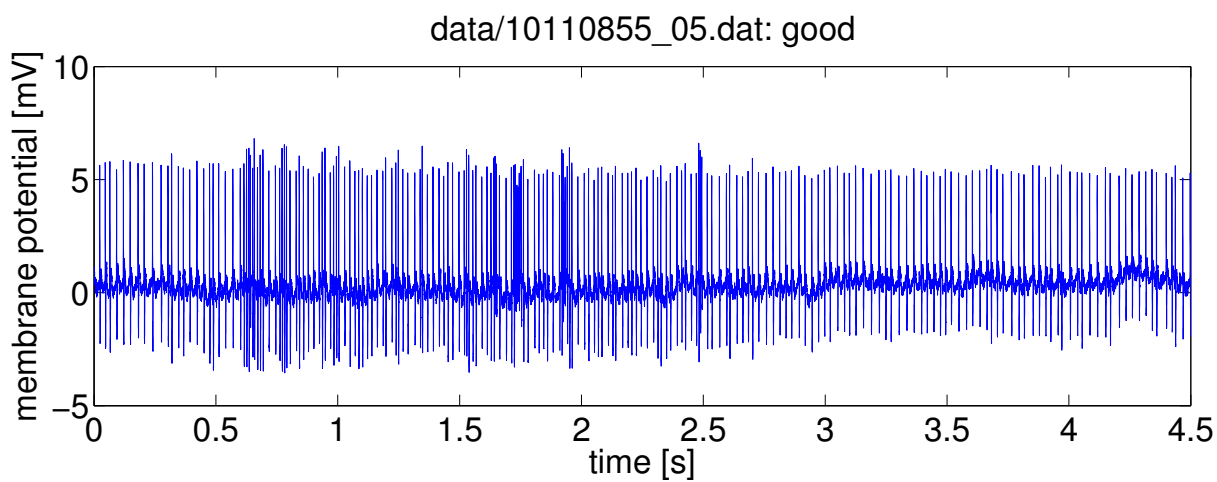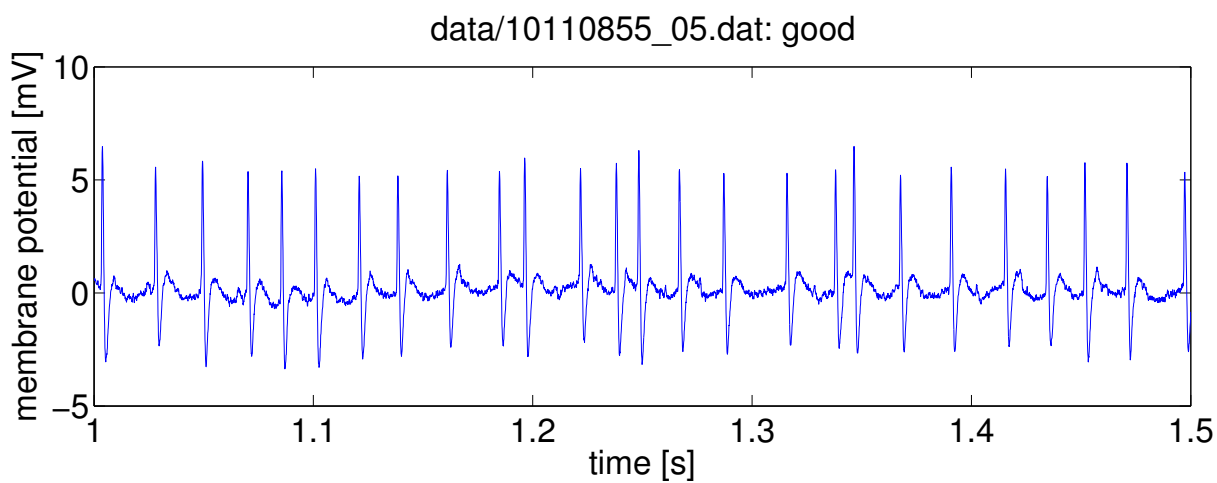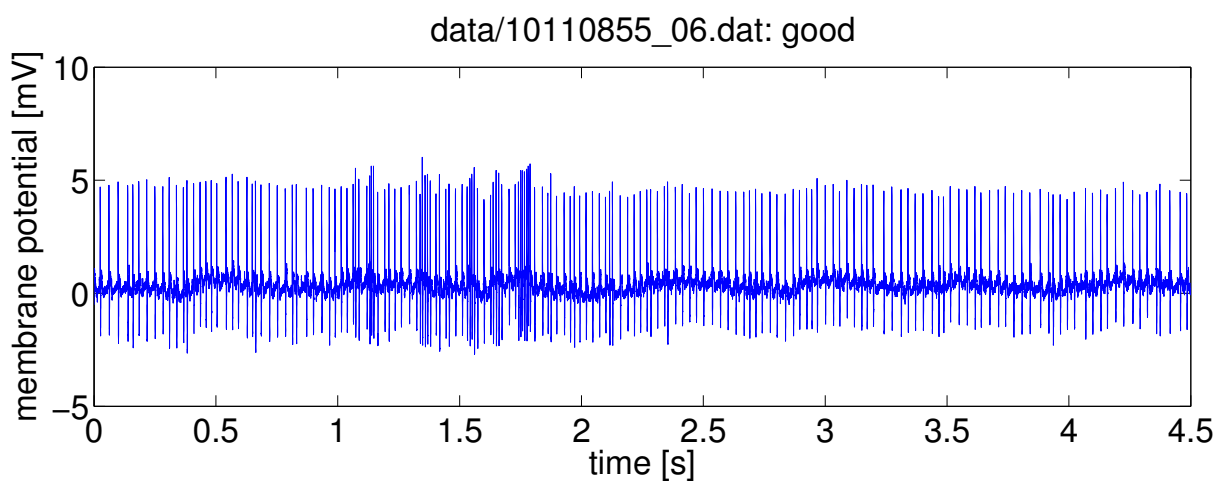

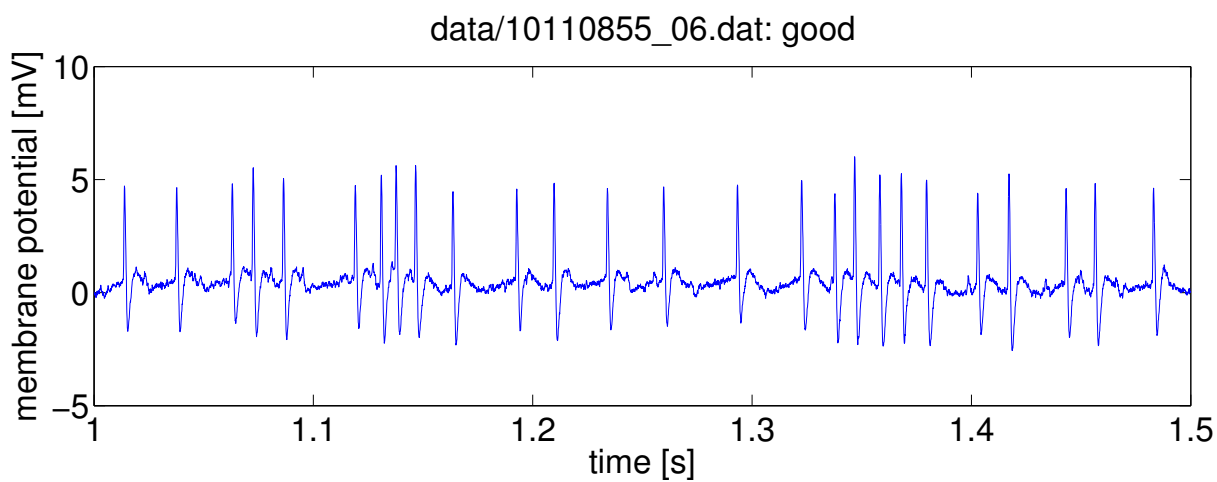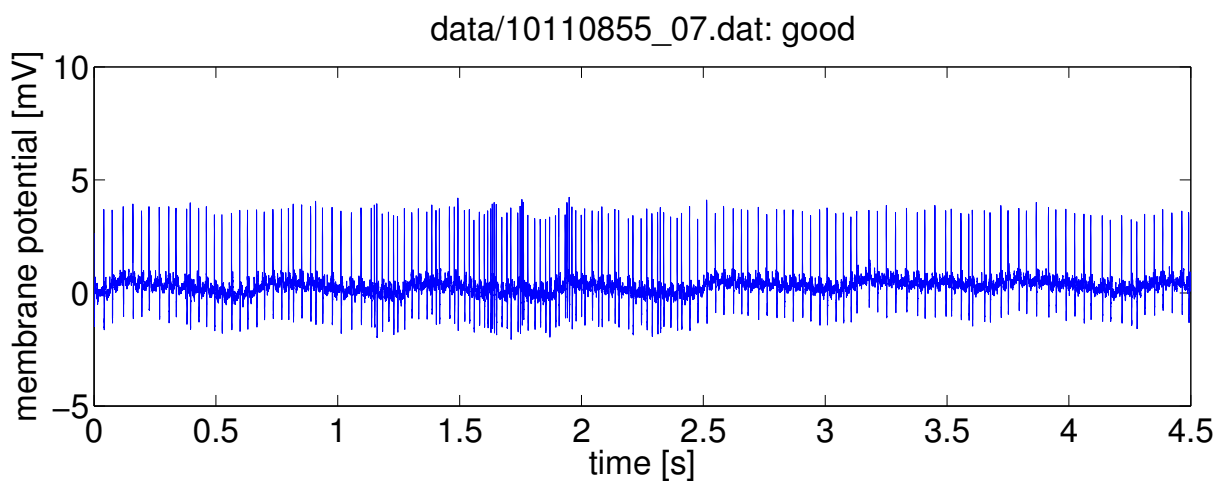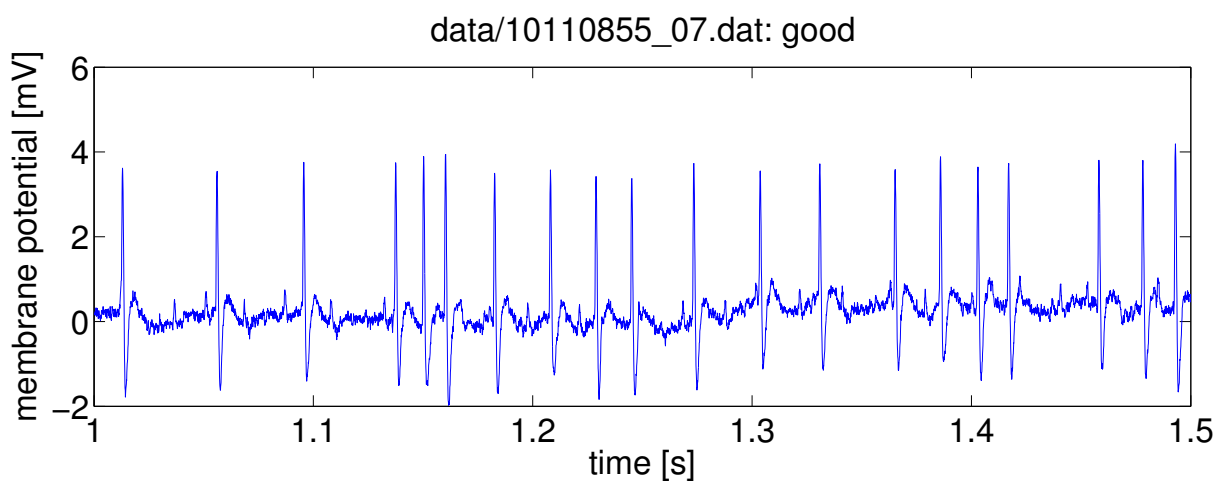

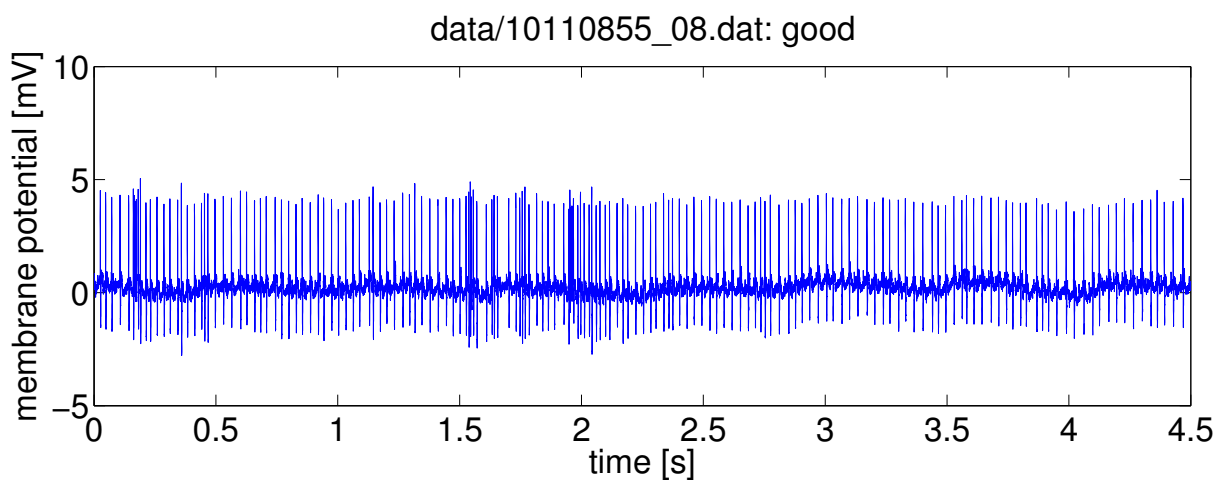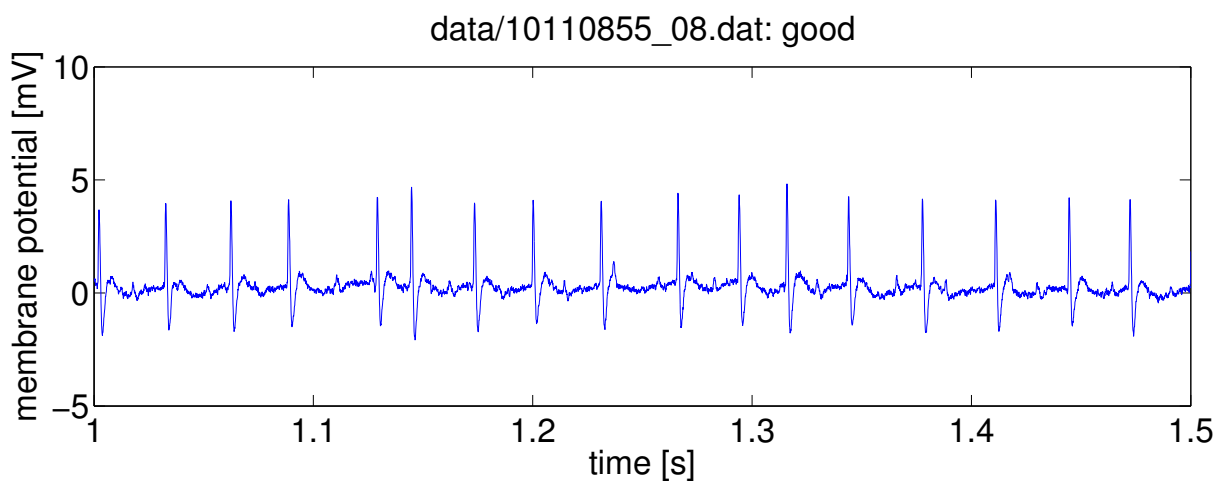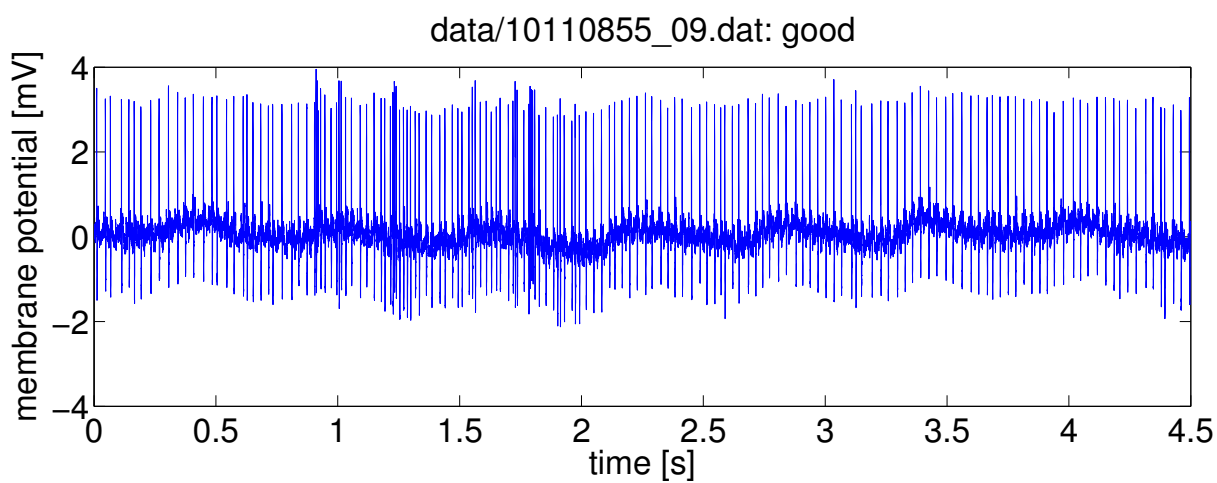

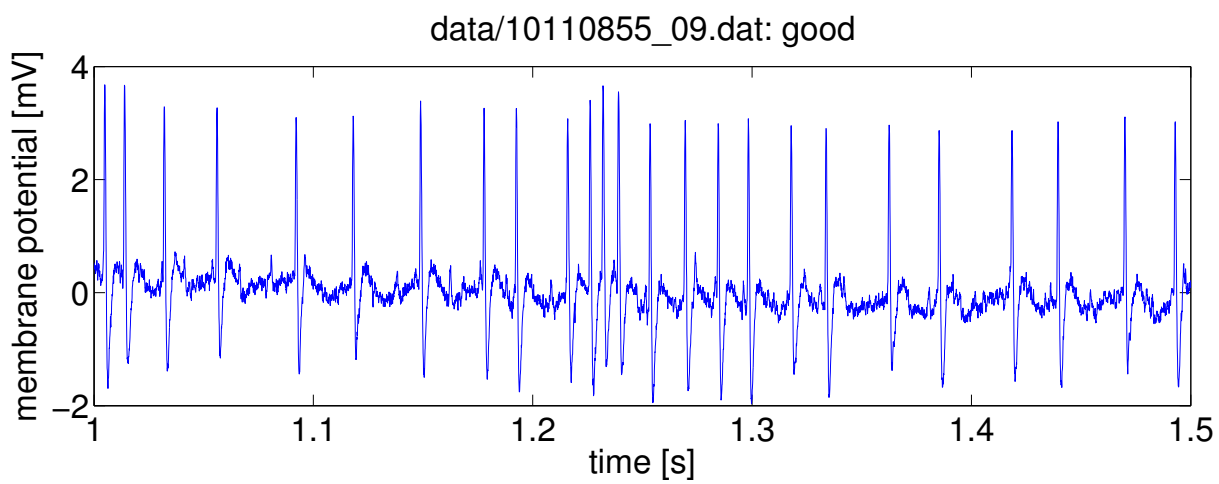

Supplement: Data S1 — PDF collection of example plots of the data used in our study. The data is displayed in its original unprocessed form and each plot is labelled with the corresponding file name of the original data files, which are included in Toolbox S1. (PDF) [file pone.0080838.s001.pdf]
